# The Big Picture of Neurodegeneration: A Meta Study to Extract the Essential Evidence on Neurodegenerative Diseases in a Network-Based Approach

**DOI:** 10.3389/fnagi.2022.866886

**Published:** 2022-06-27

**Authors:** Nicolas Ruffini, Susanne Klingenberg, Raoul Heese, Susann Schweiger, Susanne Gerber

**Affiliations:** ^1^Institute of Human Genetics, University Medical Center, Johannes Gutenberg University, Mainz, Germany; ^2^Leibniz Institute for Resilience Research, Leibniz Association, Mainz, Germany; ^3^Fraunhofer Institute for Industrial Mathematics (ITWM), Kaiserslautern, Germany

**Keywords:** neurodegenerative diseases, metastudy, multi-omic analyses, bioinformatics, hub genes and pathways

## Abstract

The common features of all neurodegenerative diseases, including Alzheimer's disease, Parkinson's disease, Amyotrophic Lateral Sclerosis (ALS), and Huntington's disease, are the accumulation of aggregated and misfolded proteins and the progressive loss of neurons, leading to cognitive decline and locomotive dysfunction. Still, they differ in their ultimate manifestation, the affected brain region, and the kind of proteinopathy. In the last decades, a vast number of processes have been described as associated with neurodegenerative diseases, making it increasingly harder to keep an overview of the big picture forming from all those data. In this meta-study, we analyzed genomic, transcriptomic, proteomic, and epigenomic data of the aforementioned diseases using the data of 234 studies in a network-based approach to study significant general coherences but also specific processes in individual diseases or omics levels. In the analysis part, we focus on only some of the emerging findings, but trust that the meta-study provided here will be a valuable resource for various other researchers focusing on specific processes or genes contributing to the development of neurodegeneration.

## Introduction

The typical features of all neurodegenerative diseases (NDDs), including Alzheimer's disease (AD), Parkinson's disease (PD), Amyotrophic Lateral Sclerosis (ALS), and Huntington's disease (HD), are the accumulation of aggregated and misfolded proteins and the progressive loss of neurons, leading to cognitive decline and locomotive dysfunction. Still, they differ in their ultimate manifestation (e.g., dementia, Parkinsonism, motor neuron impairment), the affected brain region [e.g., hippocampus (AD), Substantia nigra (PD), striatum (HD), upper and lower motor neurons (ALS)], and the kind of proteinopathy [amyloidosis, tauopathies (AD)], a-synucleinopathy (PD), CAG triplet elongation (HD) and TAR DNA-binding protein 43 (TDP-43) aggregates (ALS) (Roos, 2010; Zarei et al., 2015; Rosenberg et al., 2016; Armstrong and Okun, 2020; Pathak et al., 2021).

Adult neurons maintain energy-intense functions, such as membrane excitability, neurotransmission, and plasticity. They cannot rejuvenate by dividing, so they are especially reliant on a high supply of energy, the maintenance of protein and organelle quality control, rapid delivery of molecules within and out of cells, and the trafficking of organelles and other factors over considerable distances within the cell (Franco-Iborra et al., 2018; Gan et al., 2018). There are myriads of biological processes involved in the well functioning of the brain, whose malfunction can lead to neurodegeneration. Hallmarks among others are an aberrant cell cycle regulation, mitochondrial dysfunction, ER stress, impaired protein folding and quality control, deregulated autophagy and apoptosis, oxidative stress and insufficient DNA damage repair, missing homeostasis, a malfunctioning extracellular matrix, excitotoxicity, aberrant axonal transport, and excessive neuroinflammation (Coppedè and Migliore, 2015; Lindberg et al., 2015; Nah et al., 2015; Briston and Hicks, 2018; Cabral-Miranda and Hetz, 2018; Sonbol, 2018; Guzman-Martinez et al., 2019; Armada-Moreira et al., 2020; Guo et al., 2020; Joseph et al., 2020; Iatrou et al., 2021). To disentangle the multitude of processes associated with neurodegenerative diseases, we collected data from four omics-layers in four neurodegenerative diseases from 234 studies. These data are analyzed in a network-based approach that provides structure regarding the most represented processes, hub genes, and differences in regulation between specific omics-layers and diseases by building communities within networks for each omics-layer in each condition.

To provide an overview of the central biological processes and the pathways involved, we have divided them into broader categories according to their primary location or mode of action, namely, intracellular mechanisms, local tissue environment influences, and systemic influences (Ramanan and Saykin, 2013). On the cellular level, a deregulated cell cycle and misguided autophagy and apoptosis lead to degenerated neurons. Within the local tissue environment, an intact extracellular matrix (ECM), an unimpaired cell development, signal transduction, and transport of vital molecules are of fundamental importance. Excessive immune response and a dysregulated metabolism lead to significant disturbances on the systemic level.

Within our analyses, we found thousands of biological processes (BP) as defined by the Gene Ontology and local network cluster terms given by the string DB database. To allow an understanding within this complexity, we assigned those multilayered and diverse terms to 6 hallmarks: “cell cycle,” “autophagy/apoptosis,” “ECM/development,” “signal transduction/transport,” “immune system,” and “metabolism” for each community in all created networks. This observation allows us to extract processes distributed across diseases and omics-layers, which are mainly present in specific omics-layers or conditions. We only address the most striking results but see this work as an opportunity for other researchers working primarily on the effect of particular processor genes on neurodegeneration to study the gene communities associated with those processes in more detail. For example, a specific gene cluster containing the gene or process of interest can be extracted from the Supplementary Data sources we provide (see Supplementary Table 1—Genes Per Community) and inserted, e.g., in string DB (https://string-db.org/). The resulting network can be selectively examined, and all publications, processes, pathways, etc., that are significantly associated with the respective candidates or networks can be analyzed. Consequently, we hope the big picture of neurodegeneration we draw here in our results will serve as an inspiring and rich source of data, providing a range of new insights and analysis perspectives for the scientific community. In the following, we introduce the six hallmarks, providing a preview of our associated low-level terms by showing them in italics.

### Hallmark 1: Cell Cycle

Neuronal cells similar to muscle cells usually remain in a quiescent cell cycle state once they are differentiated due to their specialized functions. But cell cycle regulatory proteins like cyclins, CDKs, caspases, and p53 continue to be required for axonal migration, maturation, and survival (López-Sánchez et al., 2017; Joseph et al., 2020). One or more of these cell cycle proteins and pathways might get activated in response to various epigenetic or pathologic stimuli. Aberrant cell cycle reentry of neurons with duplication of the genetic material but without subsequent cell division is a well-known phenomenon. However, it is still unknown whether this is deleterious or beneficial. In the early stages of brain development, specific populations of neurons undergo incomplete mitosis and stay in a tetraploid state for the rest of their adult life. Still, a *de novo* neuronal tetraploidization in the adult brain seems to be an early indicator for neurodegeneration, with cells showing elevated molecular stress response and apoptotic marker proteins due to this aberrant *regulation of cell cycle* (Frade and Ovejero-Benito, 2015; López-Sánchez et al., 2017; Joseph et al., 2020). The correct assembly, positioning, and working of the mitotic *spindle apparatus* are essential for the development and differentiation of cells. Spindle orientation defects leading to an imbalance between symmetric and asymmetric divisions have been discussed as being linked to NDD. An asymmetric cell division leads to two daughter cells with different cell fates. An important example is the division of a stem cell, leading to a differentiated brain cell and another stem cell. The preconditions for an asymmetric cell division are the polarization of the content of the mother cell and the alignment of the mitotic spindle along the axis of polarity. If this process is disturbed by an impaired spindle orientation, this will have severe consequences in the development of the brain (Noatynska et al., 2012). During mitosis, motor proteins, together with Huntingtin, localize along spindle microtubules to segregate the chromosomes (Godin and Humbert, 2011). Thus, mutant Huntingtin could lead to defective neurogenesis during embryonic development leading to Huntington's disease later in life (Godin and Humbert, 2011; Wiatr et al., 2018). Defective *DNA repair* mechanism, together with elevated oxidative stress, is a typical hallmark for aging cells (Joseph et al., 2020). As the brain is the largest consumer of oxygen, reactive oxygen species (ROS) are the main inducers of DNA damage in neurons (Nissanka and Moraes, 2018). The accumulation of unrepaired damaged DNA might contribute to a cell cycle reentry of the neuron to increase polyploidy, thus compensating for the loss of information on some parts of the DNA. Also, general terms like *transcription (mitochondrial) translation, RNA modification, spliceosome*, and *chromatin* organization were defined as subprocesses integrated into the hallmark called the cell cycle.

Obviously, transcription, translation, and chromatin remodeling as major contributors to protein homeostasis have a considerable impact on all cellular functions, and genetically or epigenetically misregulated transcription factors can enhance or weaken all the processes that lead to or prevent neurodegeneration like neuroinflammation, oxidative stress, and protein homeostasis (Berson et al., 2018; Jin et al., 2019). Especially neurons face the problem that their locus of transcription is distant from the locus of translation as protein synthesis is decentralized to meet the rapidly changing metabolic needs of axons and dendrites. Nuclear mRNA is transported from the cell body to the periphery as RNA/protein granules linked to motor proteins of the cytoskeleton by adaptor complexes. Conditions, such as mislocalization of the mRNA, a defective cytoskeleton, and deleterious adaptor complexes, lead to synaptic dysfunction, loss of synapses, and, ultimately, to neuronal death (Liu et al., 2017).

### Hallmark 2: Autophagy and Apoptosis

Accurate folding and control over levels of many proteins are tightly regulated in neuronal cells by a protein quality control network consisting of molecular chaperones and an intact degradation system of misfolded or discarded proteins, consisting of the ubiquitin-proteasome system and *autophagic processes*. *Chaperone*-*mediated processes* are involved in the promotion of *de novo* folding of nascent proteins or the refolding of misfolded polypeptide chains, thereby preventing their aggregation (Hartl et al., 2011; Chaplot et al., 2020).

Proteins that cannot be folded correctly or are no longer needed are tagged by the attachment of the small protein ubiquitin (*ubiquitinylation*) and degraded by a multi-subunit protein complex called the *proteasome* or by autophagy, which is a universal lysosome-dependent intracellular degradation process for not only protein aggregates but also cell organelles and cytoplasmic constituents to maintain cell homeostasis (Nah et al., 2015; Schmidt et al., 2021). Lysosome-dependent autophagy can either be carried out by lysosomes acting by themselves in a process called micro-autophagy, or in macro-autophagy where cell organelles with a lipid double layer derived from the ER called omegasomes fuse with lysosomes to form autophagosomes (Mizushima and Klionsky, 2007; Li et al., 2012; Nah et al., 2015). In neurons, these autophagosomes are generated constitutively in the cell periphery and mature on their way to the cell body (Xie and Klionsky, 2007). The proteins ensuring this axonal transport are Huntingtin and Huntingtin-associated protein 1. As huntingtin is predominantly cleared by autophagy, the deficiency of intact huntingtin in HD is a double stress factor leading to the accumulation of polyglutamine-expanded huntingtin polyQ-htt and other cytoplasmic constituents that need to be degraded (Wong and Holzbaur, 2014). Besides deficiencies in the protein quality control network leading to the typical protein aggregations in NDD, there are many more factors that impair homeostasis of the cell, causing stress to the cells. Heat, toxins, mechanical damage, infections, starvation, hypoxia, and oxidants all lead to cellular *stress responses* that aim to control the induced damage and, if possible, to confer resilience to the stressor. The integrated stress response (ISR) is an elaborate signaling cascade, where different stress signals activate different protein kinases that converge in phosphorylating the core of the ISR, the α-subunit of the translation initiation factor 2 (eIF2α). This leads to a reduction of global protein synthesis in favor of ISR-specific mRNAs, such as the activating transcription factor ATF4. ATF4 is the main effector of the ISR. It forms homo- and heterodimers that bind to DNA targets to control the expression of genes involved in cellular adaptation, mainly chaperones that refold denaturated proteins or tag them for immediate degradation (Harding et al., 2003; Fulda et al., 2010; Pakos-Zebrucka et al., 2016). To terminate the integrated stress response, dephosphorylation of eIF2α is required. However, dephosphorylation of eIF2α can also facilitate the production of death-inducing proteins in cases where the cell is so severely damaged that normal functioning and homeostasis cannot be restored (Pakos-Zebrucka et al., 2016). Highly regulated cascades of events will then lead to the decomposition of the cell in a process called apoptosis (Danial and Korsmeyer, 2004; Elmore, 2007; Fulda et al., 2010). Apoptosis is a caspase-mediated programmed cell death that can be triggered either by stress signals from within the cell or by signals that are released by the surrounding cells and that bind to cell-surface death-receptors (Danial and Korsmeyer, 2004; Elmore, 2007). Both intrinsic and extrinsic pathways ultimately induce cell death by activating caspases, enzymes that carry out the degradation of the cell, leading to cell shrinkage, plasma membrane blebbing, chromosome condensation and degradation, mitochondrial death with release of cytochrome C, cellular fragmentation, and formation of membranous apoptotic bodies. At the same time, phosphatidylserine residues are exposed at the cell surface to attract macrophages that phagocytize the cell fragments. The extrinsic pathway is activated by extracellular ligands (e.g., CD95 ligand, tumor necrosis factor α) binding to cell-surface death receptors, which either leads to the formation of the so-called DISC (death-inducing signaling complex) or the activation of nuclear factor kappa B (*NF*κ*B*) (Wang and El-Deiry, 2003; Lavrik et al., 2005). Caspase 8, which serves as the initiator caspase, is activated within this complex. If the activating stimulus is sufficiently large, caspase 8 begins activating the effector Caspases 3, 6, and 7. They translocate to the mitochondria to trigger pore formation in the outer mitochondrial membrane (Fan et al., 2005), which ultimately leads to mitochondrial permeability and death. The release of cytochrome C promotes caspase-9 activation, which triggers a cascade of proteolytic events. The intrinsic signaling pathway is controlled by the Bcl2-family of proteins and is triggered by cell-internal factors, such as DNA damage, osmotic stress, or growth factor withdrawal. The triggering cell event activates the transcription factor p53, leading to the expression of proapoptotic Bad protein. The Bad protein, in turn, activates the multidomain proapoptotic Bax protein, which triggers permeabilization of the mitochondrial outer membrane. As a result, similar to the extrinsic pathway, cytochrome C escapes from the intermembrane space of the mitochondria and induces a proteolytic cascade (Mcilwain et al., 2013; Wang and Tjandra, 2013; Chen et al., 2018).

The *NFkB signaling pathway* is activated during apoptosis either to enforce the ongoing apoptotic processes or to navigate the cell back to the survival pathway by stimulating the expression of anti-apoptotic genes, especially *TRAF1* and *TRAF2*, and thus overruling the activities of the caspases (Sheikh and Huang, 2003). NFκB is a family of transcription factors with multiple physiological and pathological functions. The NFκB complex exists in an inactive state in the cytoplasm. It consists of different NFkB dimers and inhibitory kB proteins (IκB) that prevent NFkB to enter the nucleus and bind to the DNA (Israël, 2010). NFkB can be activated by highly variable stimuli, including growth factors, cytokines like tumor necrosis factor alpha and interleukin 1-ß, bacterial and viral antigens like lipopolysaccharides or double-stranded RNA and physicochemical insults like ionizing radiation or free radicals. All these different stimuli activate different phosphorylation cascades that, in the end, lead to an interaction with the IκB kinase (IKK) complex, which then leads to the phosphorylation of IκB in the NFkB complex. Once phosphorylated, IκB gets ubiquitinylated and degraded by the proteasome. The released NFkB can now enter the nucleus to activate target gene expression that regulates immune responses, cell growth, and proliferation but also apoptosis (Qin et al., 2007). The selectivity of the NF-κB response is based on several factors, including dimer composition, timing, organization of chromatin, and cell type (Natoli, 2009; Sen and Smale, 2010). Besides the *NFkB signaling pathway*, an important low-level term in autophagy and apoptosis, was the *mTOR signaling pathway*. As mentioned above, an impaired autophagy in NDD leads to the accumulation of toxic protein aggregates that promote cellular stress and death. The kinase mammalian target of rapamycin (*mTOR*) is a central regulator of metabolism as it senses cellular nutrient, oxygen, and energy levels (Perluigi et al., 2015). It is a potent repressor of autophagy and reacts to food abundancy with cell growth and proliferation, whereas, in times of starvation, it is inhibited, thus allowing the cells to derive energy from autophagic and apoptotic processes (Heras-Sandoval et al., 2020).

### Hallmark 3: Extracellular Matrix Organization and Development

The extracellular matrix (ECM) is a three-dimensional network that reaches all cells of the body to provide structural support, segregate tissues, and regulate intercellular communication by many different biochemical processes. It consists of minerals and macromolecules like *collagen*, elastin, enzymes, glycoproteins, and hyaluronan, which is most abundant in the brain (Bonnans et al., 2014). These compounds contribute to a varying degree of stiffness and elasticity of the ECM throughout the body from hard bone to soft brain tissues. The mechanical properties of the ECM environment can be sensed by the cells, which is the precondition for processes like *cell migration*, proliferation, *differentiation and growth*, apoptosis, and, on a higher level, the development of tissues and organs (Bonneh-Barkay and Wiley, 2009; Sethi and Zaia, 2017; Maguire, 2018). These processes also require a direct connection between cells and ECM. The ECM communicates with the cells by connecting to the cytoskeleton through *cilia* and *(focal) adhesion complexes* (Geiger and Bershadsky, 2002). The cytoskeleton is a functionally versatile filamental structure that transports and *localizes* the contents of the cell, it organizes the process of cell division, namely, chromosome segregation and cytokinesis, it connects the cell physically and biochemically to the external environment through interacting with the ECM (*cell adhesion, focal adhesion, cell junction*), and it enables the cell to move and change shape (*cell migration, cell differentiation, growth, and localization*) (Wozniak et al., 2004; Fletcher and Mullins, 2010). These functions are mediated by three filament types: microtubules, actin microfilaments, and intermediate filaments, which, in neurons, are called neurofilaments (Herrmann et al., 2007; Hohmann and Dehghani, 2019). Microtubules are hollow tubes consisting of tubulin dimers, which are stabilized by tau proteins. They grow out from the centrosome to the plasma membrane by constantly adding and subtracting tubulin dimers at both ends of the filament (Mitchison and Kirschner, 1984). They are responsible for the intracellular transport together with the motor proteins kinesin and dynein that transport, e.g., vesicles, nutrients, and essential organelles, but also broken-down neurotransmitters and misfolded proteins by extending along the axon and functioning as the cytoskeletal navigation pathways in the transporting process (Guo et al., 2020). The hyperphosphorylation of tau proteins as seen in AD results in microtubule destabilization and cytoskeleton abnormalities (Mandelkow et al., 1995; Barbier et al., 2019). Actin ß- and γ- monomers, together with formin homology proteins, form linear polymers called actin microfilaments, which can then be arranged as a network with the help of the proteins Arp2 and Arp3. The Arp2/3-complex forms dimers that resemble actin dimers, but are much more stable. They attach themselves in a 70° angle and serve as nucleation cores for actin filaments activated by the nucleation-promoting factor proteins of the WASP/WAVE-family (Kurisu and Takenawa, 2009). Both filaments and networks stabilize the plasma membrane of the cells and control the position of the nucleus and other cell organelles (Pfisterer et al., 2017). They maintain and adapt cell shape, thus allowing for cytokinesis, organization of *cell junctions*, and enhancement of *cell adhesion*. C*ell migration* is achieved by the polymerization of new actin filaments in the forward edge of a moving cell that pushes the cell membrane forward in protrusions called lamellipodia. These protrusions form mechanical links between the actin filaments and the ECM called *focal adhesions*. Once attached, the rear of the cell body contracts, squeezing its contents forward past the adhesion point. Once the adhesion point has moved to the rear of the cell, the cell disassembles it, allowing the rear of the cell to move forward (Ananthakrishnan and Ehrlicher, 2007). The actin filaments also provide the tracks for cellular transport, allowing actin-based motor proteins (myosins) to transport organelles over short distances.

The interactions of *a*-actin und myosin but also troponin and tropomyosin are most sophisticated in muscle cells. These highly specialized cells work together to contract and release muscles, thus leading to movement of the whole organism and to the *cardiac muscle action potential* (Cooper, 2000). Intermediate filaments are the major cytoskeletal proteins, which, in contrast to microtubules and microfilaments, are more heterogeneous, have tensile strength, and do not participate in cell motility (Herrmann et al., 2009). The intermediate filaments primarily provide structural support for the cell, especially for the long neuronal cells where they regulate the diameter of the axon and, thereby, the nerve conduction speed (Helfand et al., 2003).

All cells have intermediate filaments, but the protein subunits of these structures vary. They can be classified into six different types: Types I and II are the *keratins*, which are expressed in epithelia. Type III contains the proteins vimentin, which frequently colocalizes with microtubules, desmin, peripherin, and glial fibrillary acidic protein (GFAP). Type IV consists of the neurofilament proteins L, M, H, and internexin. Type V consists of the nuclear lamins, and Type VI consists of the protein nestin (Muñoz-Lasso et al., 2020). During embryonic development of the brain microtubules, microfilaments and neurofilaments work together to guarantee proper growth and guidance of axons; during adult life, they maintain neuronal homeostasis and plasticity. Given these fundamental processes rely on an intact cytoskeleton organization, it is not surprising that cytoskeleton defects, including alterations in microtubule stability, in axonal transport as well as in actin dynamics, have been characterized in several NDDs.

### Hallmark 4: Signal Transduction and Transport

Neuronal signal transduction and transport of organelles and vital molecules along the axon maintain neuronal activity and health. In NDD, neuronal signaling is more and more impaired because of synaptic and cytoskeletal dysfunctions. The loss of synapses has detrimental consequences as they are critical to learning, memory, and behavior (Lepeta et al., 2016; Ardiles et al., 2017), and perturbations in axonal transport can lead to neuronal cell death (Perlson et al., 2010).

*Cell communication* in the brain is achieved by *neuronal signaling via synapses*. In this process, electrical activity is transferred from one neuron to another through neurotransmitters as chemical mediators. The most important neurotransmitters in the central nervous system are glutamate for the excitatory, and GABA and glycine for the inhibitory synapses, but also dopamine, serotonin, and acetylcholine (Fogarty et al., 2016). Action potentials that travel along axons induce an intracellular Ca^2+^ influx mediated by *voltage*-*gated calcium channels* at the presynaptic terminal. Increased Ca^2+^ levels are sensed by the synaptic vesicle protein synaptotagmin I, which induces the Soluble NSF Attachment Protein Receptor (SNARE) protein complex to mediate fusion of the neurotransmitter vesicles with the plasma membrane (*SNARE binding*), releasing them into the synaptic cleft (*neurotransmitter secretion*). The neurotransmitters then bind to and thus *activate receptors* of the postsynaptic cell, which can be either *ion channels* or G protein-coupled receptors. They transmit signals from the post-synaptic membrane to the cell body by transducing the chemical signal into an electrical signal that depolarizes the postsynaptic cell and is transmitted downstream to the next neuron (Lepeta et al., 2016; Taoufik et al., 2018).

Rather than being just a gap, the synaptic cleft is full of trans-synaptic adhesion molecules that not only physically connect the pre- and post-synaptic compartment but also mediate recognition and signaling processes that are essential for the establishment, specification, and plasticity of synapses. Such synapse-organizing adhesion molecules include neurexins and neuroligins, cadherins, integrins, receptor phosphotyrosine kinases, and phosphatases, such as ephrins and Rho GTPases (Missler et al., 2012; Jang et al., 2017). Deficiencies in the complex synaptic functioning are not only seen in neurodegenerative diseases but also in neuropsychiatric disorders, suggesting that the loss of synapses is not the endpoint incident of the disease but, rather, a triggering event (Taoufik et al., 2018).

*Axonal transport*, in general, is a process where the motor proteins kinesin and dynein loaded with diverse cargos navigate along the microtubules of the cytoskeleton. Anterograde axonal transport delivers proteins, lipids, mRNA, and mitochondria to the distal synapse, whereas retrograde transport clears misfolded or aggregated proteins and brings distal trophic signals to the soma (Millecamps and Julien, 2013; Sleigh et al., 2019).

*Transmembranal transport* systems in neurons are the *anterograde ER to Golgi transport* with COP II-coated *vesicles* and the *retrograde trans-Golgi-Network* transport with COP I-coated *vesicles* (Martínez-Menárguez et al., 2019; Wang L. et al., 2020).

Neurotransmitters or other secretory molecules leave the ER packed in COPII-coated vesicles to be further processed in the Golgi apparatus before being secreted into the synaptic cleft. After an action potential, neurotransmitter and receptors are recycled by the cell. They are protected from lysosomal degradation by COPI-coated vesicles of the retrograde trafficking and redirected to the ER where they are recycled for the next release (Lu and Hong, 2014).

Deficiencies in axonal transport through defects in the cytoskeletal organization, the impairment of motor protein attachment to the microtubules or the destabilization of motor-cargo binding but also aberrant transmembranal anterograde and retrograde transport between ER and Golgi apparatus can induce synaptic failure and ultimately lead to neurodegeneration, as shown in AD, PD, ALS, and HD (Perlson et al., 2010; Guo et al., 2020).

### Hallmark 5: Immune System

A fundamental feature in NDD is the dysregulation of the innate *immune response* in the central nervous system by chronic inflammation. The innate immune response of the brain represents not only the first line of defense against invading microorganisms (*defense response to other organisms*) but also responds to endogenous stress like dying or damaged cells after cellular stress. It consists primarily of microglial cells and astrocytes. Microglia are resident tissue macrophages and the principal mediators of *inflammation* (Frank-Cannon et al., 2009), whereas astrocytes mainly supply neurons with nutrients and energy and form the blood–brain barrier (Blackburn et al., 2009). Both microglia and astrocytes express on their cell-surface pattern-recognition receptors that detect pathogen-associated molecular patterns like residues from bacteria, viruses, fungi but also danger-associated molecular patterns, which can be abnormal endogenous proteins, iron overload, antibodies, *cytokines*, and chemokines, including toll-like receptors (Akira et al., 2006; Medzhitov, 2007). Also, complement factors bind to these receptors, initiating the *complement and coagulation cascade*, which enhances the immune response of microglia and astrocytes.

Pathogen- and danger-associated molecular patterns induce neuroinflammation by activating the transcription factor NFkB in microglial cells. NFkB induces the synthesis of proinflammatory cytokines like Interleukin−1ß (IL-1β), IL-6, IL-12, interferon gamma (IFN-γ), chemokines, including the C-C motif chemokine ligand 2 and tumor necrosis factor alpha (TNF-α), but also prostaglandins (Schaefer, 2014; Guzman-Martinez et al., 2019) to promote efficient clearance of cell debris and plaques. Also, these factors, being released into the cell environment, activate the astrocytes to both increase the permeability of the blood–brain barrier for easier recruitment of B- and T-adaptive immune cells (*leukocyte activation*) in the brain parenchyma and to release intracellular signals like neurotrophic and growth factors and cytokines, promoting neuronal survival, neurite growth, and neurogenesis (Jha et al., 2019). At the same time, microglial cells synthesize and release short-lived cytotoxic factors, such as superoxide radicals, nitric oxide, and reactive oxygen species that help remove pathological agents (Harry and Kraft, 2008; Jurga et al., 2020).

Although an acute insult may trigger oxidative and nitrosative stress and a slightly more permeable blood-brain barrier, this reaction is typically short-lived and unlikely to be detrimental to long-term neuronal survival (Kurutas, 2015). But disease-induced protein aggregations like oligomers of tau and Aβ, aging, and other risk factors can lead to chronic neuroinflammation with a long-standing and, often, self-perpetuating neuroinflammatory response that persists long after the initial injury or insult. Permanently activated microglia may not be able to remove amyloid-beta deposits, which, in turn, contributes to further plaque accumulation as opposed to clearance (Frank-Cannon et al., 2009). Also, the permanently increased oxidative and nitrosative stress leads to damaged neurons, synaptic dysfunction, loss of synapses, and neuronal death (Schain and Kreisl, 2017; Shabab and Zorofchian, 2017; Kinney et al., 2018), aggravated by the fact that astrocytes lose their neurotrophic function during the progressive loss of neurons. This process called astrogliosis affects considerably the neurons and their integrity (Sofroniew and Vinters, 2010; Sofroniew, 2015). *C*hronic inflammation can also trigger signaling pathways that activate brain tau hyperphosphorylation in residues that are not modified under normal physiological conditions, again leading to degenerated neurons and, finally, the now permeable blood-brain barrier allows much more pathogens to enter the brain than the innate immune response can cope with (Gendelman, 2002).

### Hallmark 6: Metabolic Processes

We associated the highest number of low-level terms across all six hallmarks to the metabolism hallmark as its pathways and biological processes are involved in all basic physiological processes, and the homeostasis of the compounds and their interactions is vital for the well-functioning of the organism. As neurons but also astrocytes, oligodendrocytes, and microglia are highly dependent on a continuous energy supply. One of the most significant hallmarks for NDD is the dysfunction of mitochondria. Mitochondrial metabolism, comprising the oxidation of pyruvate and fatty acids, the citric acid cycle, the oxidative phosphorylation within the respiratory chain, and the formation of antioxidants, was assigned to the hallmark metabolism, which also contains metabolic processes in the cytoplasm like fat metabolism, protein, carbohydrate, and nucleic acid metabolism, but also homeostasis of the cell. Lipid molecules are key components of the brain's complex structure and function, with lipids comprising around 50% of the brain's dry weight (O'brien et al., 1964). Many neurological diseases are caused by mutations in genes that are involved in lipid metabolism (Mesa-Herrera et al., 2019; Petit et al., 2020). As associated low-level terms, we included *lipid-, cholesterol-, sphingolipid* metabolism, *lipoproteins, carboxylic acid*, and *fatty acid oxidation*. Lipids function as key regulators of neurotransmission; cholesterol is a membrane stabilizer and organizer during synaptic vesicle exo- and endocytosis; sphingolipids and their metabolites are regulators of the fluidity of cell membranes and second messengers in signal transduction (Puchkov and Haucke, 2013; Barber and Raben, 2019). Being hydrophobic, lipids and cholesterol must be transported through the blood by *lipoproteins*. The term carboxylic acid was mostly connected to biological processes concerning the citric acid cycle and was thus assigned to mitochondrial metabolism, like fatty acids, which are degraded in the mitochondria in an energy-generating process called ß-oxidation (Petit et al., 2020).

Main low-level terms of protein metabolism were branched amino acid degradation and *proteoglycans*. Valine, isoleucine, and leucine are essential branched-chain amino acids (BCAAs). Their catabolism starts in muscle mitochondria and yields *NADH* and FADH2, which can be utilized for ATP generation. In the brain, BCAAs contribute to the synthesis of excitatory glutamate and inhibitory gamma-aminobutyric acid (GABA) and, at the same time, can reduce the production of serotonin and the catecholamines because they compete for transport across the blood-brain barrier with the aromatic amino acids tryptophan, tyrosine, and phenylalanine, which are the precursors of these neurotransmitters (Fernstrom, 2005). AD metabolomics studies demonstrate that altered *branched-chain amino acids* metabolism accompanies Alzheimer's disease development. Lower plasma valine levels are correlated with accelerated cognitive decline in AD and the severity of motor dysfunction in HD (Polis and Samson, 2020; Xu et al., 2020). No clear correlation between the levels of BCAA and ALS or PD could be found (Bjornevik et al., 2019; Xu et al., 2020).

Carbohydrates are the main source for energy production in the brain and are broken down by glycolysis, the tricarboxylic acid cycle, and oxidative phosphorylation (Attwell and Laughlin, 2001). The main causes for defective glucose usage in NDD are deleterious mitochondria and a decreased secretion of insulin, coupled with resistance to its actions, problems that link NDD to type II diabetes (Ristow, 2004; Haan, 2006; Seneff et al., 2011; Schaeffer et al., 2021).

During nutritional uptake, the insulin pathway secures the release of the hormone insulin into the blood, which then binds to insulin receptors on the cell surface and thus regulates the uptake of glucose into the cells. But insulin also controls divergent signaling pathways like the Act-pathway or the MAP-kinase pathway, which are involved in cell growth and differentiation (Saltiel and Pessin, 2002). Studies with mouse models have shown that insulin is also involved in the excitability of (hippocampal) neurons (Dai et al., 2014). AD neurons show a reduced action potential due to soluble Aß, an effect that is aggravated by missing insulin and that can be partially reversed by the anti-cancer agent bexarotene (Cramer et al., 2012; Dai et al., 2014).

Another important task of carbohydrates is the glycosylation of proteins and lipids in the ER. In the brain, glycosylated proteins participate in a myriad of processes, from electrical gradients to neurotransmission (Conroy et al., 2021), and glycolipids are crucial for the interaction of cells and for signal transduction (Reily et al., 2019). Thus, an intact *N*-glycosylation and *O*-glycosylation of lipids and proteins are important for the well functioning of the brain, and disrupted *glycosylation* can lead to NDD (Moll et al., 2020), e.g., *O*-GlcNAcylation of CNS proteins important for axonal and synaptic function is significantly reduced in AD, ALS, HD, and PD patients' tissue (Liu et al., 2004; Lüdemann et al., 2005; Kumar et al., 2014).

*Nucleotides* are not only the building blocks of DNA and RNA but also are involved in cellular metabolism, with, especially, ATP and NADH as energy carriers and cAMP, a second messenger molecule, as a translator of signals from outside to cellular processes. Thus, these molecules are involved in all basic physiological processes critical to the proper function of the organism (Huang et al., 2021). Nucleotides are either built *de novo* or, as this process takes much energy, recovered during the degradation of DNA and RNA in a nucleotide salvage pathway. A disbalance in the synthesis pathways again disturbs energy homeostasis and many cellular processes like proliferation, differentiation, migration, and apoptosis. The *respiratory chain complex* in the mitochondria not only efficiently produces energy but also reactive oxygen species that can harm the mitochondrial DNA. Antioxidants like glutathione protect mitochondria and cells against damage, but, if this *homeostasis* is disturbed, oxidative stress induces the destruction of mitochondria and apoptosis of neuronal and other brain cells (Kausar et al., 2018).

## Materials and Methods

To make all steps from data acquisition *via* data management to data analysis comprehensible, we will outline these steps following that order. The data analysis is composed of several steps, the analysis of the intersections of the individual omics-layers per NDD, the analysis of the conformity of the mean regulatory direction across omics-layers per NDD, as well as the core of the data analysis: the generation and evaluation of the protein-protein interaction networks, and the generation and evaluation of the communities.

### Data Acquisition

In this meta-study, we used datasets of four different omics-layers: Genomics (SNP data), Transcriptomics, Proteomics, and Methylomics. In the following, we describe where these data were acquired from.

The SNP data are based on the genome-wide association studies (GWAS) Catalog (Buniello et al., 2019), which has been updated on November 18, 2021. The experimental factor ontology (EFO) numbers for the exact search pattern were EFO_0000249 (Alzheimer's disease), EFO_0002508 (Parkinson's disease), Orphanet_399 (Huntington's disease), and EFO_0000253 (Amyotrophic Lateral Sclerosis). For all NDD but HD, a child term was given, which was also included in the data acquisition step. In total, 153 studies with genomic data were used for the analyses (Maraganore et al., 2005; Fung et al., 2006; Coon et al., 2007; Reiman et al., 2007; Schymick et al., 2007; van Es et al., 2007; Van Es et al., 2008, 2009; Abraham et al., 2008; Bertram et al., 2008; Cronin et al., 2008, 2009; Li et al., 2008, 2021; Webster et al., 2008; Beecham et al., 2009, 2015; Carrasquillo et al., 2009; Harold et al., 2009; Lambert et al., 2009, 2013a,b; Landers et al., 2009; Latourelle et al., 2009; Pankratz et al., 2009, 2012; Satake et al., 2009; Simón-Sánchez et al., 2009; Edwards et al., 2010; Feulner et al., 2010; Hamza et al., 2010; Heinzen et al., 2010; Laaksovirta et al., 2010; Naj et al., 2010, 2011; Seshadri et al., 2010; Shatunov et al., 2010; Stein et al., 2010; Antúnez et al., 2011; Do et al., 2011; Furney et al., 2011; Hollingworth et al., 2011, 2012; Hu et al., 2011, 2016; Kim et al., 2011; Kramer et al., 2011; Liu et al., 2011, 2021; Logue et al., 2011; Nalls et al., 2011, 2014, 2019; Saad et al., 2011; Spencer et al., 2011; Wijsman et al., 2011; Chung et al., 2012, 2018; Cummings et al., 2012; Kamboh et al., 2012a,b; Kwee et al., 2012; Lill et al., 2012; Meda et al., 2012; Cruchaga et al., 2013; Davis et al., 2013; Deng et al., 2013; Jonsson et al., 2013; Langefeld, 2013; Martinelli-Boneschi et al., 2013; Miyashita et al., 2013; Reitz et al., 2013; Diekstra et al., 2014; Fogh et al., 2014, 2016; Goris et al., 2014; Hill-Burns et al., 2014, 2016; Kauwe et al., 2014; Nelson et al., 2014; Pérez-Palma et al., 2014; Ramanan et al., 2014; Ramirez et al., 2014; Sherva et al., 2014, 2020; Vacic et al., 2014; Xie et al., 2014; Hirano et al., 2015; McLaughlin et al., 2015; Tosto et al., 2015; Wang et al., 2015, 2021; Biernacka et al., 2016; Chen et al., 2016; Deming et al., 2016; Herold et al., 2016; Jun et al., 2016, 2017; Pickrell et al., 2016; Schott et al., 2016; Traylor et al., 2016; Van Rheenen et al., 2016; Watanabe et al., 2016; Benyamin et al., 2017; Chang et al., 2017; Foo et al., 2017, 2020; Lee et al., 2017; Mez et al., 2017; Moss et al., 2017; Siitonen et al., 2017; Sims et al., 2017; Chao et al., 2018; Gusareva et al., 2018; Marioni et al., 2018; Miron et al., 2018; Mukherjee et al., 2018; Nicolas et al., 2018; Pottier et al., 2018; Wallen et al., 2018; Yashin et al., 2018; Bandres-Ciga et al., 2019; Blauwendraat et al., 2019, 2020; Dekker et al., 2019; Jansen et al., 2019; Kunkle et al., 2019, 2021; Lo et al., 2019; Moreno-Grau et al., 2019; Nazarian et al., 2019a,b; Wei et al., 2019; Zhu et al., 2019; Cha et al., 2020; Hong et al., 2020; Nakamura et al., 2020; Ryu et al., 2020; Alfradique-Dunham et al., 2021; Bone et al., 2021; DeMichele-Sweet et al., 2021; de Rojas et al., 2021; Kang et al., 2021; Loesch et al., 2021; Park et al., 2021; Reddy et al., 2021; Rodrigo and Nyholt, 2021; Sakaue et al., 2021; Schwartzentruber et al., 2021; Shigemizu et al., 2021; Smeland et al., 2021; Tan et al., 2021; Wightman et al., 2021).

For the transcriptomic and proteomic layer, we used the studies that we have identified previously (Ruffini et al., 2020), using the Gene Expression Omnibus (GEO) (Barrett et al., 2013), the Expression Atlas (Papatheodorou et al., 2018) databases, and doing literature research (Ruffini et al., 2020). For the transcriptomic data, the keywords used in the GEO database were <name of disease> AND (“microarray” OR “RNAseq”) AND “human,” The Expression Atlas was used in Release 35 (May 2020, https://www.ebi.ac.uk/gxa/home) and scanned for Alzheimer's, Parkinson's, Huntington, and Amyotrophic Lateral Sclerosis, using the filter “Homo sapiens” in the section “Differential Experiments.”

For the proteomic data, literature search was conducted in PubMed and Google Scholar, with the keywords: (“neurodegenerative diseases” OR “Alzheimer's^*^ disease” OR “Parkinson's^*^ disease” OR “Huntington^*^ disease” OR “Amyotrophic Lateral Sclerosis”) AND (proteomics OR “quantitative proteomics” OR “differentially expressed proteins” OR biomarkers) AND human NOT mice for publications from 2010 to 2020.

Of the included experiments, 63% of the transcriptomic experiments were performed with brain material, while 67% of the proteomic experiments were conducted with brain material. Among the non-control patients, 84% were classified as having a moderate or severe condition in the transcriptome experiments and 90% in the proteome experiments. In total, transcriptomic data of 39 studies (Blalock et al., 2004, 2011; Zhang et al., 2005; Dunckley et al., 2006; Lesnick et al., 2007; Liang et al., 2007; Scherzer et al., 2007; Simunovic et al., 2009; Cox et al., 2010; Elstner et al., 2011; Dumitriu et al., 2012, 2016; Feyeux et al., 2012; Berchtold et al., 2013; Hokama et al., 2014; Riley et al., 2014; Calligaris et al., 2015; Dijkstra et al., 2015; Labadorf et al., 2015; Magistri et al., 2015; Prudencio et al., 2015; Raman et al., 2015; Ring et al., 2015; Kapeli et al., 2016; Lin et al., 2016; Scheckel et al., 2016; Lim et al., 2017a,b; Gagliardi et al., 2018; Mehta et al., 2018; Stopa et al., 2018; Mathys et al., 2019; Meyer et al., 2019; Otake et al., 2019; Swindell et al., 2019; Switońska et al., 2019; Al-Dalahmah et al., 2020; Dols-Icardo et al., 2020; Higginbotham et al., 2020) and proteomic data of 22 studies were acquired (Fang et al., 2009; van Dijk et al., 2012; Varghese et al., 2013; McQuade et al., 2014; Riley et al., 2014; Collins et al., 2015; Dumitriu et al., 2016; Hondius et al., 2016; Ratovitski et al., 2016; Lachén-Montes et al., 2017, 2019; Seyfried et al., 2017; Umoh et al., 2018; Zhang et al., 2018; Higginbotham et al., 2019, 2020; Iridoy et al., 2019; Bader et al., 2020; Johnson et al., 2020; Oeckl et al., 2020; Rotunno et al., 2020; Wingo et al., 2020) (see also Table 1). A table giving the number of samples per condition, the severity state of the included samples, and the used technology is given in Supplementary Table 4.

**Table 1:** A summary of the number of used studies per disease and omics-layer.

**NDD**	**Genome**	**Transcriptome**	**Proteome**	**Methylome**	**Studies**
AD	79	11	9	10	109
PD	46	11	7	7	71
HD	2	10	5	2	19
ALS	26	8	3	2	39
∑	153	40 (39)	24 (22)	21 (20)	238 (234)

*In total, data of four studies were used for more than one omics-layer, resulting in 234 different studies as a basis for this meta-studies data analysis. In brackets, the actual summed number of unique studies used is shown, as some studies contained information of two NDDs*.

For the methylomic data, we used all data from the PDMethDB (Wang C. et al., 2020) (last accessed December 13, 2021), the EWAS Atlas (Li M. et al., 2019) (accessed 01.02.21) for the four respective diseases, and other data we found through literature research using PubMed and Google Scholar. Queries for “epigenome-wide” OR “epigenome wide” OR “EWAS” OR “genome-wide” AND “methylation” OR “genome wide” AND “methylation,” as well as “metastudy” AND “neurodegenerative disease” AND “epigenetic,” were used to find studies published after 2010. Combined, the databases and literature research resulted in the inclusion of data of 20 studies (Bakulski et al., 2012; Kaut et al., 2012; Masliah et al., 2013; de Jager et al., 2014; Lunnon et al., 2014; Sanchez-Mut et al., 2016; Watson et al., 2016; Zhang et al., 2016; Young et al., 2017, 2019; Gasparoni et al., 2018; Smith et al., 2018, 2019; Zadel et al., 2018; Altuna et al., 2019; Li P. et al., 2019; Tarr et al., 2019; Go et al., 2020; Lu et al., 2020; Marshall et al., 2020), one of which contained data for both AD and PD. As the higher availability of methylome data for Alzheimer's and Parkinson's diseases made it possible, we chose to include exclusively brain tissue-derived data for those two diseases. However, as the data availability for HD and ALS were much poorer, only blood tissue data were included for those NDDs. In total, we gathered data from 234 different studies for the four respective diseases and omics-layers (see Table 1).

### Data Management

The raw gene lists obtained from the transcriptomic, proteomic, and methylomic data were filtered for a false discovery ratio (FDR) < = 0.05, the proteomic data were mapped to gene names, and all the obtained gene names (from genomic, transcriptomic, proteomic, and methylomic data) were then mapped to the list of current protein-coding gene symbols (22 March 2021) from the HUGO Gene Nomenclature Committee (Tweedie et al., 2021) by mapping all alias names to the original gene symbol. Those names that were not found as any gene symbol or alias were discarded. The following data management step consisted of removing those genes that appeared as significantly altered only once in our transcriptomic, proteomic, and methylomic data. However, since the database for our methylomic and proteomic data in ALS and for the proteomic data in HD was very sparse, we decided to retain all significant genes for these gene lists to interpret a potentially noisy signal instead of hardly any signal at all.

### Mean Direction of Regulation

The transcriptomic, proteomic, and methylomic data also contained information about the regulation of these significant genes/proteins. This information was contained as G-fold change (FC), log2 FC or other in transcriptomic and proteomic experiments, and as beta values in some methylomic experiments. To make this information from the different data sources as comparable as possible, we derived the mean direction of regulation (MeanRegDir) for all significantly conspicuous genes in each of the three omics levels, which equals to 1 if a gene/protein was overexpressed/hypomethylated in the disease group in every instance in which it occurred. Conversely, MeanRegDir indicates a value of −1 if a gene in the disease group was downregulated/hypomethylated in all experiments in which it appeared as significantly striking. If the signal is ambiguous, MeanRegDir will give a value between −1 and 1, as indicated in Equation (1).

Equation 1: Calculation of mean regulation of direction


$$\begin{array}{l} MeanRegDir\left(gene\right)=\frac{1}{n}\overset{n}{\underset{i=1}{\sum }}\left(sig\left({gene_{i}}_{foldChange}\right)\right)\; \end{array}$$

with

*n* = number of appearances for gene with FDR ≤ 0.05

$sig\left(x\right)=\;\{\begin{array}{c} 1,\;if\;x>0\\ -1,\;if\;x<0 \end{array}$

*gen*_*e*_*i*_*foldChange*_ = *fold change of gene*_*i*_

### Method 1: Omics-Layer Intersections per Disease

By intersecting the four analyzed omics-layers per disease, we were able to test whether the number of shared genes between each pair of omics-layers per disease was significantly increased. We used a hypergeometric test for the overlapping sets, with the total amount of 19,207 gene symbols of protein-coding genes (HUGO Gene Nomenclature Committee, 22 March 2021) (Braschi et al., 2019) as the total population. To also have a measure for the effect size of the significantly high numbers of genes in the overlapping sets and thus being able to differentiate between the differently significantly found large gene sets, we calculated the effect size of the hypergeometric test results by setting the difference between observed and expected value in relation to the standard deviation of the hypergeometric distribution. This led to the following formula and the value *eff*_*size*_ that gives the number of standard deviations by which our expected value is beyond/below the expected value:

Equation 2: Effect size of the hypergeometric test results


$${\text{eff}}_{\text{size}}\text{=}\frac{{\text{overlap}}_{\text{obs}}{-\text{ overlap}}_{\exp}}{{\text{std}}_{\text{hypergeom}}}$$

with:


$$\begin{array}{l} std_{hypergeom}=\;\sqrt{n*\frac{K}{N}*\frac{N-K}{N}*\frac{N-n}{N-1}} \end{array}$$

with:


$$\begin{array}{l} overlap_{obs}=\;observed\;overlap\;between\;the\;two\;omics\;layers\\ overlap_{\exp}=\;expected\;overlap\;between\;the\;two\;omics\;layers\\ N=\;population\;size\;\left(whole\;gene\;background\right)\\ K=\;number\;of\;successes\;in\;the\;population\\ \left(number\;of\;genes\;in\;the\;first\;omics\;layer\right)\\ n=\;number\;of\;draws\;\\ \left(number\;of\;genes\;in\;the\;second\;omics\;layer\right)\\ k=\;number\;of\;observed\;successes\\ \left(number\;of\;overlapping\;genes\right) \end{array}$$

Intersections were performed and visualized using the R (version 4.0.2) (Urbanek et al., 2014) package *venn* (version 1.10) (Dusa, 2016).

### Method 2: Multi-Omics Conformity

#### Stacked Bar Plots

In order to visualize those genes, which are appearing as altered/differentially expressed in multiple omics-layers, stacked bar plots showing conformity in genomics, transcriptomics, proteomics, and methylomics data (Supplementary Presentation 2—StackedBarPlots) were created using the python package *matplotlib* (Hunter, 2007). As the overlap between genomics and the other three omics-layers was rather low, stacked bar plots were also created for those genes appearing only in transcriptomic, proteomic, and methylomic levels. Plots were only created if there was any overlap. To gain insights into the direction of regulation (up- or downregulation) for the overlapping genes, the stacked bars are also color-coded. The color represents the ratio of studies in which a gene was significantly up- or downregulated (see Equation 1 Calculation of mean regulation of direction). This data representation shows information about the certainty of the direction of differential regulation as well as coherency between transcriptomic, proteomic, and methylomic over- or underrepresentation or hyper-/hypomethylation, respectively.

#### Correlation Test

The *pearson* function that is available in the *scipy.stats* module (Virtanen et al., 2020) was used to compute the correlation and its two-sided *p*-value for the relation between transcriptomic and proteomic direction of regulation. The correlation analysis showed if there was a significant correlation between the direction of regulation in the pairwise intersection between the transcriptomic, proteomic, and methylomic levels. The value of the pairwise correlation and information about the *p*-value of the correlation test was calculated for each pairwise intersection of the transcriptomic, proteomic, and methylomic data layers for each disease. The results are shown as a heatmap in **Figure 2**.

### Method 3: Protein-Protein Interaction Networks

The significantly distinctive genes and proteins that emerged in our analysis represented a variety of processes connected to the respected NDD. Consequently, the functional analysis of all genes that emerged for an individual omics-layer for a particular NDD would lead to results showing a combination of different related processes and would, therefore, lower the statistical power of the hypergeometric test and interpretability of the results.

To better separate the distinct processes related to our gene sets, we constructed protein-protein interaction (PPI) graphs for each omics-layer of each NDD and isolated communities of high modularity within that graph with the louvain algorithm (Blondel et al., 2008). These communities of high modularity were then used for the functional analyses and showed clearly distinct processes for our different gene sets.

#### Network Construction

PPI networks were constructed to visualize and utilize protein-protein interaction data for each omics layer per NDD, which were subsequently used to identify communities of high modularity and associated hub genes within each network. The networks were constructed using the Python module *louvain* (2.6.3) (Hagberg et al., 2008). The PPI data were based on the *louvain* dataset for human PPI enrichment (species = 9,606, accessed on October 18, 2021) and were filtered for those PPI with a combined score > = 400 (default value). The combined score in string DB consists of a combination of the individual scores (annotated pathways, gene neighborhood, …), emerging from the underlying databases and is typically higher than the individual scores, as it expresses increased confidence if a connection between two genes is supported by different types of evidence (von Mering et al., 2005). This resulted in a graph that consisted of nodes representing the genes that are connected by weighted edges, representing the combined interaction score of the involved genes. These weights were later used in the hub gene identification to represent the importance of an edge or to calculate a distance between edges as *distance* = 1/*weight*, depending on each centrality algorithms input requirements.

Furthermore, the modularity of the networks was computed using the Python module *louvain*, with the *louvain.algorithms.community.quality.modularity* function with a resolution parameter set to 1. For this function, modularity is defined as described by Newman (2010) and shown below:

Equation 3: Modularity


$$\begin{array}{l} Q=\frac{1}{2m}\underset{ij}{\sum }\left(A_{ij}-\gamma \frac{k_{i}k_{j}}{2m}\right)\delta \left(c_{i},c_{j}\right) \end{array}$$

with

*m* = number of edge*s*
*A* = *adjacency matrix of Graph G*
*k*_*i*_ = degree of node *u*_*i*_
γ = *a resolution parameter* (*for this analysis, set to* 1)

$\delta \left(c_{i},\;c_{j}\right)=\;\{\begin{array}{c} 1,\;if\;u_{i}\;and\;u_{j}\;are\;in\;the\;same\;community\\ 0,\;otherwise \end{array}\;$

#### Community Creation

The community search was carried out using the Python module *community* (1.0.0b1) with the *community.best_partition* function, which computes a partition of the graph, maximizing the modularity using the *louvain* heuristics (Blondel et al., 2008). As the *louvain* algorithm's result varies with the order in which the input is given, we applicated the louvain algorithm 100 times with permutated input of the whole network for each omics-layer in each disease and calculated the whole graph's modularity, picking the best result in terms of network modularity as our final community output.

#### Functional Analysis and Hallmark Definition

For each of the resulting communities, we performed the functional annotation using the stringDB API (stringDB Version 11.5) and further focused on all communities with at least one biological process with an FDR < = 0.05, as we considered those communities as most helpful for creating a meaningful redefinition of the neurodegenerative hallmarks. We also visualized these communities of the networks in Arcplots and Matrixplots using the Python module *nxviz* (0.7.2).

Derived from the functional analysis results, we defined six hallmarks that resemble the most meaningful categorization of our results, given prior knowledge and the most recurring processes throughout our multi-omics analysis of the four NDDs. When interpreting the functional analysis results and defining not only the six hallmarks but also low-level terms for each hallmark, we especially focused on the GO terms, pathways, and stringDB local network clusters that were associated with our communities. The stringDB local network clusters are precomputed protein clusters from stringDB that are derived from hierarchical clustering of the whole stringDB PPI network and further reduced by excluding all child clusters that are too redundant and only vary from the parent cluster by less than five proteins. These clusters' names are then derived from the proteins' annotation given in databases from *Gene Ontology* (GO) (Carbon et al., 2021), *KEGG* (Kanehisa et al., 2021), *Reactome* (Fabregat et al., 2016), *UniProt* (Bateman et al., 2021), *Pfam* (Mistry et al., 2021), *SMART* (Letunic et al., 2021), and *InterPro* (Blum et al., 2021).

The actual analysis of the associations between our communities and the low-level terms was performed by comparing the found number of associations with the expected number of associations per NDD and omics-layer based on the overall distribution. Additionally, we performed an X^2^-Test across all NDDs and, also, across all omics-layers to see if there was a statistically significant difference between the expected and observed numbers of associations across the four NDDs or across the four omics-layers for all six defined hallmarks.

#### Hub Gene Identification

To find out if, for some of our extracted communities, there were key players clearly playing the most prominent role in their respective communities, we aimed to identify the hub genes in our communities, meaning the most central node in our community networks. In network theory, there are different concepts for determining the centrality of a node, meaning the node's dominance within the network. Three widely used centrality measures were originally described by Freeman in the late '70s, namely, degree centrality, betweenness centrality, and closeness centrality (Freeman, 1977, 1978; Freeman et al., 1979), based on the number of direct links between a node and other nodes, the mediation role of a node in a network and on the sum of the shortest paths to all other nodes in the network, respectively. For closeness centrality, the Wassermann- and Faust-improved formula was used (Wasserman and Faust, 1994). These three centrality measures were computed with the Python module *networkx* and were based on the following formulas:

Equation 4: Degree centrality of node *u*_*i*_


$$\begin{array}{l} C_{d}\left(u_{i}\right)=\frac{1}{N}*\overset{N}{\underset{j=1}{\sum }}X_{i,j} \end{array}$$

with:

*i* ≠ *j*;
*N* = *number of nodes in the graph*;

$X_{i,j}=\;\{\begin{array}{c} 1,\;if\;u_{i}\;and\;u_{j}\;are\;connected\;by\;an\;edge\;\\ 0,\;otherwise \end{array}\;$

Equation 5: Betweenness centrality of node *u*_*i*_


$$\begin{array}{l} C_{b}\left(u_{i}\right)=\frac{n-1}{\sum_{j=1}^{n-1}d(u_{i},\;u_{j})\;}*\frac{n-1}{N-1}\\ \;C_{b}\left(u_{i}\right)=\sum_{s,t\;\in V}\frac{\sigma (s,t|u_{i})}{\sigma (s,t)} \end{array}$$

with:

*s, t* ≠ *u*_*i*_; *s* ≠ *t*;
*V* = *set of all nodes in the graph*;
σ(*s, t*) = *the number of shortest s, t paths*
σ(*s, t*|*u*_*i*_) = *the number of shortest s, t paths going through u*_*i*_

Equation 6: Closeness centrality of node *u*_*i*_ (Wasserman- and Faust-improved formula)


$$\begin{array}{l} C_{c}(u_{i})=\frac{n-1}{\sum_{j=1}^{n-1}d(u_{i},\;u_{j})\;}*\frac{n-1}{N-1} \end{array}$$

with:

*n* = *number of reachable nodes from u*_*i*_;
*N* = *number of nodes in the graph*;

$d\left(u_{i},u_{j}\right)=shortest\;path^{{}^{\prime }}s\;distance\;between\;node\;u_{i}and\;node\;u_{j}\;$

In addition to these three centrality measures originally proposed by Freeman, we also computed the eigenvector centrality of each node (Newman, 2010; Bonacich, 2015), which gives a centrality score to each node that is proportional to the sum of the centrality scores of each node's neighbors.

Equation 7: Eigenvector centrality of node u_i_

*C*_*e*_(*u*_*i*_) is the i-th element of the vector x defined by:


$$\begin{array}{l} Ax=\;\lambda x \end{array}$$

*with A* = *adjacency matrix of the graph with eigenvalue λ*.

The current literature on finding hub genes in biological networks differs but often uses the node degree (Li et al., 2018; Zhou et al., 2018) or weighted gene coexpression network analysis (Zhang and Horvath, 2005) as a basis for identification of hub genes. Here, we combined the four mentioned centrality measurements that give information for each node *u*_*i*_ about:

- The number of other nodes in the network connected to *u*_*i*_
- The number of shortest paths between two other nodes in which *u*_*i*_ is involved
- The accumulated distance to all other nodes in the network from *u*_*i*_
- The importance of nodes connected to *u*_*i*_

We considered hub genes in our communities as those that are among the most important nodes in several of these centrality measures. Accordingly, we defined hub genes as being among the top three nodes in at least three of the four centrality measures mentioned. Genes that appeared among the three most central nodes in the network on all four calculated measures of centrality, or that were the single most central nodes according to at least three measures of centrality, were given special consideration for subsequent interpretation of hub genes in our community networks.

## Results

### Omics-Layer Intersections per Disease

**Figure 1:**
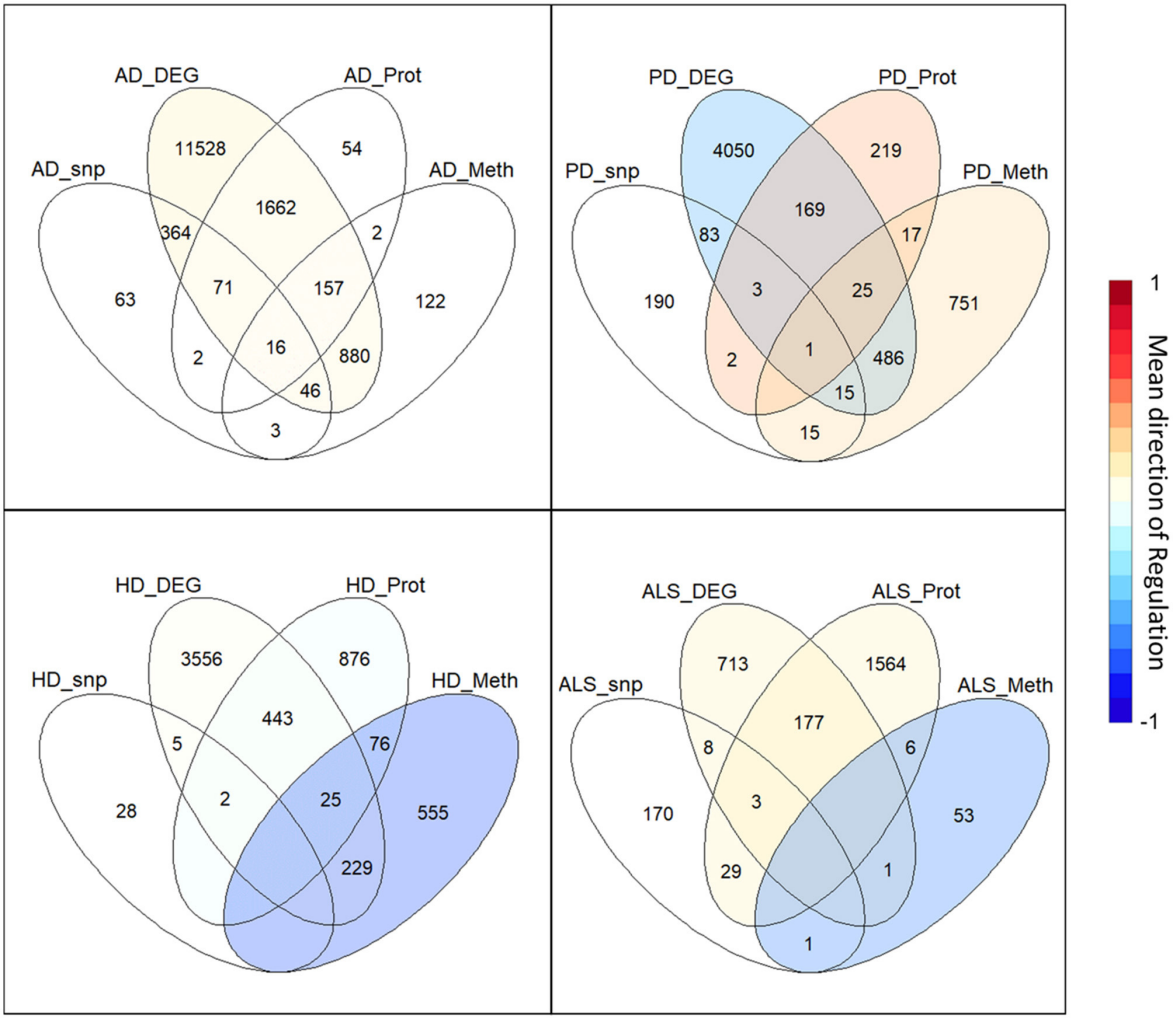
Omics-layer intersections per disease: The Venn diagrams for the four NDDs, AD, PD, HD, and ALS are shown with the numbers of shared genes in the intersections of the specific omics-layers. Additionally, the transcriptomic, proteomic, and methylomic layers of each NDD are colored to give each layer's mean direction of regulation. This color represents the color of only the respective layer, not of the individual intersections, as those have a specific regulation for each omics-layer.

The intersection of the four analyzed omics-layers per disease showed significantly high numbers of shared genes between the transcriptomic and proteomic levels for all four analyzed NDDs (Figure 1, Table 2). All other pairwise intersections show a significantly high number of shared genes in two to three of the analyzed NDDs, while AD shows significantly high numbers of shared genes for all pairwise intersections of omics-layers. AD also showed the highest number of genes in total across all omics-layers with 14,970 genes. PD followed with 6,026, and then HD with 5,795 and ALS with 2,725 genes (Figure 1). The mean direction of regulation was close to 0 for all omics-layers in AD (0.13, −0.1, −0.06 for transcriptomic, proteomic, and methylomic data, respectively); in PD, the mean direction of regulation was −0.59 for the transcriptomic level, 0.42 for the proteomic level, and 0.33 for the methylomic level. For HD, the methylomic layer showed a mean direction of regulation of −0.8, while the other omics-layers were closer to 0 (0.04 transcriptomic, −0.18 proteomic). In ALS, the transcriptomic and proteomic layers showed a positive mean direction of regulation (0.12 and 0.16), while the methylomic layer showed a strong negative mean direction of regulation (−0.75). For the genomic level, there are no mean directions of regulation as these data show an association between the presence of specific SNPs and the respective NDDs. The calculated effect sizes for the hypergeometric test results show the highest values in the transcriptomic-proteomic intersection for all NDDs, with differences between expected and observed numbers of genes of more than 10 standard deviations (stds) except for PD (second highest value with 9.9 stds). In PD, the highest effect size was achieved in the transcriptomic-methylomic intersection with 13 stds difference. All other comparisons showed much weaker effect sizes, with values below 5 stds.

**Table 2:** Omics-layers intersections per disease: the number of resulting genes in each pairwise intersection of omics-layers per disease was tested for enrichment in a hypergeometric test.

	**DEG-Prot**		**DEG-SNP**		**DEG-Meth**		**Prot-SNP**		**Prot-Meth**		**Meth-SNP**	
**NDD**	***p***	**Eff. size**	***p***	**Eff.size**	***p***	**Eff.size**	***p***	**Eff.size**	***p***	**Eff.size**	***p***	**Eff.size**
AD	**0**	**22.5**	**1.6E-12**	**6.4**	**0.00**	**11.1**	**1.3E-05**	**4.4**	**1.5E-06**	**4.8**	**1.4E-06**	**5.1**
PD	**0**	**9.9**	**8.2E-04**	**3.2**	**0.00**	**13.0**	0.56	−0.4	**6E-03**	**2.5**	**0.01**	**2.2**
ALS	**0**	**11.3**	0.29	0.4	0.79	−1.1	**2E-03**	**3.0**	0.33	0.2	0.14	0.4
HD	**0**	**10.3**	0.53	−0.3	**1.4E-06**	**4.8**	0.49	−0.4	**4.6E-06**	4.7	0.81	−1.3

*The results show that the number of significantly striking genes that emerged in transcriptomic and proteomic data was significantly high in all four analyzed diseases. The number of genes in the other pairwise intersections was only significantly high in two to three of the analyzed diseases. AD, the NDD with the largest database in our study, showed significantly high numbers of genes in all pairwise intersections. Additionally, the effect sizes of these hypergeometric test results are depicted as explained in Equation (2). Especially, the intersections between transcriptomic and proteomic layers show large effect sizes in all groups. Cells associated with significant overlaps are depicted in bold letters. SNP, genomic level; DEG, transcriptomic level; Prot, proteomic level; Meth, methylomic level*.

### Multi-Omics Conformity

**Figure 2:**
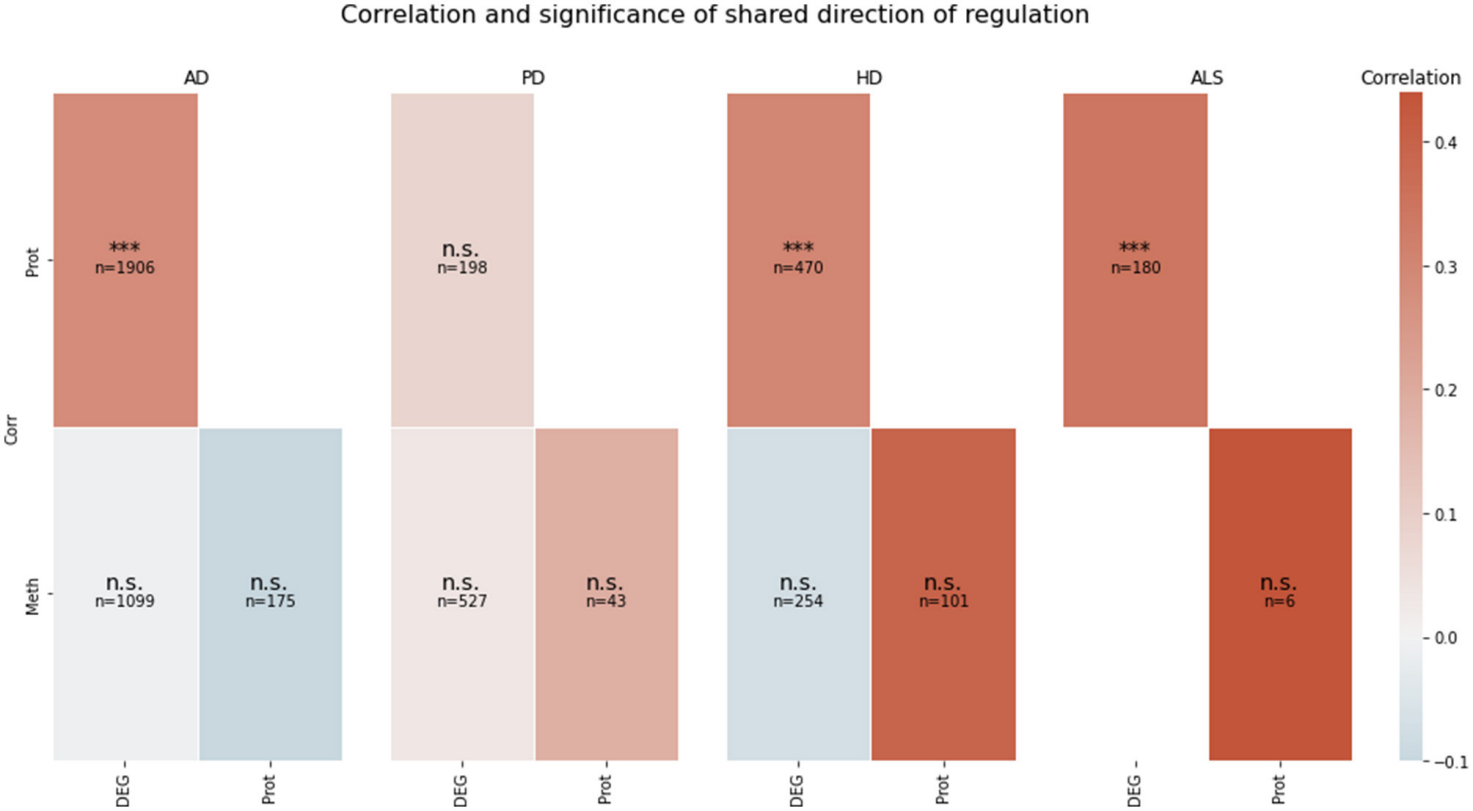
Pairwise correlation of the omics-layers per disease. For each of the four NDDs, a heatmap is showing the pairwise correlation value of the mean direction of regulation between each pair of omics-layers, which shows a direction of regulation (transcriptomics, proteomics, methylomics). Additionally, the significance of the correlation is given in each tile (n.s., not significant, ***p < 0.001). SNP, genomic level; DEG, transcriptomic level; Prot, proteomic level; Meth, methylomic level.

The correlation between the transcriptomic and proteomic mean direction of regulation of the genes in the transcriptomic and proteomic layers' intersection in AD, HD, and ALS was significantly high (all *p* < 10^−5^) (see Figure 2). The correlation for this layer intersection in PD was not significantly high. However, the correlation was slightly positive, even though the mean direction of regulation for the whole transcriptomic PD data was highly negative (−0.59), while it was highly positive (0.42) for the whole proteomic PD data. All other correlations of mean direction of regulation in the pairwise intersections between transcriptomic, proteomic, and methylomic layers per disease were neither significantly high nor low (*p* ≥ 0.05).

### Protein-Protein Interaction Networks

The network construction step led to a PPI network for each analyzed omics-layer in all four analyzed NDDs. However, the constructed communities of the networks based on the genomic layer in HD and ALS, as well as the methylomic layer in ALS, showed a little functional signal. Consequently, we focused on the remaining 13 PPI networks for the analysis.

**Figure 3:**
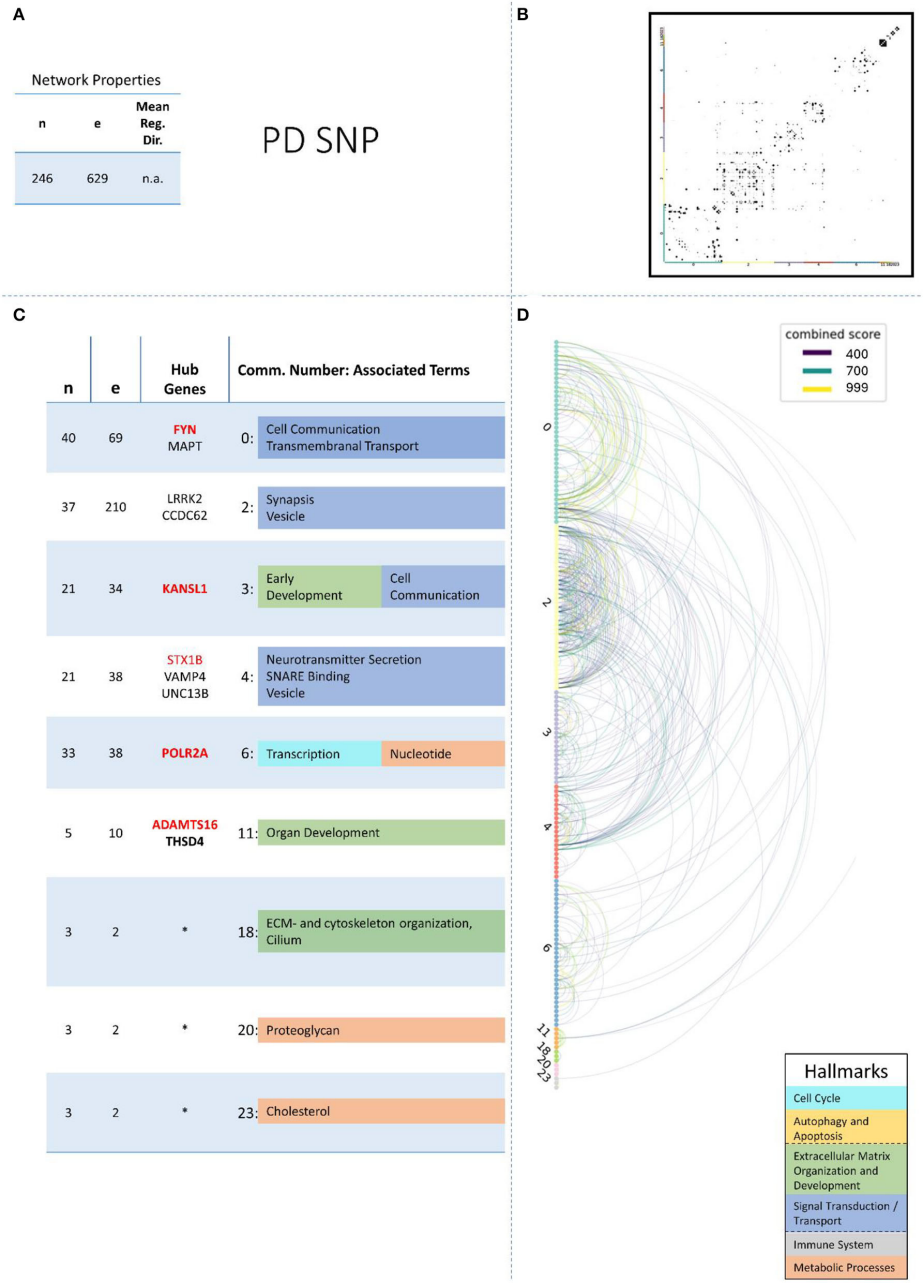
Summarizing figure of the network analysis results of the genomic layer of PD. The general properties, such as number of nodes (genes) and edges (a combined score > 400 between two nodes) of the PPI, network constructed with all PD genomic genes are given in Subfigure **(A)**. The individual connections with a combined score > = 400 between each two genes are also given in a matrix plot **(B)** and an Arcplot **(D)** that are ordered by community. The latter shows the confidence of the connection between two genes additionally by the color of the arc. The general properties of each individual community, as well as their hub genes and the categorization of their functional annotation, are given in the table in Subfigure **(C)**. The connected processes are colored by the associated hallmarks that are also displayed in the bottom right corner.

**Figure 4:**
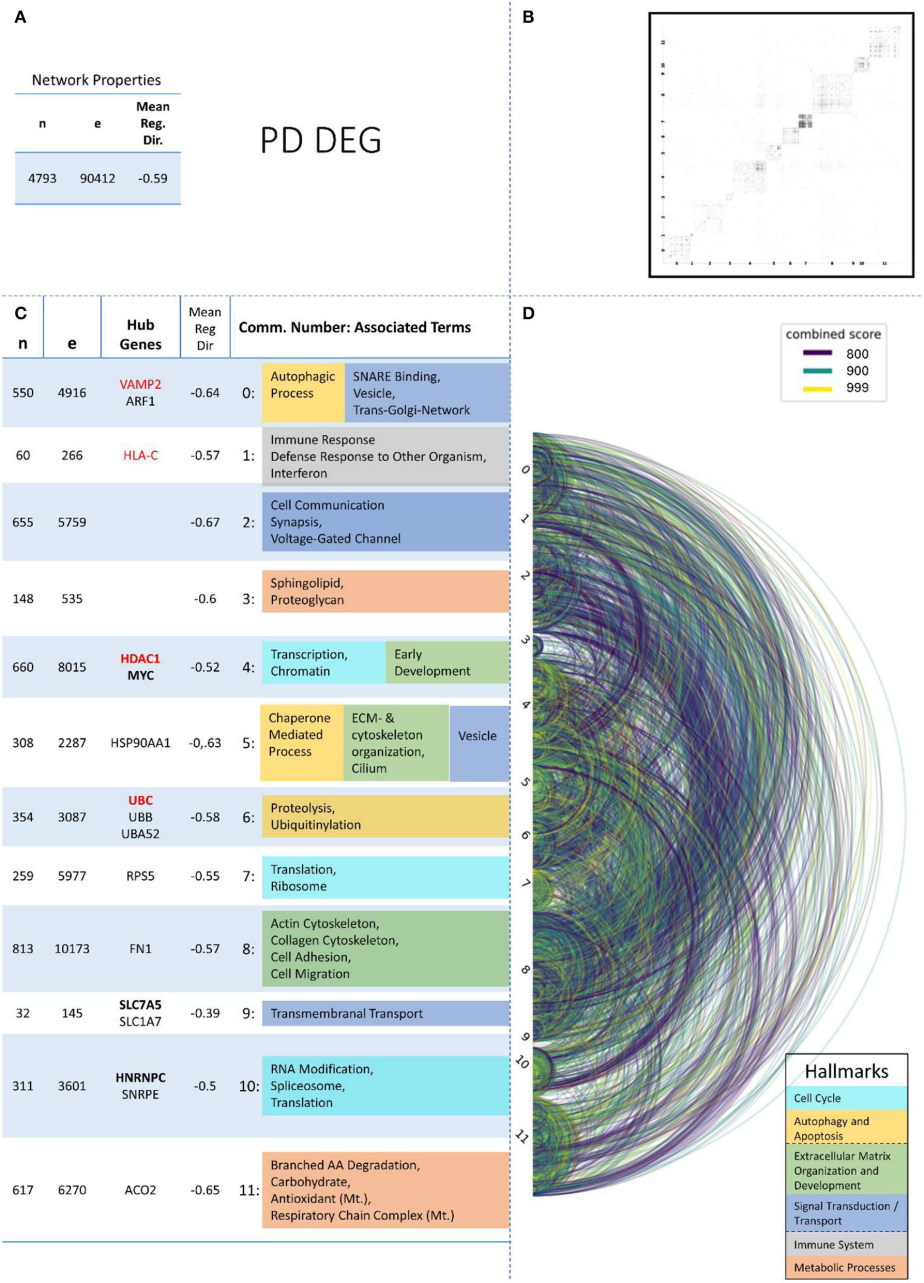
Summarizing figure of the network analysis results of the transcriptomic layer of PD. The general properties, such as number of nodes (genes) and edges (a combined score > 400 between two nodes) of the PPI network constructed with all PD transcriptomic genes, are given in Subfigure **(A)**. The individual connections with a combined score > = 800 between each two genes are also given in a matrix plot **(B)** and an Arcplot **(D)** that are ordered by community. The latter shows the confidence of the connection between two genes additionally by the color of the arc. The general properties of each individual community, as well as their hub genes and the categorization of their functional annotation, are given in the table in Subfigure **(C)**. The connected processes are colored by the associated hallmarks that are also displayed in the bottom right corner.

**Figure 5:**
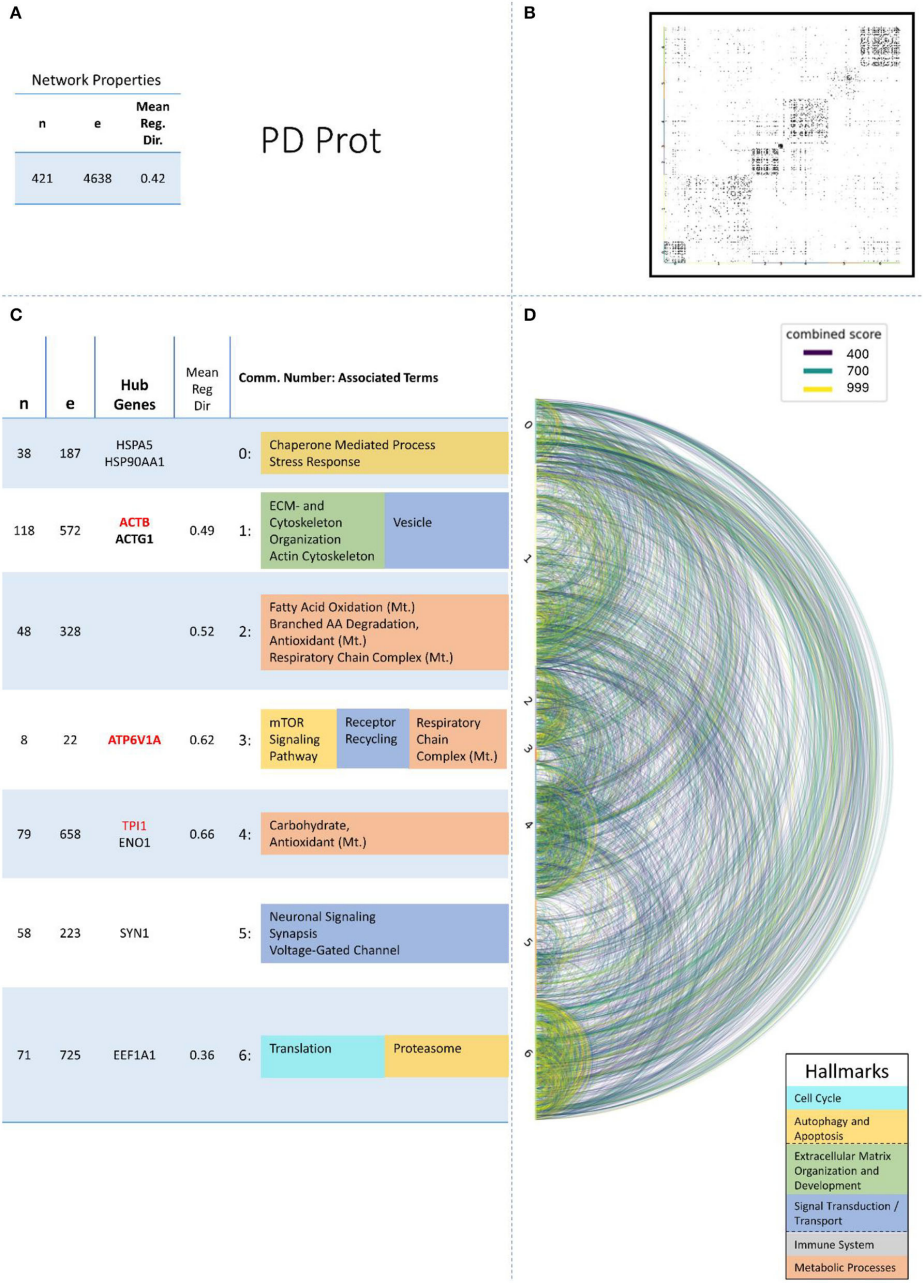
Summarizing figure of the network analysis results of the proteomic layer of PD. The general properties. such as number of nodes (genes) and edges (a combined score > 400 between two nodes) of the PPI network constructed with all PD proteomic genes, are given in Subfigure **(A)**. The individual connections with a combined score > = 400 between each two genes are also given in a matrix plot **(B)** and an Arcplot **(D)** that are ordered by community. The latter shows the confidence of the connection between two genes additionally by the color of the arc. The general properties of each individual community, as well as their hub genes and the categorization of their functional annotation, are given in the table in Subfigure **(C)**. The connected processes are colored by the associated hallmarks that are also displayed in the bottom right corner.

**Figure 6:**
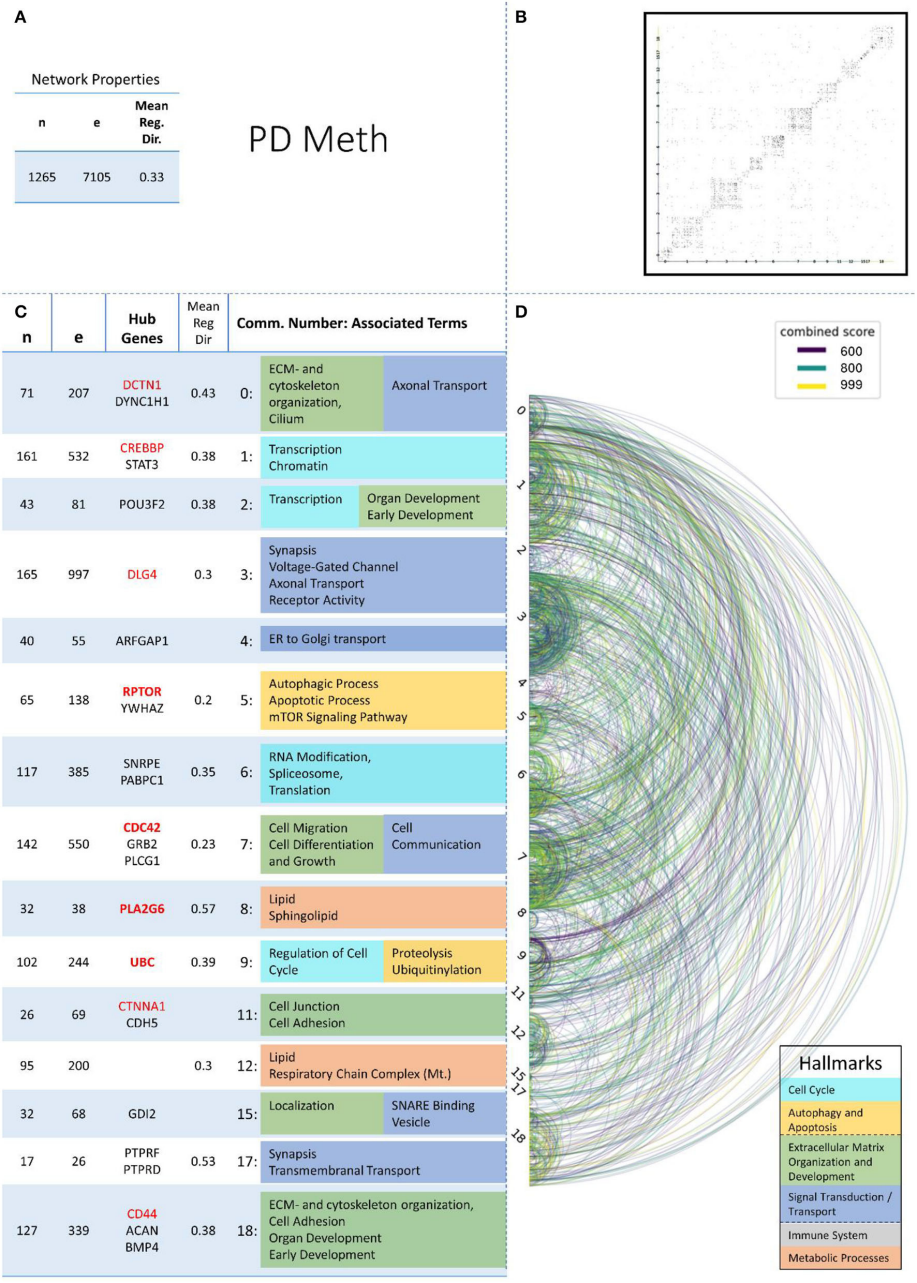
Summarizing figure of the network analysis results of the methylomic layer of PD. The general properties, such as number of nodes (genes) and edges (a combined score > 400 between two nodes) of the PPI network constructed with all PD methylomic genes, are given in Subfigure **(A)**. The individual connections with a combined score > = 600 between each two genes are also given in a matrix plot **(B)** and an Arcplot **(D)** that are ordered by community. The latter shows the confidence of the connection between two genes additionally by the color of the arc. The general properties of each individual community, as well as their hub genes and the categorization of their functional annotation, are given in the table in Subfigure **(C)**. The connected processes are colored by the associated hallmarks that are also displayed in the bottom right corner.

These remaining networks varied between 7 (PD proteome) and 15 (PD methylome) communities with sufficient functional annotation (any biological process with FDR < = 0.05) to be further considered in our analysis. The modularity of these 13 PPI networks varied after community creation between 0.484 (PD proteome) and 0.588 (ALS proteome), thus showing a small deviation in modularity values across the different NDDs and omics-layers. The modularity value is positive if the communities in the network show higher interconnectedness than randomly expected; negative values if there are less connections within the communities than randomly expected are strictly lower than 1 (Equation 3: Modularity). Thus, the 13 networks that we further consider show more connections within their communities than expected by chance (as all have positive modularity values). Examples of those network plots for PD are given in Figures 3–6. The networks for AD, HD, and ALS can be found in the Supplementary Presentation 1—Network Figures.

### Hallmark Definition

The hallmarks of neurodegenerative diseases that we defined based on the functional analyses of the communities are a deregulated **Cell Cycle**, defects in **autophagy and apoptosis** on the cellular level, a defective **Extracellular Matrix Organization and** misguided cell and organ **development**, the impaired **Signal Transduction/Transport** of vital molecules within and between cells within the local tissue environment, an excessive **Immune System** response, and defective **Metabolic Processes** in the whole systemic environment.

The results of the X^2^-test showed that there is a significant difference between the expected and observed values for the four omics-layers across all six hallmarks (*p* < 0.05), but not for the four NDDs across all six hallmarks (see Supplementary Table 3—Low-Level Term Associations).

The most striking results we found when analyzing the accumulated numbers of associations between our communities and low-level terms across the four NDDs and also across the four omics-layers were the low number of associations found in PD for the Immune System hallmark and the low number of associations in the methylomic layer in the Metabolic Processes hallmark while having a high number of associations in the proteomic layer.

A more detailed description of the results is presented in the following;

#### Cell Cycle

In the category of Cell Cycle 56 communities could be assigned to the low-level terms *Regulation of Cell Cycle, Spindle Apparatus, DNA repair, Transcription, RNA Modification, Spliceosome, Translation, Mitochondrial Translation*, and *Chromatin*.

AD showed the most low-level terms (21), followed by PD, HD, and ALS (15, 13, 7), while the transcriptomic and methylomic layers showed a higher number of low-level terms (21, 20) than the proteomic and genomic layers (11 and 4) (see Table 3). In general, for the hallmark Cell Cycle, the numbers of associations between communities and low-level terms neither strongly differed across NDDs nor across omics-layers from the expected values, given the overall distribution of associations across all hallmarks, NDDS, and omics-layers.

**Table 3:** Accumulated number of low-level terms per NDD and per omics-layer for the hallmark Cell Cycle.

		**Per disease**					**Per omics-layer**			
	**Low-level term**	**AD**	**PD**	**ALS**	**HD**	**Total**	**SNP**	**DEG**	**Prot**	**Meth**
Cell cycle	Regulation of cell cycle	2	1	1	1	5	0	2	0	3
	Spindle apparatus	1	0	0	0	1	0	1	0	0
	DNA repair	3	0	0	1	4	1	1	0	2
	Transcription	2	4	1	1	8	1	3	0	4
	RNA modification (degradation, processing, transport)	4	2	1	3	10	1	3	3	3
	Spliceosome	2	2	1	2	7	0	2	3	2
	Translation	3	4	2	3	12	0	5	4	3
	Mitochondrial translation	2	0	0	1	3	0	1	1	1
	Chromatin (organization, binding, modification)	2	2	1	1	6	1	3	0	2
	**Total**	**21**	**15**	**7**	**13**	**56**	**4**	**21**	**11**	**20**
	**Expected**	**22**	**17**	**6**	**11**	**56**	**6**	**20**	**13**	**17**

*The column for the total number of low-level terms found refers to both the sum across all four NDDs and the sum across all four omics-layers. This table is intended to provide summary information about the accumulated data—the number of individual low-level terms per omics-layer for each disease can be found in the Supplementary Table 3. SNP, genomic level; DEG, transcriptomic level; Prot, proteomic level; Meth, methylomic level*.

#### Autophagy and Apoptosis

Of all NDDs and omics-layers, 46 communities were involved in autophagy and apoptosis specified by low-level terms like *Chaperone*-*Mediated Process, Proteolysis, Ubiquitinylation, Proteasome, Stress Response, Apoptotic Process*, and the *mTOR* and *NfkB Signaling Pathways*.

Again, AD showed the most associations (16), followed by PD, HD, and ALS (13, 9, and 8), as shown in Table 4. The expected number for ALS is 5, in contrast to the observed 8 associations, but, overall, the deviations between observation and expectation for the NDDs in this hallmark were quite low.

**Table 4:** Accumulated number of low-level terms per NDD and per omics-layer for the hallmark autophagy and apoptosis.

		**Per disease**					**Per omics-layer**			
	**Low-level term**	**AD**	**PD**	**ALS**	**HD**	**Total**	**SNP**	**DEG**	**Prot**	**Meth**
Autophagy and	Autophagic process	2	2	2	1	7	1	4	1	1
apoptosis	Chaperone mediated process	2	2	1	2	7	0	4	3	0
	Proteolysis	2	2	1	0	5	1	2	1	1
	Ubiquitinylation	1	2	1	2	6	0	3	1	2
	Proteasome	2	1	1	2	6	0	1	4	1
	Stress response	2	1	0	0	3	1	1	1	0
	Apoptotic process	1	1	1	2	5	0	3	0	2
	mTOR signaling pathway	3	2	1	0	6	0	1	3	2
	NfkB signaling pathway	1	0	0	0	1	0	1	0	0
	**Total**	**16**	**13**	**8**	**9**	**46**	**3**	**20**	**14**	**9**
	**Expected**	**18**	**14**	**5**	**9**	**46**	**5**	**16**	**11**	**14**

*The column for the total number of low-level terms found refers to both the sum across all four NDDs and the sum across all four omics-layers. This table is intended to provide summary information about the accumulated data—the number of individual low-level terms per omics-layer for each disease can be found in the Supplementary Table 3. SNP, genomic level; DEG, transcriptomic level; Prot, proteomic level; Meth, methylomic level*.

The distribution of associations across the four omics-layers shows that the transcriptomic data had the most associations (20), followed by the proteomic, methylomic, and genomic layers (14, 9, and 3). Here, the discrepancy between observation and expectation is larger, with the methylomic data showing only 9 associations, whereas 14 are expected, and the transcriptomic and proteomic layers showing 4 and 3 more associations, respectively, than expected.

#### Extracellular Matrix Organization and Development

In total, 80 associations were found between communities and the low-level terms for *Extracellular Matrix (ECM) and Cytoskeleton Organization*, with the different fibril components *Actin -, Collagen -, and Keratin Cytoskeleton, Cilium* as the bridge between ECM and cytoskeleton, *Cell Adhesion, Focal Adhesion, Cell Junction, Cell Migration* along microtubules, *Cell Differentiation and Growth* (e.g., neuron, axon, dendrite, organelle), *Localization* of cells or proteins, *Organ Development* (brain, muscle, heart, bone, blood vessels), and *Early Development* (embryogenesis and cell fate commitment).

The distribution of the number of associated low-level terms across the four NDDs is according to the expectation that can be derived from the summed number of associations across all low-level terms, with AD showing the highest number of associations (33), followed by PD (26), HD (14), and ALS (7), as given in Table 5, with having the highest deviation between observation and expectation for HD.

**Table 5:** Accumulated number of low-level terms per NDD and per omics-layer for the hallmark Extracellular Matrix Organization and Development.

		**Per disease**					**Per omics-layer**			
	**Low-level term**	**AD**	**PD**	**ALS**	**HD**	**Total**	**SNP**	**DEG**	**Prot**	**Meth**
Extracellular matrix organization and	ECM- and cytoskeleton organization	6	5	2	2	15	3	2	4	6
development	Actin cytoskeleton	2	2	2	2	8	0	4	4	0
	Collagen cytoskeleton	2	1	1	1	5	0	4	0	1
	Keratin cytoskeleton	0	0	0	1	1	0	0	1	0
	Cilium	2	3	0	0	5	1	2	0	2
	Cell adhesion	4	3	0	2	9	1	3	2	3
	Focal adhesion	2	0	0	0	2	0	1	0	1
	Cell junction	0	1	0	0	1	0	0	0	1
	Cell migration (along microtubules)	1	2	0	1	4	0	1	0	3
	Cell differentiation and growth (e.g., neuron, axon, dendrite, organelle)	6	1	1	3	11	1	4	1	5
	Localization (cellular, of protein…)	1	1	0	1	3	0	1	0	2
	Organ development (brain, muscle, heart, bone, blood vessels)	4	3	1	0	8	2	2	1	3
	Early development (embryogenesis and cell fate commitment)	3	4	0	1	8	1	3	0	4
	**Total**	**33**	**26**	**7**	**14**	**80**	**9**	**27**	**13**	**31**
	**Expected**	**31**	**24**	**9**	**16**	**80**	**9**	**28**	**19**	**24**

*The column for the total number of low-level terms found refers to both the sum across all four NDDs and the sum across all four omics-layers. This table is intended to provide summary information about the accumulated data—the number of individual low-level terms per omics-layer for each disease can be found in the Supplementary Table 3. ECM, Extracellular Matrix; SNP, genomic level; DEG, transcriptomic level; Prot, proteomic level; Meth, methylomic level*.

However, the distribution of associations across the different omics-layers for the hallmark ECM Organization and Development deviates from the expectation for the proteomic and methylomic layers. The highest number of associations can be observed on the methylomic layer, closely followed by the transcriptomic (31, 27) and then by the proteomic and genomic layers (13, 9). Here, the methylomic layer shows a nearly 25% higher number of associations than expected, while the number for the proteomic layer is nearly 30% lower than expected.

#### Signal Transduction/Transport

This hallmark consisted of the low-level terms *Cell Communication, Neuronal Signaling, Synapsis, Ion*- and *Voltage-Gated Channel, Neurotransmitter Secretion, SNARE Binding, Receptor Activity* and *Recycling, Axonal Transport, Vesicle (exo- and endocytosis, clathrin coat, extracellular exosome), Transmembranal Transport (small molecules, e.g., AA), Trans-Golgi-Network (retrograde), ER to Golgi transport (anterograde)*, and *(Cardiac) Muscle Action Potential*.

The hallmark Signal Transduction/Transport united the most associations between communities and low-level terms (88), having the most associations in AD (34), followed by PD (31), HD (17), and ALS (6). Compared to the number of expected associations, ALS showed a reduction of 4 associations (−40%) and PD, an increase of 5 associations (approximately + 20%) compared to the expectation.

Observing the results per omics-layer, it becomes apparent that not the transcriptomic but the methylomic layer showed the most associations for this hallmark (31). The number of associations in the transcriptomic layer is lower than the methylomic layer's associations (24), followed by the proteomic (18) and genomic layers (15), as given in Table 6. While the transcriptomic layer shows surprisingly little associations (– 22%), the genomic layer, on opposite, shows a surprisingly high number of associations (+50%), in contrast to the expectation.

**Table 6:** Accumulated number of low-level terms per NDD and per omics-layer for the hallmark Signal Transduction/Transport.

		**Per disease**					**Per omics-layer**			
	**Low-level term**	**AD**	**PD**	**ALS**	**HD**	**Total**	**SNP**	**DEG**	**Prot**	**Meth**
Signal transduction/	Cell communication	4	4	2	3	13	3	3	2	5
transport	Neuronal signaling	1	1	0	0	2	0	1	1	0
	Synapsis	6	5	0	2	13	3	3	3	4
	Ion channel	1	0	0	2	3	0	1	0	2
	Voltage-gated channel	4	2	0	1	7	1	2	0	4
	Neurotransmitter secretion	2	1	0	2	5	1	2	2	0
	SNARE binding	2	3	1	1	7	1	2	3	1
	Receptor activity	3	1	0	0	4	2	0	0	2
	Receptor recycling	1	1	0	0	2	0	0	2	0
	Axonal Transport	0	2	0	1	3	0	0	0	3
	Vesicle (exo- endocytosis, clathrin coat, extracellular exosome)	5	6	2	2	15	2	4	5	4
	Transmembranal transport (small molecules, e.g., AA)	2	3	0	1	6	1	3	0	2
	Trans-Golgi-network (retrograde)	1	1	1	1	4	0	2	0	2
	ER to Golgi transport (anterograde)	1	1	0	0	2	0	1	0	1
	(Cardiac) muscle action potential	1	0	0	1	2	1	0	0	1
	**Total**	**34**	**31**	**6**	**17**	**88**	**15**	**24**	**18**	**31**
	**Expected**	**34**	**26**	**10**	**18**	**88**	**10**	**31**	**21**	**27**

*The column for the total number of low-level terms found refers to both the sum across all four NDDs and the sum across all four omics-layers. This table is intended to provide summary information about the accumulated data—the number of individual low-level terms per omics-layer for each disease can be found in the Supplementary Table 3. SNP, genomic level; DEG, transcriptomic level; Prot, proteomic level; Meth, methylomic level; AA, amino acids*.

A closer look at all low-level terms in relation to the individual NDD's omics-layers (see Supplementary Table 3—Low-Level Term Associations) also shows that two out of the three associations for *Axonal Transport* are found in the methylomic PD network, and two out of the four *Receptor Activity* associations are found in the AD genomic network.

Looking at the aggregated data per layer in each omics-layer also shows a high number of genomic PD associations (8 out of 88 for Signal Transduction/Transport), far exceeding the overall 4% proportion of low-level terms associated with the PD genomic layer.

At the same time, the proportion of transcriptomic ALS communities associated with the low-level terms of these hallmarks (2 of 88) is only half the proportion that might be expected based on the overall distribution of low-level terms associated with the transcriptomic ALS layer (>5%).

#### Immune System

Only 28 associations between communities and the low-level terms related to Immune System were found, making it the hallmark with the lowest number of associations. Low-level terms, which were referred to by these communities, were *Immune Response, Defense Response to Other Organisms, Cytokine, Interferon, Complement and Coagulation Cascade, Inflammation*, and *Leukocyte Activation*.

The highest number of associations was found in AD (13), followed by HD (7), ALS (5), and PD (3), making this the only hallmark in which PD showed the lowest number of associations (see Table 7). This number of associations found across all omics-layers in PD is more than 60% lower than expected.

**Table 7:** Accumulated number of low-level terms per NDD and per omics-layer for the hallmark Immune System.

		**Per disease**					**Per omics-layer**			
	**Low-level term**	**AD**	**PD**	**ALS**	**HD**	**Total**	**SNP**	**DEG**	**Prot**	**Meth**
Immune system	Immune response	1	1	1	1	4	0	2	0	2
	Defense response to other organisms	2	1	1	1	5	1	3	0	1
	Cytokine	2	0	0	1	3	1	0	0	2
	Interferon	2	1	1	2	6	1	3	0	2
	Complement and coagulation cascade	2	0	2	2	6	0	3	3	0
	Inflammation	2	0	0	0	2	0	1	1	0
	Leukocyte activation	2	0	0	0	2	1	0	1	0
	**Total**	**13**	**3**	**5**	**7**	**28**	**4**	**12**	**5**	**7**
	**Expected**	**11**	**8**	**3**	**6**	**28**	**3**	**10**	**7**	**9**

*The column for the total number of low-level terms found refers to both the sum across all four NDDs and the sum across all four omics-layers. This table is intended to provide summary information about the accumulated data—the number of individual low-level terms per omics-layer for each disease can be found in the Supplementary Table 3. SNP, genomic level; DEG, transcriptomic level; Prot, proteomic level; Meth, methylomic level*.

The findings in the aggregation per omics-layers seem less surprising, with the transcriptomic layer showing the most associations (12), followed by the methylomic (7), proteomic (5), and genomic layers (4).

#### Metabolic Processes

The hallmark Metabolic Processes consisted of the low-level terms *Lipid, Cholesterol, Sphingolipid, Fatty acid oxidation* [*mitochondrial (Mt.)*]*, Carboxylic Acid (Mt.), Lipoprotein, Protein, Carbohydrate*, and *Nucleotide*, most of which referred to the respective metabolic processes and of terms like *Glycosylation, NADH (Mt.) Homeostasis, Respiratory Chain Complex (Mt.)*, and *Antioxidant (Mt.)*, which were tagged with the “Mt.” term if they represent processes that occur in the mitochondrion rather than in the cytoplasm.

Looking at the accumulated associations between communities and low-level terms across the four different NDDs (see Table 8), the most associations can be found across the communities of AD (26), followed by PD (20), HD (14), and ALS (7). This distribution across NDDs perfectly matches the expected distribution of associations.

**Table 8:** Accumulated number of low-level terms per NDD and per omics-layer for the hallmark Metabolic Processes.

		**Per disease**					**Per omics-layer**			
	**Low-level term**	**AD**	**PD**	**ALS**	**HD**	**Total**	**SNP**	**DEG**	**Prot**	**Meth**
Metabolic processes	Lipid	2	2	0	1	5	0	2	0	3
	Cholesterol	2	1	0	1	4	2	1	0	1
	Sphingolipid	1	2	0	0	3	0	2	0	1
	Fatty acid oxidation (Mt.)	2	1	0	3	6	0	1	3	2
	Carboxylic acid (Mt.)	1	0	1	1	3	0	1	1	1
	Lipoprotein	2	0	1	1	4	1	0	3	0
	Protein	0	0	1	0	1	0	1	0	0
	Branched AA degradation	2	2	0	1	5	0	3	2	0
	Proteoglycan	1	2	0	0	3	1	2	0	0
	Carbohydrate	3	2	1	2	8	0	2	4	2
	Glycosylation	1	0	0	0	1	0	1	0	0
	Nucleotide	3	1	0	0	4	1	1	1	1
	NADH (Mt.)	1	0	0	0	1	0	0	1	0
	Homeostasis	1	0	0	1	2	0	1	1	0
	Antioxidant (Mt.)	2	3	1	0	6	0	2	4	0
	Respiratory chain complex (Mt.)	2	4	2	3	11	0	4	5	2
	**Total**	**26**	**20**	**7**	**14**	**67**	**5**	**24**	**25**	**13**
	**Expected**	**26**	**20**	**7**	**14**	**67**	**7**	**23**	**16**	**20**

*The column for the total number of low-level terms found refers to both the sum across all four NDDs and the sum across all four omics-layers. This table is intended to provide summary information about the accumulated data—the number of individual low-level terms per omics-layer for each disease can be found in the Supplementary Table 3. Mt, mitochondrial; SNP, genomic level; DEG, transcriptomic level; Prot, proteomic level; Meth, methylomic level; AA, amino acids*.

However, the distribution across the four omics-layers is more conspicuous, as Metabolic Processes are the only hallmark having the majority of the associations in the proteomic layer (25). This is closely followed by the transcriptomic (24) and then the methylomic and genomic layers (13 and 5). Following the overall distribution of associations across the omics-layers, only 16, instead of 25, associations were expected across the proteomic layers (+ 56%) of all NDDs, but 20, instead of 13, associations were expected for the methylomic layers of all NDDs (−35%).

The individual omics-layers per NDD showed that, especially, the proteomic layer of PD and AD had enriched numbers of associations for this hallmark, both with having more than 33% of their community associations to the low-level term in Metabolic Processes, while, overall, only ~18% were expected.

## Discussion

### Omics-Layer Intersections per Disease

The intersection of genes found in genomic, transcriptomic, proteomic, and methylomic data per disease showed that, while the number of shared genes between the transcriptomic and proteomic level was significantly high in all four investigated NDDs, the significance of the intersections was more heterogenous in the other comparisons.

Only AD showed a significant number of shared genes in all pairwise intersections of the four analyzed omics-layers. The fact that ALS methylomic and HD genomic data consisted of only two studies and thus had a very small database (61 and 35 genes) may have contributed to the non-significance of pairwise comparisons that considered the methylomic level for ALS and the genomic layer for HD. However, even though the database was much broader for HD methylomic data (885 genes), these genes also stem from only two studies, but showed significant overlaps in all pairwise intersections, except for the one with genomic data. Summarizing, especially those pairwise comparisons with a low number of expected overlapping genes, seemed to not show a significantly large pairwise overlap. All pairwise intersections that are based on two large groups of genes and, therefore, showed an expected number of shared genes > = 10 also showed a significantly high number of overlapping genes in these hypergeometric tests (Supplementary Table 2—Hypergeom Test Per Disease).

The fact that the effect size was highest for the transcriptomic-proteomic intersection in AD, ALS, and HD and, also, very high in PD (9.9) seems as if these layers shared the most striking relation in terms of overlapping genes. However, in PD, the effect size is even higher between the transcriptomic and methylomic layers. PD is also the only disease in which not proteomic and transcriptomic layers showed the largest database in terms of including the most genes but the transcriptomic and methylomic data did. Thus, in all NDDs, especially those intersections, showed the largest effect sizes that were based on the omics-layers, with the most genes per NDD. The statement that the transcriptomic and proteomic layers of our data showed high significantly enriched numbers of genes in their overlap in all analyzed NDDs holds true; however, it is, to this point, unclear if the high effect sizes in contrast to the other intersections are due to biological facts or to the broader database for these omics-layers in our data.

A more surprising point is the fact that the mean direction of regulation was vastly unequal to zero for both, the transcriptomic and the proteomic data in PD. This was also the case for the methylomic layer of HD and ALS, but, given that the transcriptomic and proteomic layers of PD are based on much more studies (11 and 7, while 2 studies for HD and ALS methylome), this high absolute mean direction of regulation, in combination with the dichotomy of these values, constitutes an interesting finding. The finding of a mainly (70%) negative direction of regulation for PD transcriptomic differentially expressed genes was already described in a meta-study, focusing only on microarray data (Kelly et al., 2019). However, the reason for this phenomenon remains unclear.

### Multi-Omics Conformity

The finding of significant overlaps between the transcriptomic and proteomic data for only three out of four NDDs seems to be surprising and to hint at an extraordinary phenomenon in the posttranscriptional or translational processes in PD. However, given that the mean direction of regulation for the PD transcriptomic data was highly negative (−0.59), while the PD proteomic data were, overall, highly positive (0.42), it is striking that the genes in the intersection of these two omics-layers are still positively correlated, even though not significantly. Thus, this result might show a signal for some altered posttranscriptional behavior in PD in contrast to the other NDDs, but this could also be an artifact of the striking negative mean direction of regulation in the transcriptomic and the similarly strong positive mean direction of regulation in the proteomic data that were discussed earlier.

However, even the highly significant correlations between the transcriptomic and proteomic data in AD, HD, and ALS reached values of r = 0.283 (AD) to r = 0.344 (ALS), which might seem surprisingly small, following James Watson's simplified version of the central dogma of molecular biology (Watson et al., 1988).

Even though proteins are produced from mRNA, the correlation between mRNA levels and protein abundances is moderate. This non-trivial mRNA–protein relation is a general phenomenon, ranging from yeast to humans (De Sousa Abreu et al., 2009), and can have biological or, simply, technical reasons. Biological reasons can be found both on the posttranscriptional and on the translational levels, where many different mechanisms enhance or repress the synthesis of proteins from a certain copy number of mRNA molecules (Crick, 1958; Varshavsky, 1997; Fire et al., 1998; Kozak, 1999; Glickman and Ciechanover, 2002; Pillai et al., 2007; Kurreck, 2009; Maier et al., 2009; Chursov et al., 2013; Komar, 2016; Liu et al., 2016; McShane et al., 2016; Lau et al., 2018).

### Protein-Protein Interaction Networks

#### The Hub Gene HDAC1

On the transcriptomic level of all 4 diseases communities were found, that showed Histone Deacetylase 1 (*HDAC1*) as one of their hub genes. HDAC1 deacetylates lysine residues on the N-terminal part of the core histones H2A, H2B, H3, and H4, thereby contributing to epigenetic silencing of active chromatin (Carroll et al., 2018). HDAC1 plays an important role in transcriptional regulation, cell cycle progression, and developmental events. This was also reflected in the low-level terms of the associated communities of all four diseases: In AD and PD, they referred to transcription, chromatin, and early development, together with the hubgenes *TP53, EP300*, and *MYC*, respectively. In ALS, the low-level terms referred to regulation of cell cycle, transcription, and chromatin, together with the hub gene *CCND1*. HD showed low-level terms about cell differentiation, cell and early development, together with the hubgenes *MYC* and *TP53*.

All the hubgenes that were related to *HDAC1* are involved in similar processes like regulation of cell cycle, cell growth and migration, and development by influencing the chromatin formation (*EP300*), binding to DNA as transcription factors (*TP53, MYC*) or regulating other proteins (*CCND1*). Beyond the aforementioned similarities, researchers can, maybe, find differences in how the diseases developed by comparing the functions of the hub genes and their StringDB protein networks per disease.

#### The Hub Genes BIN1, ABCA7, and PICALM

*BIN1, ABCA7*, and *PICALM* were found to be among the three most central nodes in, at least, three of our four centrality measures and were, therefore, defined as hub genes in our analysis for the AD genomic community that was associated with the two processes, proteolysis and ECM/cytoskeleton. Approximately, 38 genes were involved in this network, showing 284 interactions giving an average node degree (*N*_*d*_) of 14.9. Mutations in these three genes are well-known risk factors in AD, and all the three of them are tightly connected to amyloid ß metabolism.

BIN1 or Bridging Integrator-1 is a key player in the control of plasma membrane shaping. The depletion of BIN1 increases cellular β-site APP-cleaving enzyme 1 (BACE1) levels through impaired endosomal trafficking and reduced lysosomal degradation, resulting in an increased Aβ production.

ABCA7 is a member of the superfamily of ATP-binding cassette (ABC) transporters, which transport various molecules across extra- and intra-cellular membranes. As with BIN1, ABCA7 could be shown to be involved in the generation and processing of amyloid ß peptides (Satoh et al., 2015), but also in the lipid metabolism and in phagocytosis and immune response (Dib et al., 2021).

PICALM, the phosphatidylinositol-binding clathrin assembly protein, also affects AD risk primarily by modulating production, transportation, and clearance of β-amyloid (Aβ) peptide, but other Aβ-independent pathways are discussed, including tauopathy (Dean and Shaw, 2010), synaptic dysfunction (Jahn and Scheller, 2006), disorganized lipid metabolism (Eisenstein, 2011), immune disorder (Carter, 2010), and disrupted iron homeostasis (Xu et al., 2014).

These genes, together with the other 35 genes found to interact closely with the two low-level terms proteolysis and ECM/cytoskeleton, can now be studied in more detail by specialists in the field with respect to the relationship between the known functions of the genes and the processes in which they are involved. This combination of all three genes was only found in AD, and any of these three was found as a hub gene only in the AD genomic network.

However, *BIN1* was also present in the transcriptomic [community 0 (C0)], proteomic (C1), and methylomic (C7) levels of PD, representing mainly ECM and Development and Signal Transduction/Transport processes but also one for Autophagy and Apoptosis. *PICALM* was also part of C0 in the PD transcriptomic network. In HD, the proteomic network showed both, *BIN1* and *PICALM* in C0, which represent Signal Transduction/Transport processes like SNARE Binding and Vesicle. In ALS, none of these genes were involved in any considered community.

#### The Hub Gene APOE

*APOE*, the well-known risk gene for late onset AD, was found on the genomic level in AD in a community associated with the low-level terms cholesterol and lipoprotein, but also on the transcriptomic level of ALS associated with the low-level terms complement/coagulation cascade and immune response.

The complement cascade stimulates phagocytes to clear microbes and damaged cell materials, it promotes inflammation, and attacks the pathogen's cell membrane. It, thus, combines innate and adaptive immune responses and additionally integrates the coagulation system into the defense response against other organisms (Kenawy et al., 2015). In addition, some of the early components of this cascade play a beneficial role in synapse elimination during the development of the nervous system, although excessive complement-mediated synaptic pruning in the adult or injured brain may be detrimental in multiple neurogenerative disorders (Schartz and Tenner, 2020). A correlation between ALS and the complement system has been frequently described (Carpanini et al., 2019; Kjældgaard et al., 2021), but a connection to APOE still remains elusive.

Here, our study may provide a good starting point for exploring the community to which *APOE* belongs and the genes to which it is linked in this network. The fact that *APOE* is among the top three nodes according to all four measures of centrality implies that it plays an important role in this network, as it is among the three most interconnected genes, has direct links to other very important genes in the network, and plays an important role in mediating between pairwise-linked genes in the network, while it is also among the three nodes that have the shortest paths to all other nodes in the network. Although *APOE* has not been previously reported to be associated with the complement and coagulation cascades, it is strongly associated with a subnetwork of genes that are significantly overrepresented in this very process. About one third of the directly linked 19 genes are directly involved in the complement cascade (*C1QB, C1QC, C3, C4A, C4B, CFH*); another third is involved in cholesterol synthesis and transport (*ABCA1, ABCB1, HMGCR, LDLR, MSR1, NPC2*); A2M disrupts inflammatory cascades but also is involved in the lipoprotein metabolism; BACE2, CTSB, and PSEN are involved in the proteolytic processing of the amyloid precursor protein; CP is a metalloprotein that binds most of the copper in plasma and is involved in the peroxidation of Fe (II) transferrin to Fe (III) transferrin. PCYOX1 cleaves the thioether bond of prenyl-L-cysteines, and PSRC1 may participate in p53-mediated growth suppression.

### Discussion of Methodological Approach

We attempted to limit the data included in this study to those derived from brain tissue whenever possible. For this reason, and because of the generally greater availability of data, we analyzed data in this study mainly from patients in the late stages of their respective NDD. This results in better comparability and less noise within the data but should be considered in the analysis and conclusions drawn from these studies, as effects occurring mainly in early stages of disease progression may not be apparent in this analysis.

It should also be noted that we included only data of studies that were published and, furthermore, available in PubMed, Google Scholar, the GWAS Catalog (Buniello et al., 2019), the GEO database (Barrett et al., 2013), the Expression Atlas (Papatheodorou et al., 2018), PDMethDB (Wang C. et al., 2020) or the EWAS Atlas (Li M. et al., 2019). Therefore, as pointed out in Pan et al. (2005), this introduces some language bias, as our methodology did not include searching for results published in other than the English language in, e.g., local journals, which was shown to influence the strength of *p*-values between, e.g., Chinese and non-Chinese studies (Pan et al., 2005; Tang, 2005). Also, we did not consider unpublished as well as unsignificant data. However, as our approach was mainly aimed for giving a comprehensive and broad overview and, therefore, mainly focused on those genes that were found as significant in, at least, two experiments, regardless of the exact strength of the actual *p*-value, we consider this problem of publication bias as a minor one for our approach as the number of overexaggerated and false-positive disease associations should be strongly reduced by this methodology.

For some approaches used in this study, it was necessary to choose between different possible approaches, e.g., for the hallmarks to be used or the definition of hub genes. In addition, the network creation of the louvain algorithm is non-deterministic and, therefore, subject to a random influence in the creation of the networks. To minimize this random influence, we applied the louvain algorithm with the input shuffled 100 times anew for each of the analyzed networks, and then chose the iteration that produced the highest modularity. With this approach, we found that the modularity between the different iterations did not change much; however, taking the best of 100 runs makes the results of the louvain community creation more reliable.

The definition of hallmarks and hub genes involves some flexibility, so we have attempted to define hallmarks in light of previous definitions of hallmarks for neurodegeneration (e.g., Ramanan and Saykin, 2013) and of our actual observed subprocesses in the functional analyses. As there are myriads of centrality measurements for nodes in a network, we took centrality measurements into account that were connected to the number of direct links between a node and other nodes, the mediation role of a node in a network, and to the sum of the shortest paths to all other nodes in the network, respectively (Freeman, 1977, 1978; Freeman et al., 1979). In addition, we chose to include the eigenvector centrality of each node (Newman, 2010; Bonacich, 2015), which gives a centrality score to each node that is proportional to the sum of the centrality scores of each node's neighbors to cover some varieties of ways for defining centrality of a node in a network.

## Conclusion

The results of this study present a comprehensive picture drawn from the results of the analysis of 234 studies across four neurodegenerative disorders and four omics layers. Roughly summarizing, the 69 low-level terms we chose to categorize into 6 hallmarks show a significantly different distribution across the different omics layers, but not across the four diseases, which might be due to the inclusion of mainly late-stage disease data for broadening the database. The number of genes in the intersection of two diseases and the correlation of their regulatory direction was mainly distinctive between the transcriptomic and proteomic levels.

However, the possibilities for further analysis arising from this study are, at least, as important as the results just mentioned. For example, the communities found, and the processes associated with them can be compared with regard to the direction of regulation of the communities, the presence and absence of processes at certain levels/diseases, or, also, with regard to the hub genes found, as we have already exemplified with three examples in the discussion. Consequently, we not only provide an overview—the big picture of neurodegeneration—but also many new starting points for further research going deeper into individual communities or even genes within these communities. We achieve this by providing comprehensive illustrations as well as all relevant tables for exploring specific communities and their associated genes. Ultimately, identifying the hub genes and the other genes involved in the different clusters may make it easier to develop therapeutics by both getting a better idea of which protein can be targeted in a chosen process but, at the same time, whether too many other important processes could be disturbed by the potential inhibition or amplification.

## Data Availability Statement

The original contributions presented in the study are included in the article/Supplementary Materials, further inquiries can be directed to the corresponding author.

## Author Contributions

NR and SK wrote the manuscript, designed the research question, performed the analysis, wrote code, interpreted the data, and visualized the results. SS and RH contributed to writing, interpreting the data, and editing the manuscript. SG conceptualized the research, contributed to writing and interpretation, edited the manuscript, supervised the study, and acquired the funding. All authors have read and agreed to the published version of the manuscript. All authors contributed to the article and approved the submitted version.

## Funding

This research was partially funded by the ReALity initiative—Resilience, Adaptation, and Longevity (SG and SS). NR and RH acknowledge funding of the Leibniz Institute for Resilience Research (LIR) gGmbH and the IDSAIR initiative.

## Conflict of Interest

The authors declare that the research was conducted in the absence of any commercial or financial relationships that could be construed as a potential conflict of interest.

## Publisher's Note

All claims expressed in this article are solely those of the authors and do not necessarily represent those of their affiliated organizations, or those of the publisher, the editors and the reviewers. Any product that may be evaluated in this article, or claim that may be made by its manufacturer, is not guaranteed or endorsed by the publisher.
