# Supplementary material for: The Big Picture of Neurodegeneration: A Meta Study to Extract the Essential Evidence on Neurodegenerative Diseases in a Network-Based Approach
Source: Front Aging Neurosci. 2022 Jun 27;14:866886. doi: 10.3389/fnagi.2022.866886 (PMC9271745; doi:10.3389/fnagi.2022.866886)
Supplement: Supplementary file 5 [file Presentation_1.zip › network_figures.pdf]

A

Network Properties

| n | e | Mean Reg. Dir. |
|---|---|---|
| 521 | 1821 | n.a. |

AD SNP

B

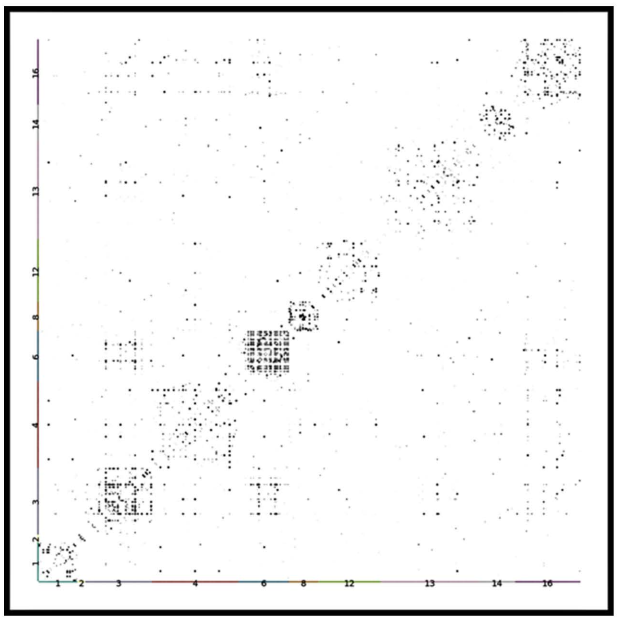


C

| n | e | Hub Genes | Comm. Number: Associated Terms | | |
|---|---|---|---|---|---|
| 29 | 45 | PSMA1 | 1: | Synapsis Receptor Activity | |
| 6 | 7 | CACNA1G CATSPERB KCNN2 | 2: | Voltage-Gated Channel (Cardiac) Muscle Action Potential | |
| 49 | 126 | APOB APOE APP | 3: | Cholesterol, Lipoprotein | |
| 65 | 122 | CDH1 **ITGA6** CASP3 | 4: | ECM- and cytoskeleton organization Cell Adhesion Organ Development | |
| 38 | 284 | BIN1 ABCA7 PICALM | 6: | Proteolysis | ECM- and cytoskeleton organization |
| 22 | 52 | **ATM** **ERCC2** ERCC1 | 8: | DNA Repair | Stress Response |
| 47 | 61 | BIRC3 **MADD** | 12: | Autophagic Process | |
| 74 | 141 | GRIN2B | 13: | Cell Differentiation and Growth | Synapsis Receptor Activity |
| 25 | 31 | EGR1 ADAR | 14: | RNA Modification, Chromatin | Interferon |
| 48 | 113 | IL6 SYK | 16: | Cell Communication | Defense Response to other Organisms Cytokine Leukocyte Activation |

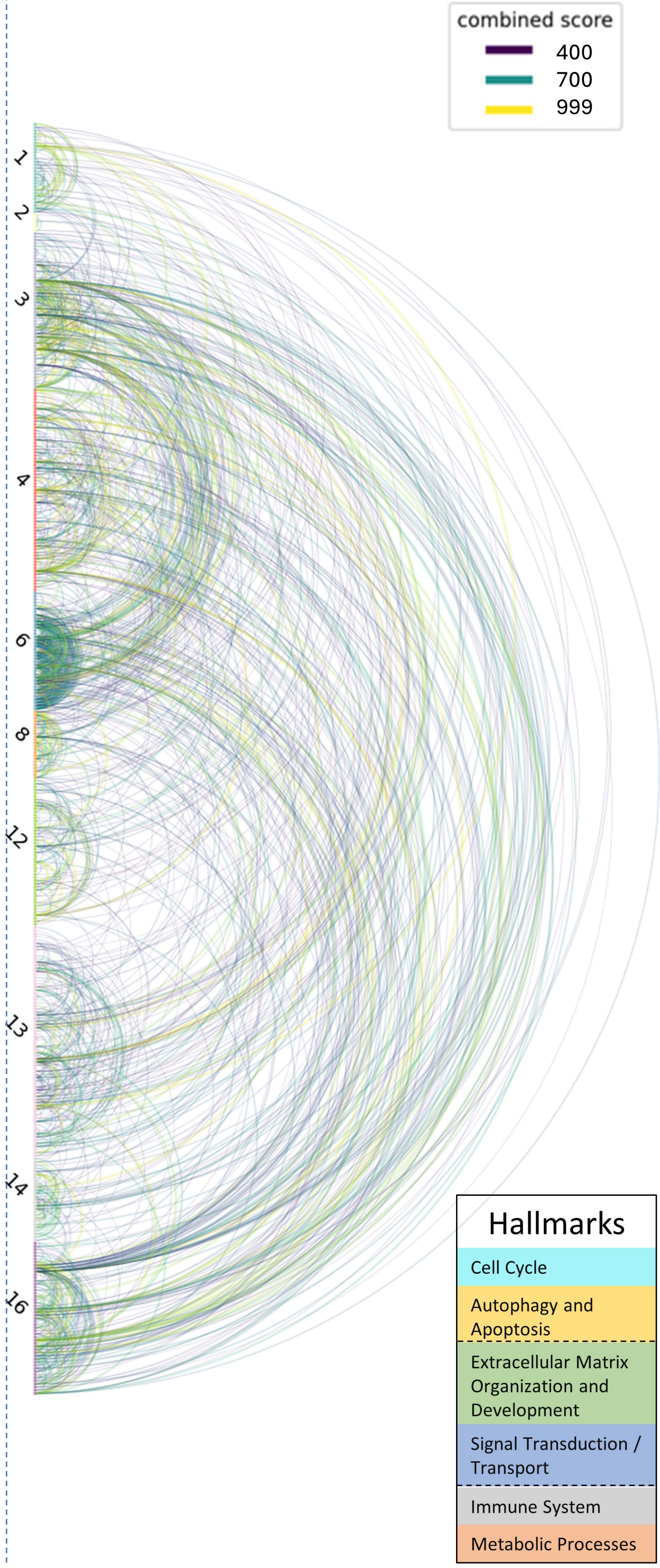

A

Network Properties

| n | e | Mean Reg. Dir. |
|---|---|---|
| 14654 | 649843 | 0. 13 |

AD DEG

B

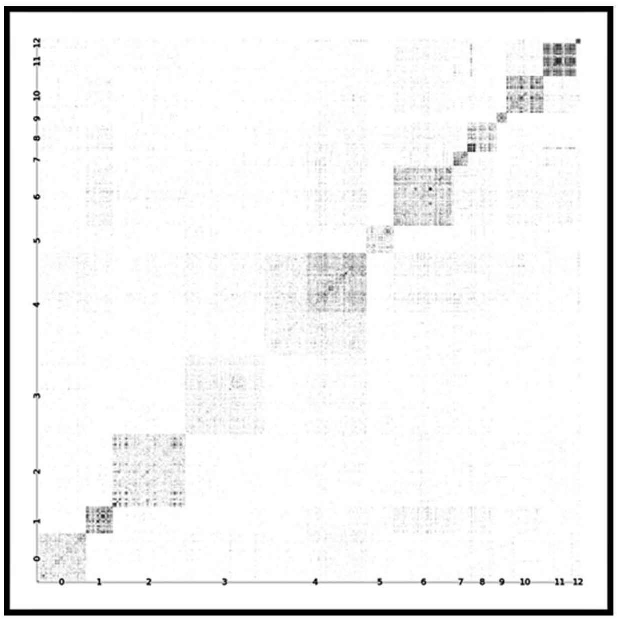

C

| n | e | Hub Genes | Mean Reg Dir | Comm. Number: Associated Terms |
|---|---|---|---|---|
| 1278 | 24662 | RAB5A<br>RAB7A | 0.04 | 0: Auto-phagic Process; Local-ization; SNARE Binding, Vesicle, ER to Golgi transport |
| 683 | 22915 | | 0.16 | 1: Regulation of Cell Cycle, Spindle Apparatus, DNA Repair, Chromatin, |
| 1852 | 40486 | CAT | 0.05 | 2: Trans-membranal Transport; Lipid, Branched AA Degradation, Carbohydrate, Nucleotide, Antioxidant (Mt.), Respiratory Chain Complex (Mt.) |
| 2060 | 34465 | | 0.06 | 3: Neuronal Signaling, Synapsis, Voltage-Gated Channel, Neurotransmitter Secretion, |
| 2705 | 77466 | SRC | 0.33 | 4: Apoptotic Process, NfkB signaling pathway; Actin Cytosk. Collagen Cytosk. Cell Adhesion Cell Diff. & Growth; Defense Resp. to other Organisms, Inflammation, Compl. & Coag. Cascade |
| 694 | 7377 | IFT88 | 0.14 | 5: Spindle Apparatus; ECM- and cytoskeleton organization, Cilium |
| 1575 | 38572 | EP300<br>**TP53**<br>HDAC1 | 0.23 | 6: Transcription, Chromatin |
| 357 | 4728 | PPP2CA<br>PPP2R1A | 0.14 | 7: Focal Adhesion, Cell Differentiation and Growth, Organ Development, Early Development |
| 759 | 13937 | UBC<br>UBB | | 8: Proteolysis, Ubiquitinylation, Proteasome |
| 267 | 2615 | | 0.16 | 9: Sphingolipid, Proteoglycan, Glycosylation |
| 957 | 28314 | SNRPD3 | 0.09 | 10: Transcription, RNA Modification , Spliceosome; Chaperone Mediated Process |
| 831 | 47025 | RPS5 | -0.08 | 11: Translation, Mitochondrial Translation |
| 119 | 2064 | | | 12: Stress Response, mTOR Sig. pathway |

D

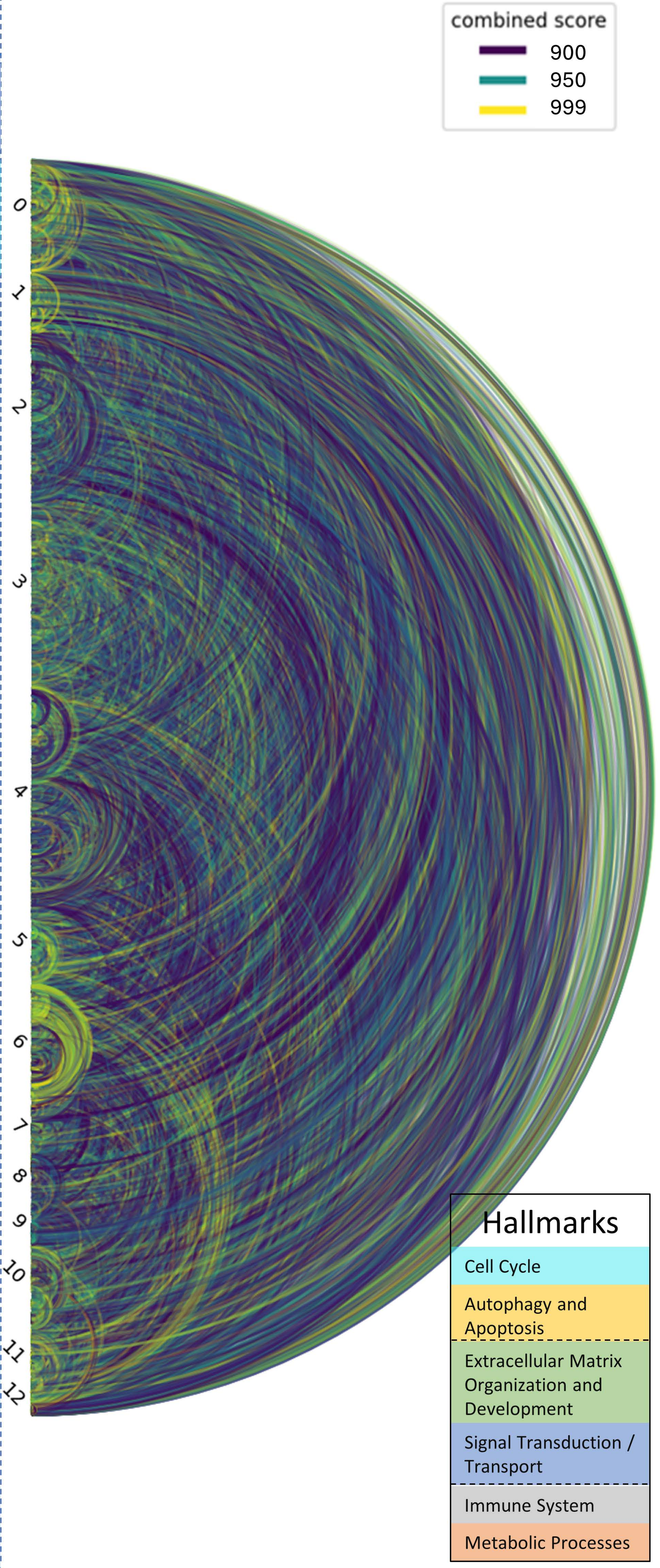

A

Network Properties

| n | e | Mean Reg. Dir. |
|---|---|---|
| 1956 | 40482 | -0.10 |

# AD Prot

B

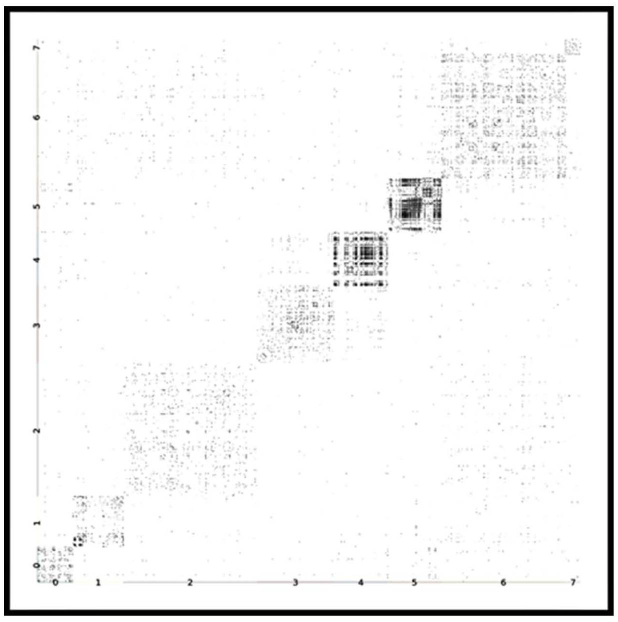

C

| n | e | Hub Genes | Mean Reg Dir | Comm. Number: Associated Terms | | |
|---|---|---|---|---|---|---|
| 127 | 985 | | | 0: | RNA Modification, Spliceosome | Chaperone Mediated Process |
| 181 | 1224 | CLTC | -0.13 | 1: | mTOR Signaling Pathway | Vesicle Receptor Recycling |
| 481 | 5317 | **SNAP25** DLG4 | -0.2 | 2: | Synapsis Neurotransmitter Secretion SNARE Binding | |
| 277 | 3119 | TPI1 | 0.13 | 3: | Fatty Acid Oxidation (Mt.) Carboxylic Acid (Mt.), Branched AA Degradation, Carbohydrate, Nucleotide, Antioxidant (Mt.) | |
| 193 | 3965 | UQCRFS1 | -0.55 | 4: | NADH (Mt.), Respiratory Chain Complex (Mt.) | |
| 195 | 4714 | RPL9 | -0.31 | 5: | Translation Mitochondrial Translation | Proteasome |
| 444 | 5790 | MAPK3 CDC42 | 0.13 | 6: | ECM- and cytoskeleton organization, Actin Cytoskeleton Cell Adhesion Organ Development | Cell Communication |
| 54 | 406 | **ALB** C3 | -0.27 | 7: | Inflammation Complement and Coagulation Cascade Leukocyte Activation | Homeostasis |

D

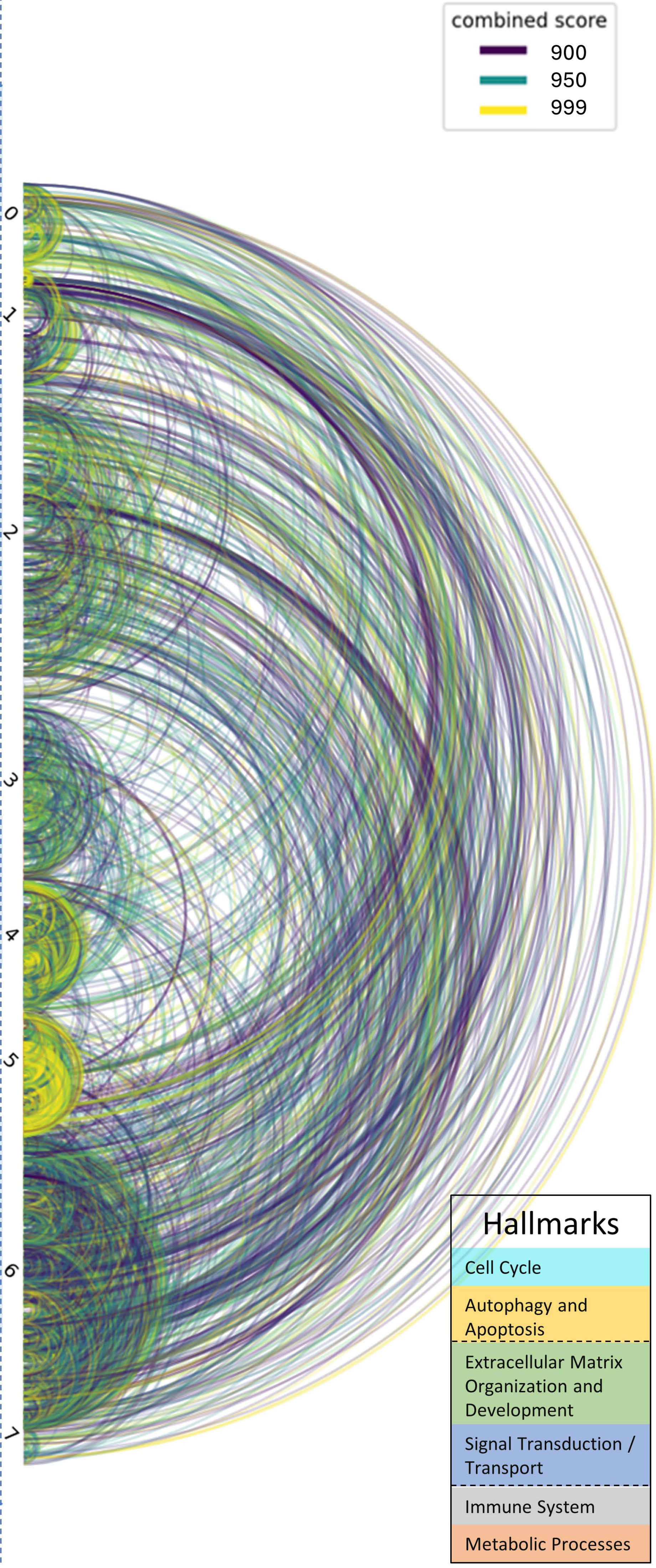

A

Network Properties

| n | e | Mean Reg. Dir. |
|---|---|---|
| 1172 | 6019 | -0.06 |

# AD Meth

B

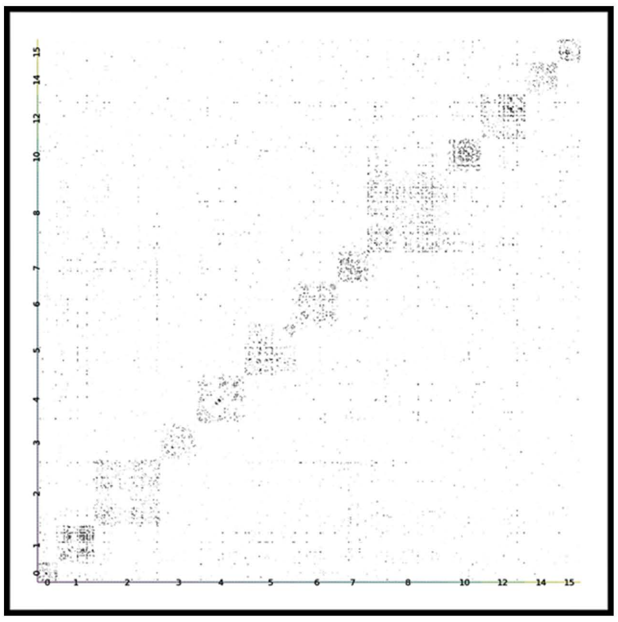


C

| n | e | Hub Genes | Mean Reg Dir | Comm. Number: Associated Terms |
|---|---|---|---|---|
| 39 | 81 | **SORT1**<br>DNM2 | | 0: Vesicle<br>Trans-Golgi-Network |
| 75 | 235 | PLCG1<br>GRB2 | | 1: Cell Adhesion \| Immune Response<br>Cytokine<br>Interferon |
| 131 | 555 | | | 2: Cell Differentiation and Growth \| Cell Communication<br>Synapsis<br>Voltage-Gated Channel<br>Receptor Activity |
| 75 | 111 | PFKL | -0.24 | 3: Carbohydrate,<br>Nucleotide |
| 97 | 221 | **SREBF1**<br>LEP<br>SCD | | 4: Lipid ,<br>Cholesterol,<br>Fatty Acid Oxidation (Mt.) |
| 104 | 315 | NTN1<br>DOCK1 | | 5: Focal Adhesion<br>Cell Differentiation and Growth \| Cell Communication |
| 84 | 191 | **FN1**<br>**VWF** | | 6: ECM- and cytoskeleton organization<br>Collagen Cytoskeleton<br>Cell Migration \| Vesicle |
| 61 | 168 | CACNA1C<br>**CACNA1D** | | 7: Synapsis<br>Voltage-Gated Channel<br>Transmembranal Transport |
| 162 | 719 | **NOTCH1**<br>SOX2<br>HDAC2 | | 8: Transcription \| Cell Differentiation and Growth<br>Early Development |
| 66 | 221 | **ATM**<br>RPA2 | | 10: Regulation of Cell Cycle<br>DNA Repair |
| 89 | 259 | RPS14<br>RPL4 | | 12: RNA Modification,<br>Translation |
| 67 | 128 | PCNT | | 14: ECM- and cytoskeleton organization<br>Cilium \| Vesicle |
| 46 | 84 | SMAD7 | | 15: mTOR Signaling Pathway \| Organ Development<br>Early Development |

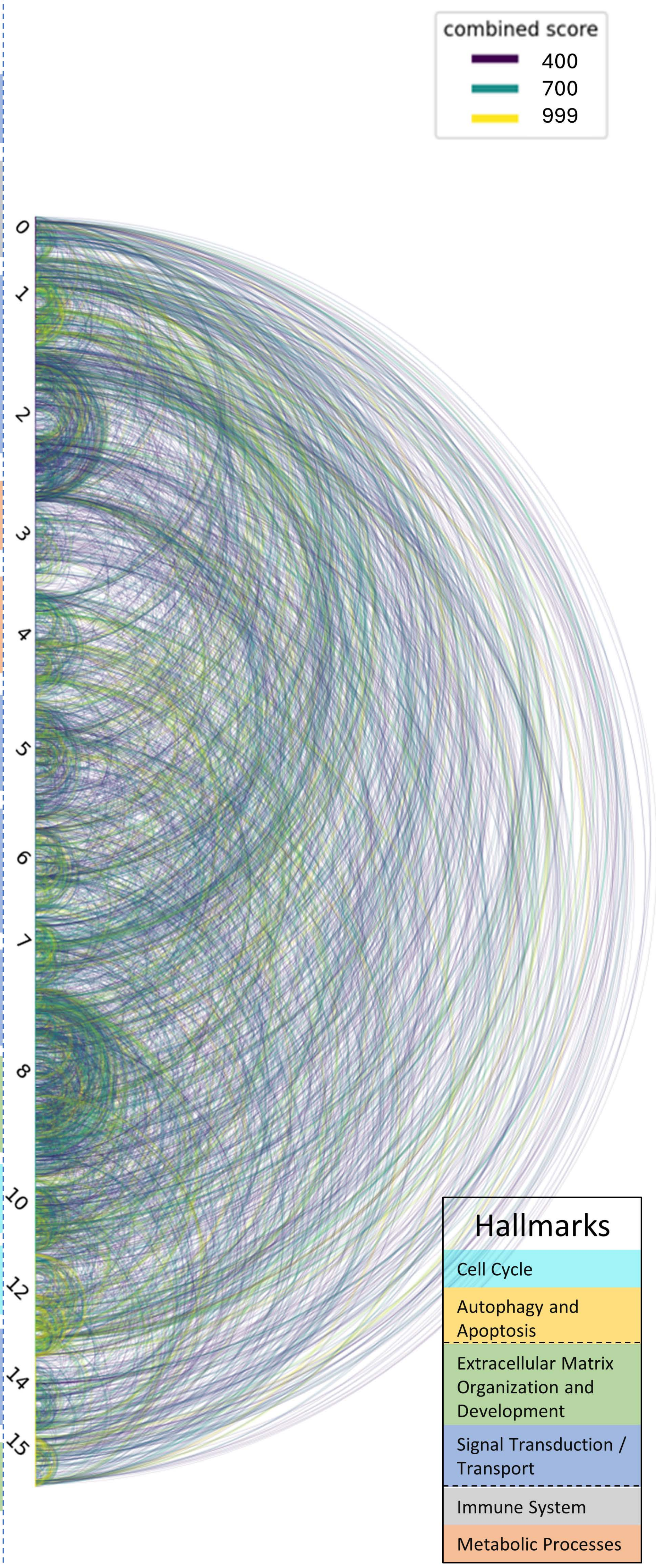

A

Network Properties

| n | e | Mean Reg. Dir. |
|---|---|---|
| 246 | 629 | n.a. |

PD SNP

B

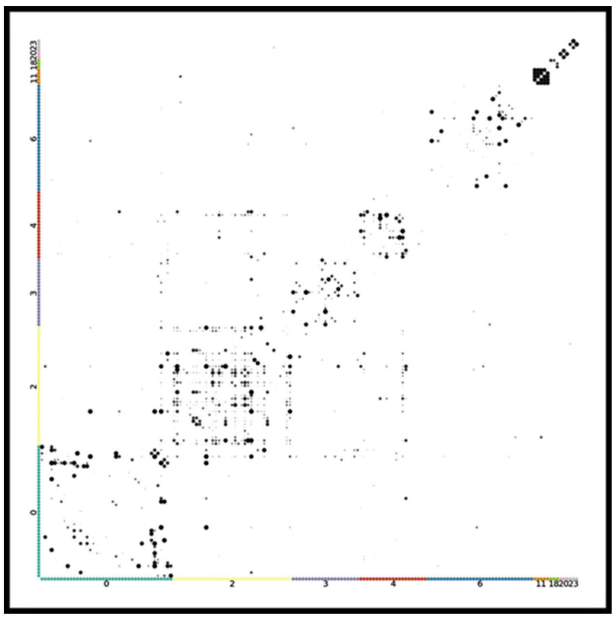

C

| n | e | Hub Genes | Comm. Number: Associated Terms |
|---|---|---|---|
| 40 | 69 | **FYN**<br>MAPT | 0: Cell Communication<br>Transmembranal Transport |
| 37 | 210 | LRRK2<br>CCDC62 | 2: Synapsis<br>Vesicle |
| 21 | 34 | **KANSL1** | 3: Early Development / Cell Communication |
| 21 | 38 | STX1B<br>VAMP4<br>UNC13B | 4: Neurotransmitter Secretion<br>SNARE Binding<br>Vesicle |
| 33 | 38 | **POLR2A** | 6: Transcription / Nucleotide |
| 5 | 10 | **ADAMTS16**<br>**THSD4** | 11: Organ Development |
| 3 | 2 | * | 18: ECM- and cytoskeleton organization, Cilium |
| 3 | 2 | * | 20: Proteoglycan |
| 3 | 2 | * | 23: Cholesterol |

D

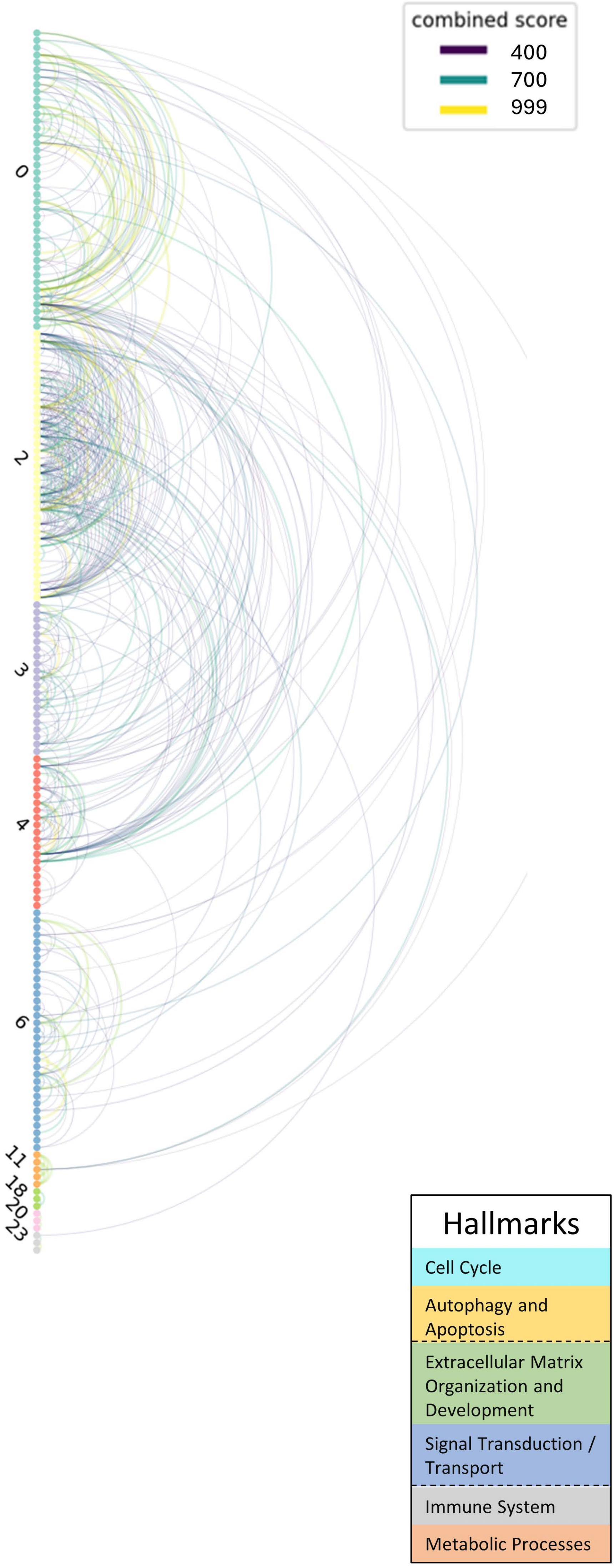


Hallmarks

- Cell Cycle
- Autophagy and Apoptosis
- Extracellular Matrix Organization and Development
- Signal Transduction / Transport
- Immune System
- Metabolic Processes

A

| Network Properties | | |
|---|---|---|
| **n** | **e** | **Mean Reg. Dir.** |
| 4793 | 90412 | -0.59 |

PD DEG

B

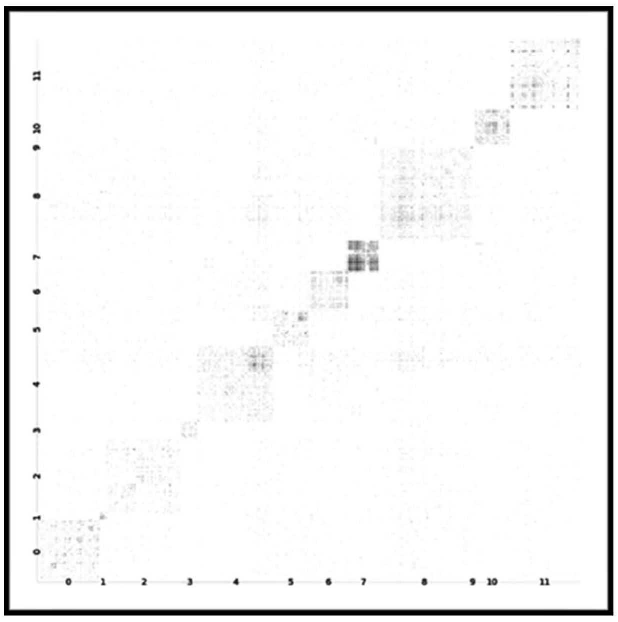

C

| n | e | Hub Genes | Mean Reg Dir | Comm. Number: Associated Terms |
|---|---|---|---|---|
| 550 | 4916 | VAMP2 ARF1 | -0.64 | 0: Autophagic Process; SNARE Binding, Vesicle, Trans-Golgi-Network |
| 60 | 266 | HLA-C | -0.57 | 1: Immune Response Defense Response to Other Organism, Interferon |
| 655 | 5759 | | -0.67 | 2: Cell Communication Synapsis, Voltage-Gated Channel |
| 148 | 535 | | -0.6 | 3: Sphingolipid, Proteoglycan |
| 660 | 8015 | **HDAC1** **MYC** | -0.52 | 4: Transcription, Chromatin; Early Development |
| 308 | 2287 | HSP90AA1 | -0,.63 | 5: Chaperone Mediated Process; ECM- & cytoskeleton organization, Cilium; Vesicle |
| 354 | 3087 | **UBC** UBB UBA52 | -0.58 | 6: Proteolysis, Ubiquitinylation |
| 259 | 5977 | RPS5 | -0.55 | 7: Translation, Ribosome |
| 813 | 10173 | FN1 | -0.57 | 8: Actin Cytoskeleton, Collagen Cytoskeleton, Cell Adhesion, Cell Migration |
| 32 | 145 | **SLC7A5** SLC1A7 | -0.39 | 9: Transmembranal Transport |
| 311 | 3601 | **HNRNPC** SNRPE | -0.5 | 10: RNA Modification, Spliceosome, Translation |
| 617 | 6270 | ACO2 | -0.65 | 11: Branched AA Degradation, Carbohydrate, Antioxidant (Mt.), Respiratory Chain Complex (Mt.) |

D

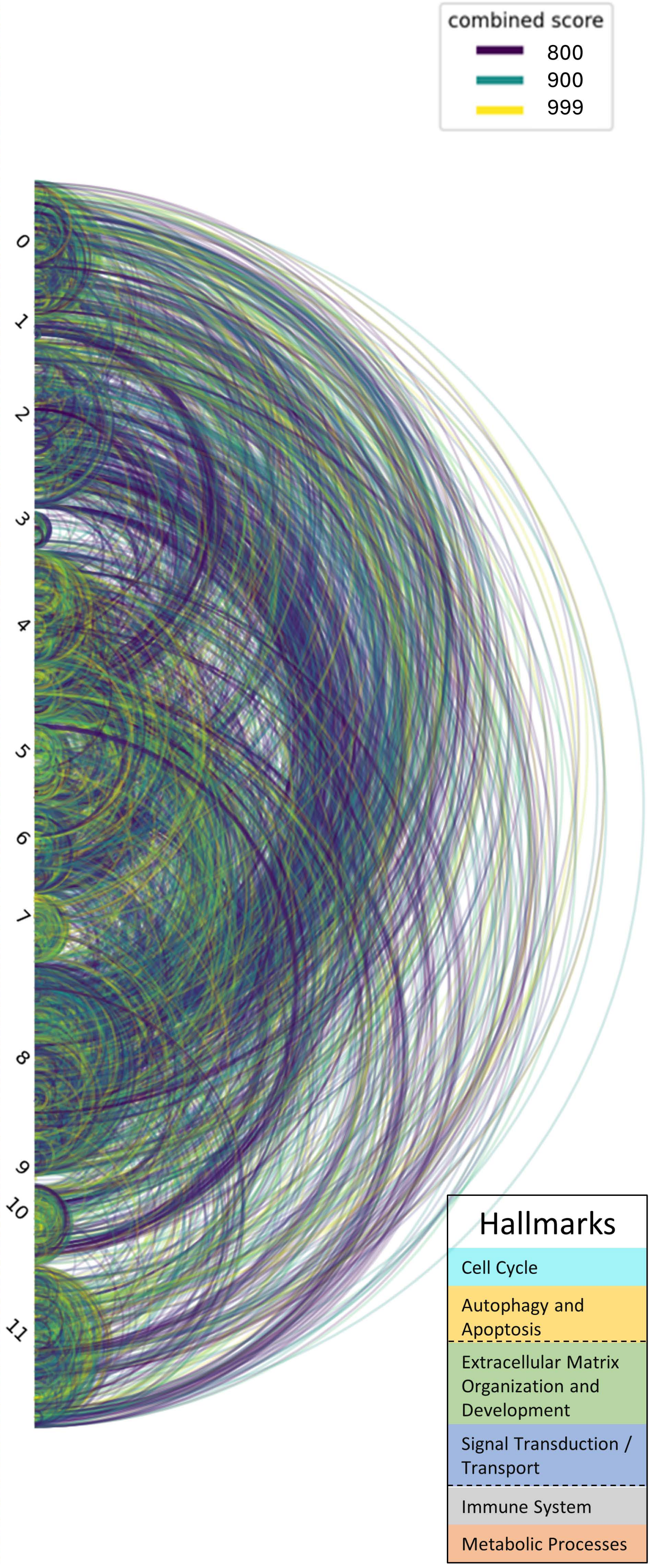

A

Network Properties

| n | e | Mean Reg. Dir. |
|---|---|---|
| 421 | 4638 | 0.42 |

PD Prot

B

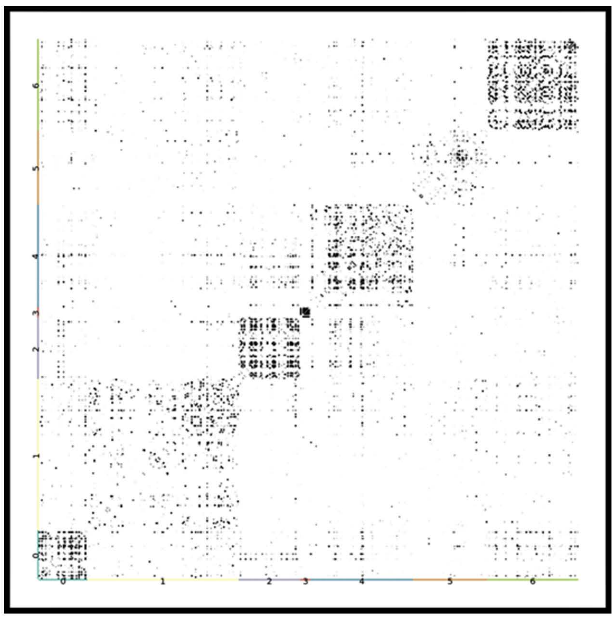

C

| n | e | Hub Genes | Mean Reg Dir | Comm. Number: Associated Terms |
|---|---|---|---|---|
| 38 | 187 | HSPA5 HSP90AA1 | | 0: Chaperone Mediated Process; Stress Response |
| 118 | 572 | **ACTB** **ACTG1** | 0.49 | 1: ECM- and Cytoskeleton Organization; Actin Cytoskeleton; Vesicle |
| 48 | 328 | | 0.52 | 2: Fatty Acid Oxidation (Mt.); Branched AA Degradation, Antioxidant (Mt.); Respiratory Chain Complex (Mt.) |
| 8 | 22 | **ATP6V1A** | 0.62 | 3: mTOR Signaling Pathway; Receptor Recycling; Respiratory Chain Complex (Mt.) |
| 79 | 658 | TPI1 ENO1 | 0.66 | 4: Carbohydrate, Antioxidant (Mt.) |
| 58 | 223 | SYN1 | | 5: Neuronal Signaling; Synapsis; Voltage-Gated Channel |
| 71 | 725 | EEF1A1 | 0.36 | 6: Translation; Proteasome |

D

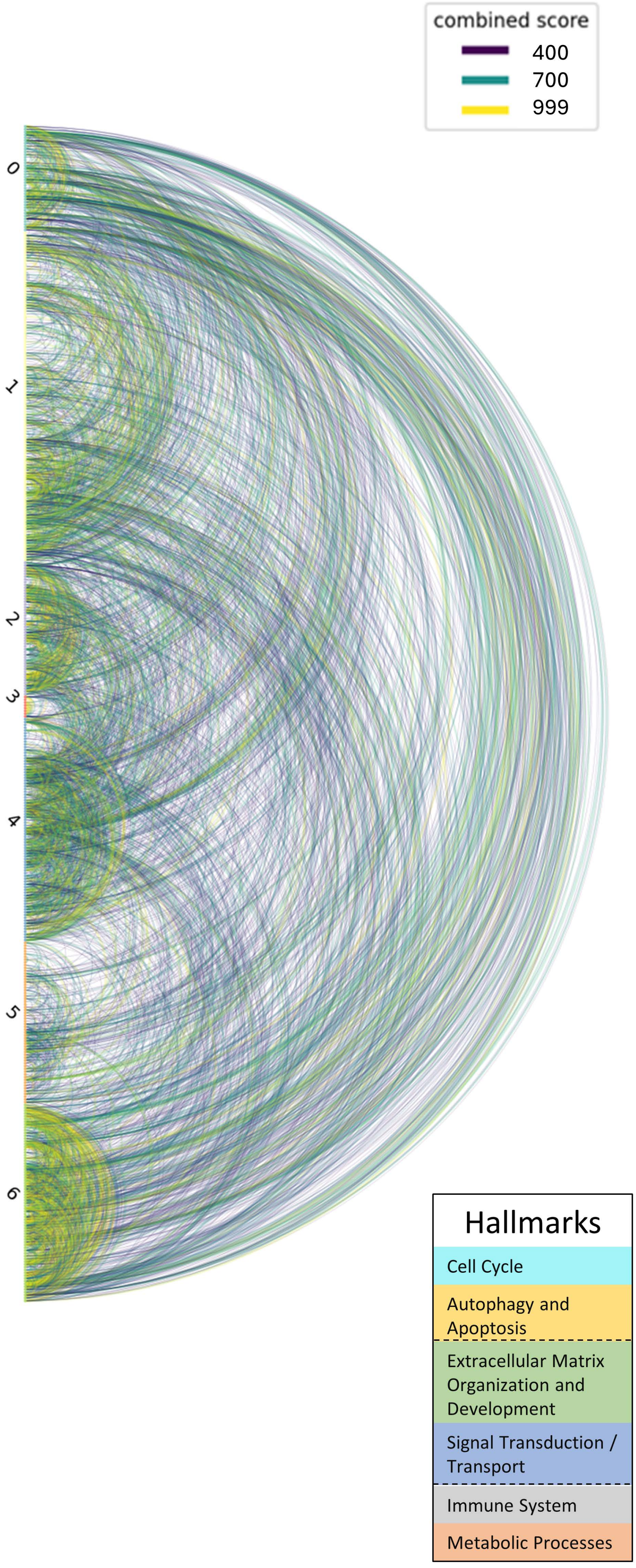

A

Network Properties

| n | e | Mean Reg. Dir. |
|---|---|---|
| 1265 | 7105 | 0.33 |

PD Meth

B

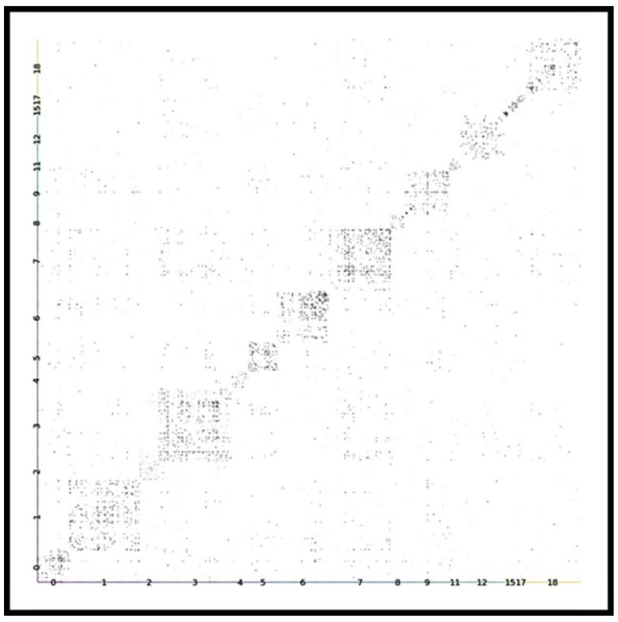

C

| n | e | Hub Genes | Mean Reg Dir | Comm. Number: Associated Terms | | |
|---|---|---|---|---|---|---|
| 71 | 207 | DCTN1 DYNC1H1 | 0.43 | 0: | ECM- and cytoskeleton organization, Cilium | Axonal Transport |
| 161 | 532 | CREBBP STAT3 | 0.38 | 1: | Transcription Chromatin | |
| 43 | 81 | POU3F2 | 0.38 | 2: | Transcription | Organ Development Early Development |
| 165 | 997 | DLG4 | 0.3 | 3: | Synapsis Voltage-Gated Channel Axonal Transport Receptor Activity | |
| 40 | 55 | ARFGAP1 | | 4: | ER to Golgi transport | |
| 65 | 138 | **RPTOR** YWHAZ | 0.2 | 5: | Autophagic Process Apoptotic Process mTOR Signaling Pathway | |
| 117 | 385 | SNRPE PABPC1 | 0.35 | 6: | RNA Modification, Spliceosome, Translation | |
| 142 | 550 | **CDC42** GRB2 PLCG1 | 0.23 | 7: | Cell Migration Cell Differentiation and Growth | Cell Communication |
| 32 | 38 | **PLA2G6** | 0.57 | 8: | Lipid Sphingolipid | |
| 102 | 244 | **UBC** | 0.39 | 9: | Regulation of Cell Cycle | Proteolysis Ubiquitinylation |
| 26 | 69 | CTNNA1 CDH5 | | 11: | Cell Junction Cell Adhesion | |
| 95 | 200 | | 0.3 | 12: | Lipid Respiratory Chain Complex (Mt.) | |
| 32 | 68 | GDI2 | | 15: | Localization | SNARE Binding Vesicle |
| 17 | 26 | PTPRF PTPRD | 0.53 | 17: | Synapsis Transmembranal Transport | |
| 127 | 339 | CD44 ACAN BMP4 | 0.38 | 18: | ECM- and cytoskeleton organization, Cell Adhesion Organ Development Early Development | |

D

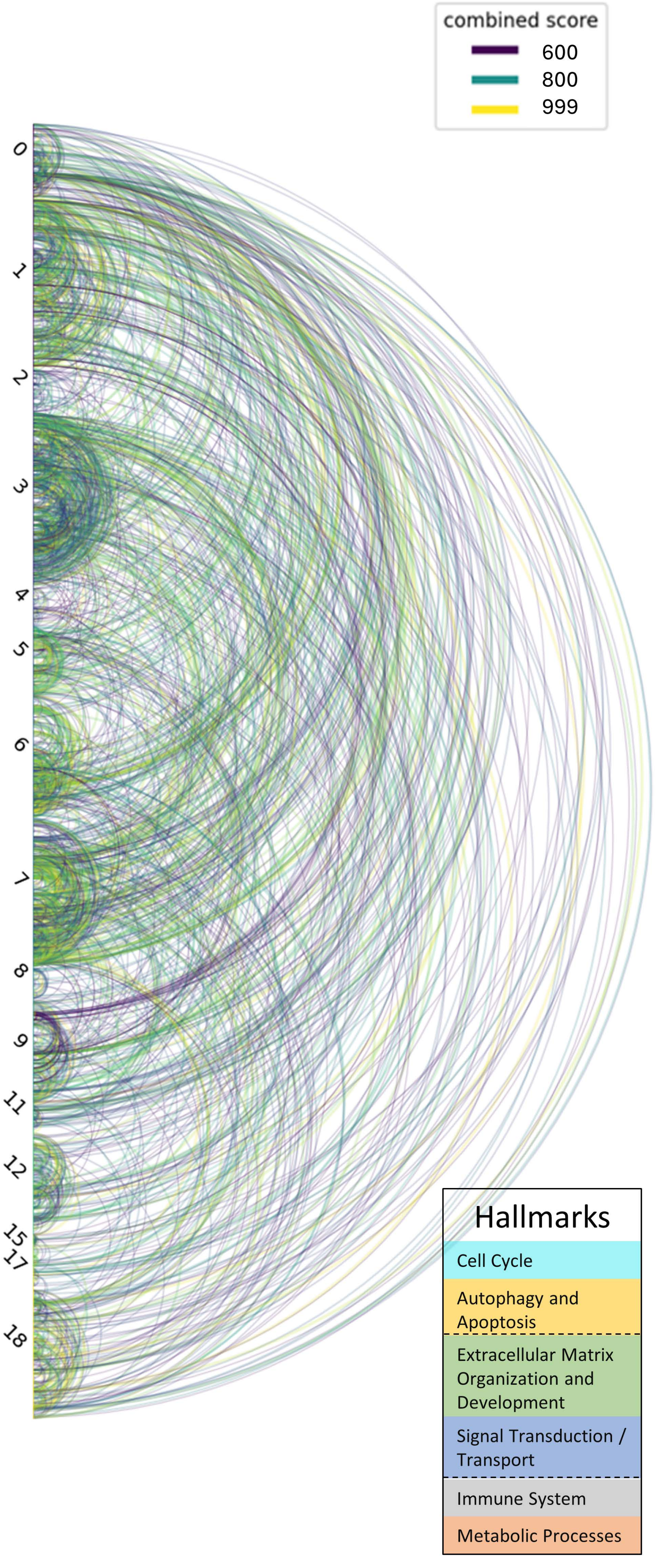

A

ALS DEG

Network Properties

| n | e | Mean Reg. Dir. |
|---|---|---|
| 853 | 5315 | 0. 12 |

B

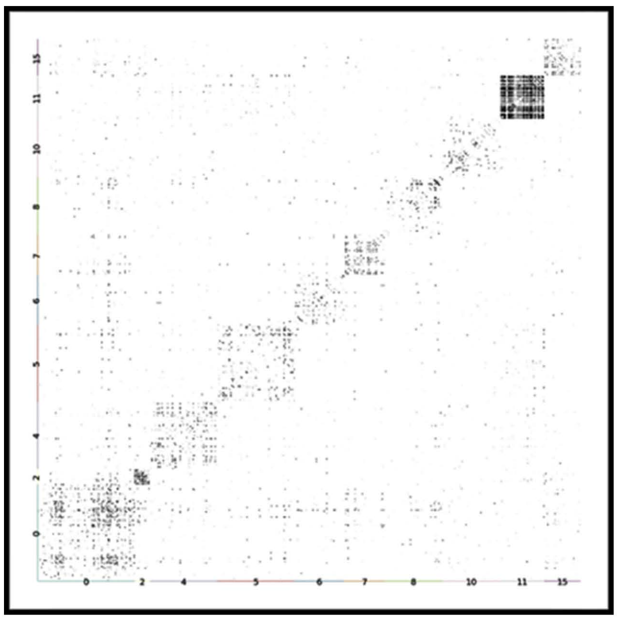

C

| n | e | Hub Genes | Mean Reg Dir | Comm. Number: Associated Terms |
|---|---|---|---|---|
| 148 | 929 | **STAT3** CD4 | 0.35 | 0: Apoptotic Process |
| 24 | 89 | HLA-B ISG15 | 0.5 | 2: Defense Response to Other Organism, Interferon |
| 103 | 288 | **HSPA4** HSP90AB1 | | 4: Autophagic Process, Chaperone Mediated Process; Trans-Golgi-Network |
| 119 | 274 | **HDAC1** CCND1 | | 5: Regulation of Cell Cycle, Transcription, Chromatin |
| 76 | 143 | **ACTN1** FLNA | | 6: Actin Cytoskeleton |
| 63 | 219 | **COL1A1** COL4A1 COL4A2 | 0.36 | 7: Collagen Cytoskeleton, Organ Development |
| 89 | 166 | **RAF1** AKT2 | | 8: Cell Communication; Protein |
| 89 | 152 | VAMP2 | -0.14 | 10: Carboxylic Acid (Mt.), Respiratory Chain Complex (Mt.) |
| 67 | 881 | RPS6 | | 11: Translation |
| 56 | 140 | APOE | 0.21 | 15: Complement and Coagulation Cascade, Immune Response |

D

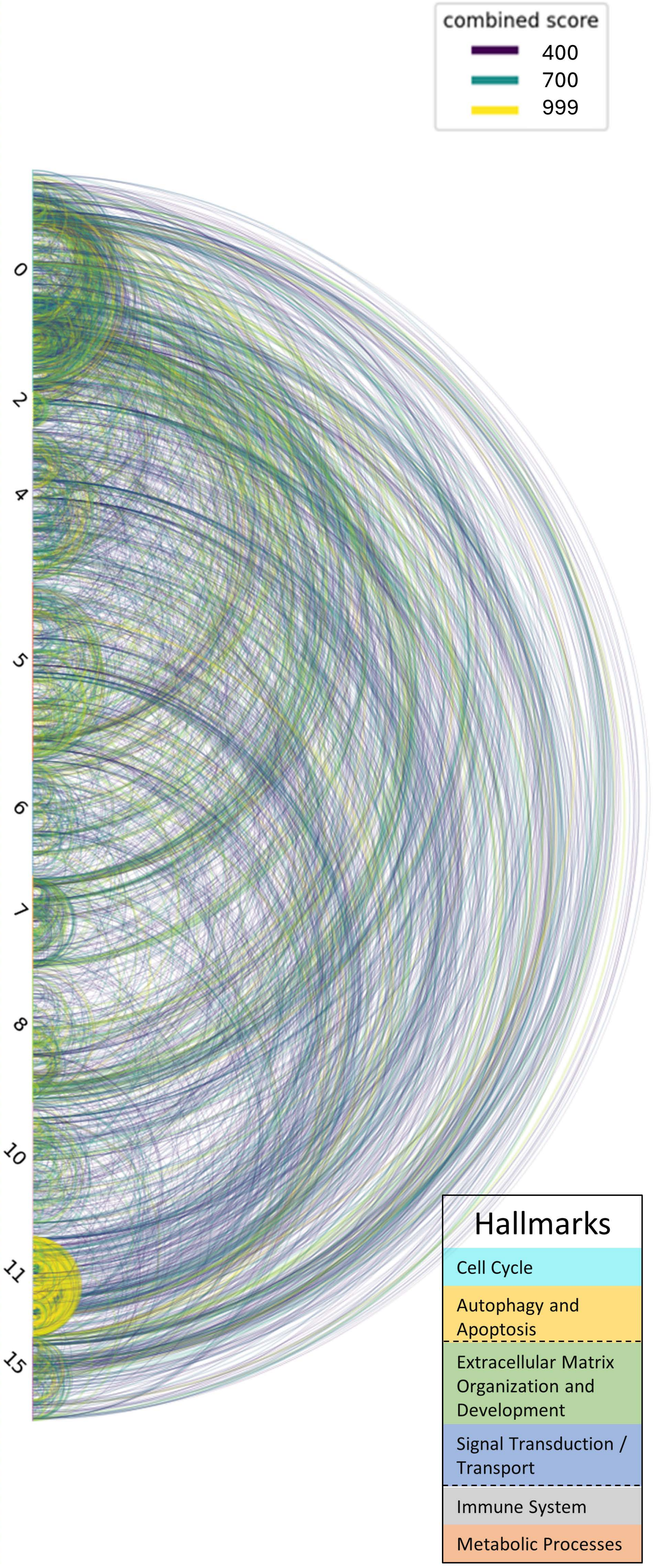

A

Network Properties

| n | e | Mean Reg. Dir. |
|---|---|---|
| 1756 | 27735 | 0. 16 |

ALS Prot

B

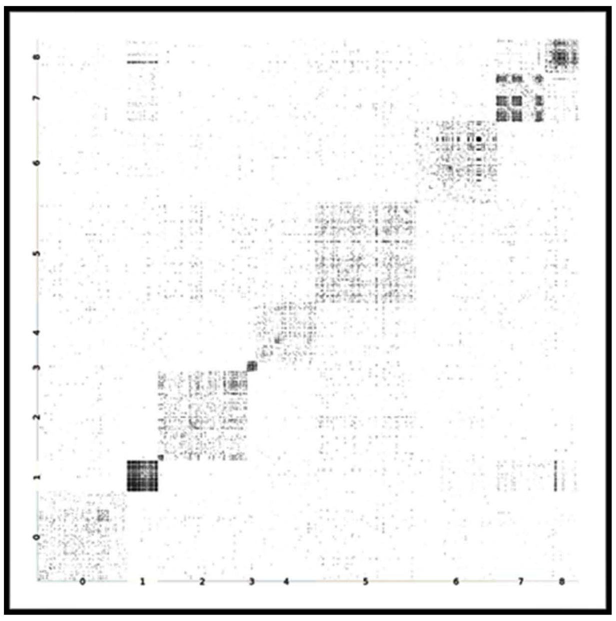

C

| n | e | Hub Genes | Mean Reg Dir | Comm. Number: Associated Terms |
|---|---|---|---|---|
| 292 | 1601 | CLTC CANX | | 0: SNARE Binding Vesicle |
| 98 | 2283 | | | 1: Translation |
| 286 | 3497 | **ALB** | 0.74 | 2: ECM & Cyto-skeleton Organization; Complement & Coagulation Cascade; Lipoprotein |
| 32 | 197 | **RPTOR** | | 3: Autophagic Process mTOR Signaling Pathway; Cell Differentiation and Growth |
| 190 | 974 | DCTN1 DYNC1H1 | -0.24 | 4: ECM and Cytoskeleton Organization; Vesicle |
| 323 | 3008 | HRAS | | 5: Actin Cytoskeleton; Cell Communication |
| 260 | 2258 | GOT2 GAPDH | | 6: Carbohydrate, Antioxidant (Mt.), Respiratory Chain Complex (Mt.) |
| 157 | 2487 | | 0.67 | 7: RNA Modification, Spliceosome |
| 108 | 1194 | RPS27A UBA52 | | 8: Proteolysis Ubiquitinylation Proteasome |

D

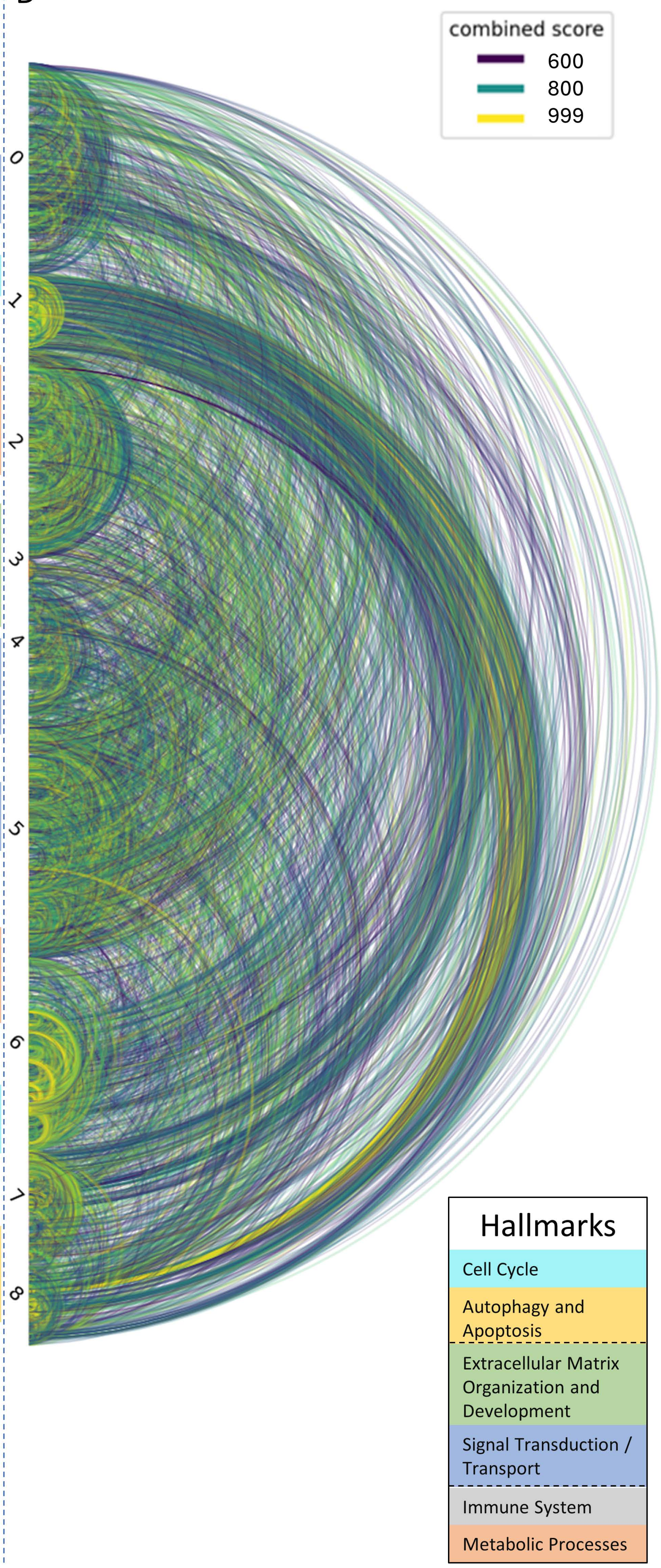

A

Network Properties

| n | e | Mean Reg. Dir. |
|---|---|---|
| 4226 | 66298 | 0.04 |

# HD DEG

B

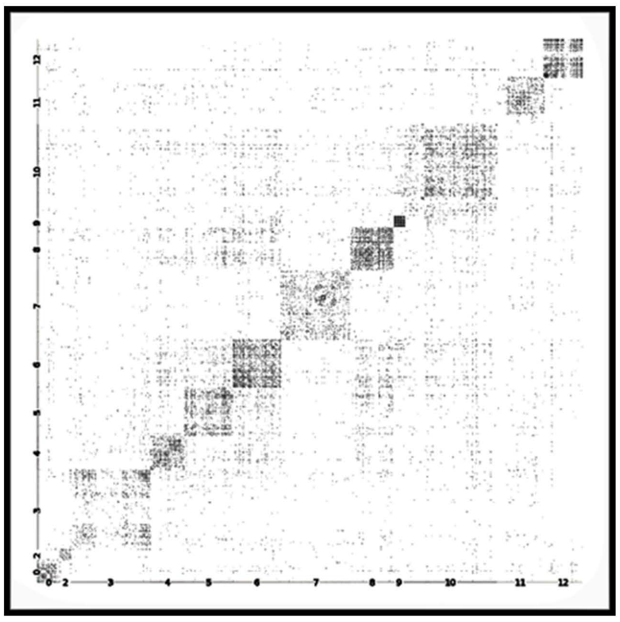

C

| n | e | Hub Genes | Mean Reg Dir | Comm. Number: Associated Terms | | |
|---|---|---|---|---|---|---|
| 172 | 1046 | **HSPA9** HSP90AA1 | -0.16 | 0: | Respiratory Chain Complex (Mt.) | Chaperone Mediated Process |
| 81 | 253 | SLC30A1 | | 2: | Sphingolipid, Homeostasis | |
| 617 | 6729 | GRIA2 | -0.42 | 3: | Ion Channels Synapsis Neurotransmitter Secretion | |
| 262 | 1628 | GNB1 | | 4: | Cell Communication Vesicle | |
| 375 | 2559 | **CDC42** | 0.18 | 5: | Actin Cytoskeleton Cell differentiation and growth | |
| 364 | 5507 | **CD4** TLR4 | 0.55 | 6: | Apoptotic Process | Complement and coagulation cascade |
| 541 | 2929 | PPARA | | 7: | Lipid, Cholesterol, Fatty Acid Oxidation (Mt.), Branched AA Degradation | |
| 332 | 3794 | **FN1** ITGB1 | 0.29 | 8: | Collagen Cell Adhesion | |
| 81 | 687 | | 0.39 | 9: | Ubiquitinylation | Interferon |
| 716 | 6272 | **TP53** **MYC** HDAC1 | 0.17 | 10: | Cell differentiation and growth Early Development | |
| 363 | 1675 | | | 11: | Autophagic Process | Trans-membranal Transport |
| 294 | 3010 | | -0.11 | 12: | Translation RNA Modification | |

D

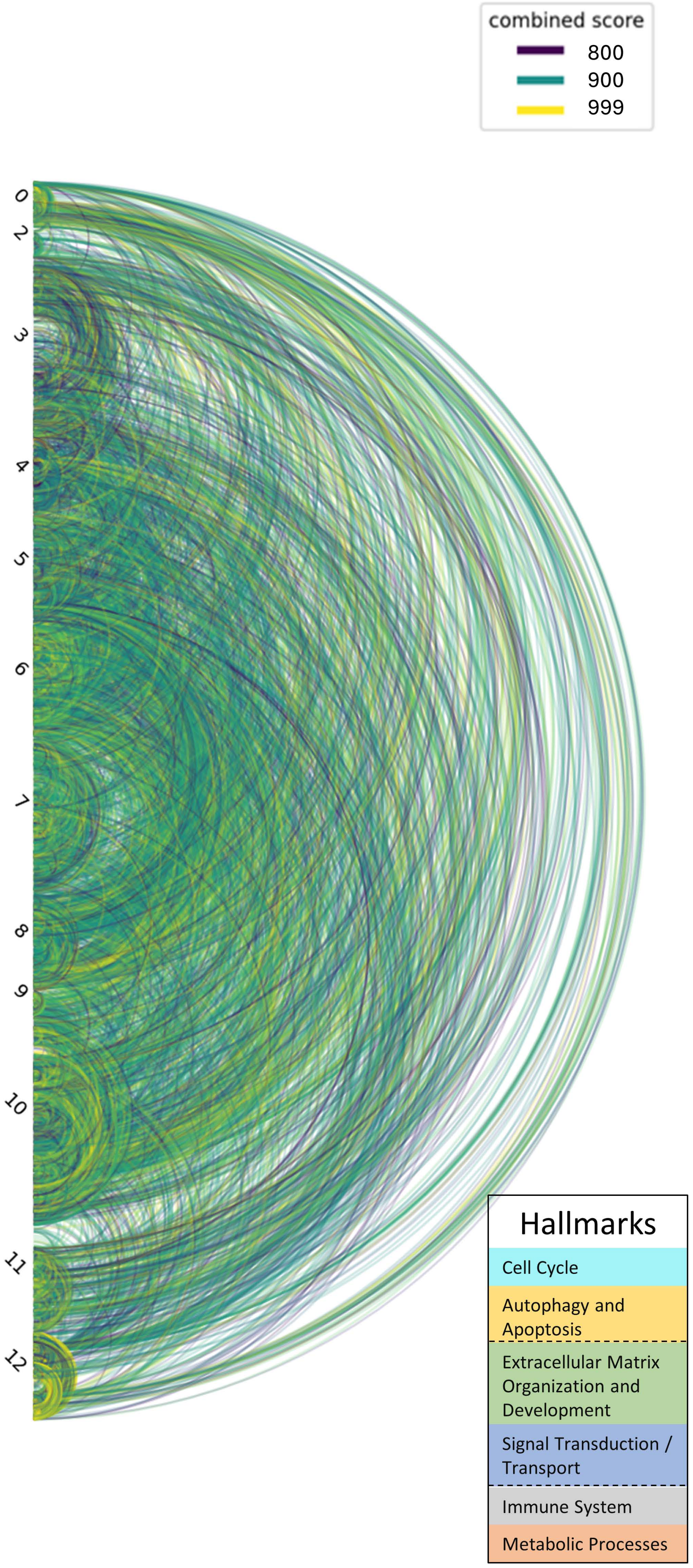

A

Network Properties

| n | e | Mean Reg. Dir. |
|---|---|---|
| 1411 | 19927 | -0.18 |

# HD Prot

B

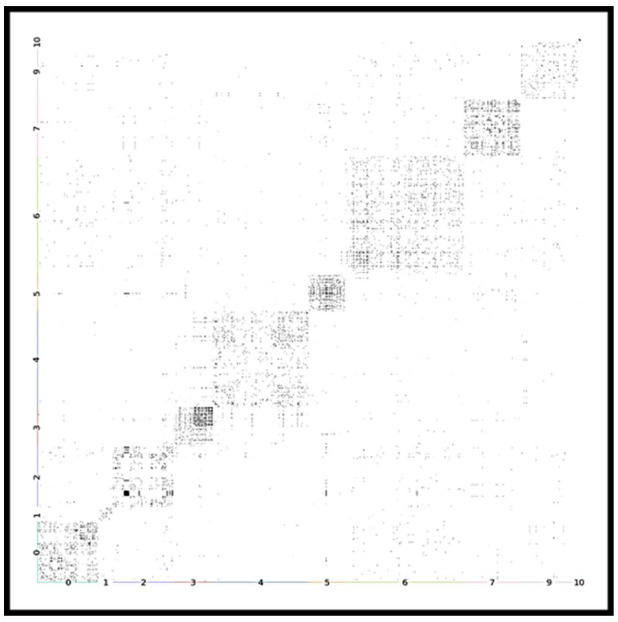

C

| n | e | Hub Genes | Mean Reg Dir | Comm. Number: Associated Terms |
|---|---|---|---|---|
| 157 | 1269 | RAB5A **CLTC** | -0.26 | 0: SNARE Binding Vesicle |
| 36 | 85 | APOC3 CLU | | 1: Complement and coagulation cascade; Lipoprotein |
| 158 | 987 | HSP90AB1 PSMD4 | | 2: Chaperone Mediated Process, Proteasome |
| 97 | 1039 | NDUFS3 | -0.78 | 3: Respiratory Chain Complex (Mt.) |
| 247 | 2415 | ACO2 FH | | 4: Fatty Acid Oxidation (Mt.), Carbohydrate |
| 94 | 808 | | -0.36 | 5: Translation |
| 303 | 2683 | **CDC42** ACTB CTNNB1 | | 6: Actin Cytoskeleton, Cell Adhesion |
| 145 | 1571 | SRSF1 HNRNPK | | 7: RNA Modification, Spliceosome |
| 148 | 978 | DLG4 | -0.27 | 9: Synapsis, Neurotransmitter Secretion |
| 7 | 13 | KRT1 KRT2 KRT5 | **1.0** | 10: Keratin Cytoskeleton |

D

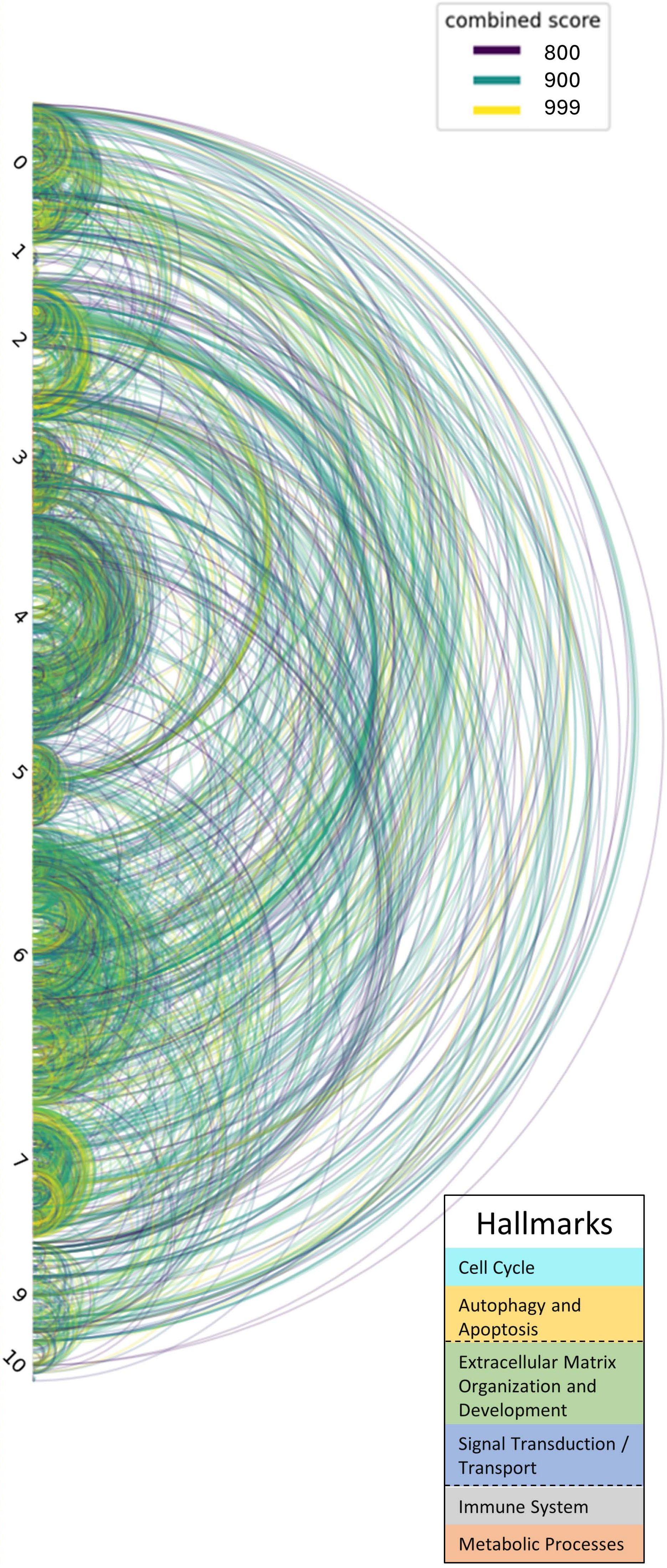

A

Network Properties

| n | e | Mean Reg. Dir. |
|---|---|---|
| 806 | 2862 | -0.80 |

# HD Meth

B

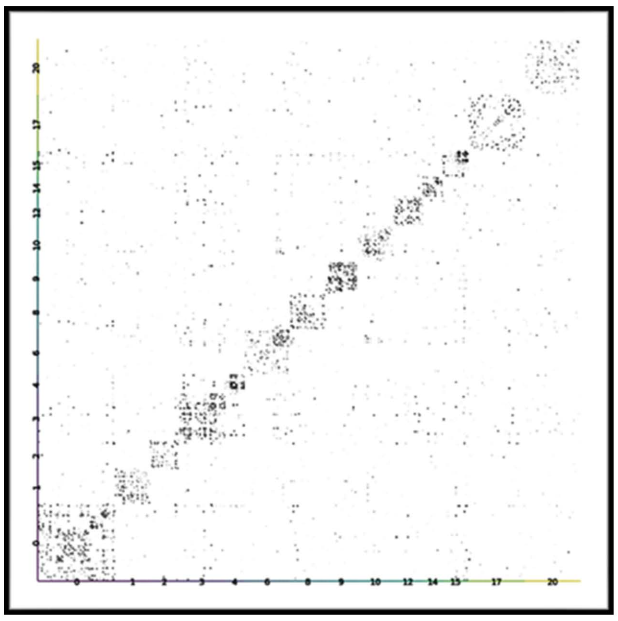

C

| n | e | Hub Genes | Mean Reg Dir | Comm. Number: Associated Terms | |
|---|---|---|---|---|---|
| 112 | 336 | CDK2<br>DNMT1 | -0.74 | 0: | Regulation of Cell Cycle, DNA Repair, Transcription, Chromatin |
| 50 | 83 | **SNRPN**<br>LSM7 | -0.83 | 1: | RNA Modification, Spliceosome |
| 39 | 56 | **PPARGC1A** | -0.67 | 2: | Fatty Acid Oxidation (Mt.), Carboxylic Acid (Mt.), Carbohydrate |
| 67 | 168 | RAF1 | -0.8 | 3: | Cell Communication |
| 28 | 49 | DVL2<br>**LRP5**<br>LRP6 | -0.95 | 4: | Apoptotic Process; Cell differentiation & growth |
| 66 | 136 | **CACNA1C**<br>KCNQ1<br>KCNJ2 | -0.86 | 6: | Ion Channels, Voltage-gated Channel (Cardiac) Muscle Action Potential |
| 52 | 93 | **PAFAH1B1** | -0.78 | 8: | ECM- & cytoskeleton organization; Axonal Transport |
| 45 | 122 | RPS6<br>RPL19<br>RPS15 | -0.67 | 9: | Translation |
| 53 | 83 | **ADCY6**<br>ADCY9<br>**GNAS** | -0.82 | 10: | Cell Communication |
| 42 | 88 | IRF1<br>IRF4 | -0.9 | 12: | Immune Response, Defense Response to Other Organism, Cytokine, Interferon |
| 29 | 53 | NDUFAB1 | -0.76 | 14: | Mitochondrial Translation; Respiratory Chain Complex (Mt.) |
| 37 | 87 | PSMA6<br>USP14<br>PSMA3 | -0.68 | 15: | Ubiquitinylation, Proteasome |
| 82 | 144 | CAV1<br>THBS1 | -0.89 | 17: | ECM- and cytoskeleton organization, Cell Migration |
| 81 | 127 | GAK | -0.79 | 20: | Localization; Trans-Golgi-Network |

D

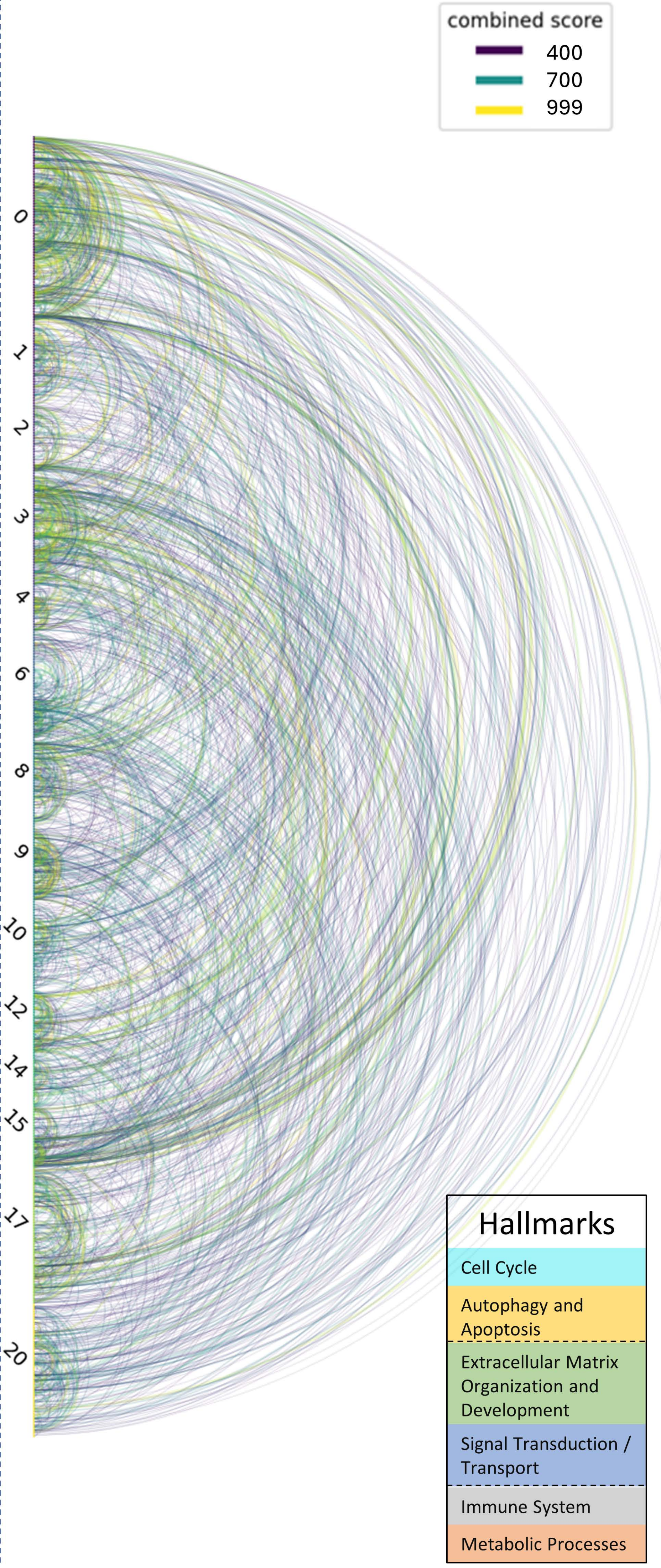

A

# HD SNP

Network Properties

| n | e | Mean Reg. Dir. |
|---|---|---|
| 16 | 13 | n.a. |

| n | e | Hub Genes | Comm. Number: Associated Terms |
|---|---|---|---|

None

B

# ALS Meth

Network Properties

| n | e | Mean Reg. Dir. |
|---|---|---|
| 18 | 12 | -0. 75 |

| n | e | Hub Genes | Mean Reg Dir | Comm. Number: Associated Terms |
|---|---|---|---|---|
| 2 | 1 | * | * | 1: Protein |

C

# ALS SNP

| n | e | Mean Reg. Dir. |
|---|---|---|
| 147 | 214 | n.a. |

| n | e | Hub Genes | Comm. Number: Associated Terms |
|---|---|---|---|
| 20 | 35 | **NRXN3**<br>ANK3 | 1: Cell Adhesion |
| 9 | 9 | **MAT2B**<br>**SQLE**<br>STOX1 | 2: Cholesterol |
| 2 | 1 | * | 6: Spindle Apparatus |
| 6 | 6 | **LAMA3**<br>LAMA2<br>ITGA9 | 19: |
| 5 | 6 | * | 20: Fatty Acid Oxidation (Mt.) Lipid |

| Hallmarks |
|---|
| Cell Cycle |
| Autophagy and Apoptosis |
| Extracellular Matrix Organization and Development |
| Signal Transduction / Transport |
| Immune System |
| Metabolic Processes |
