# Supplementary figures and images for: The Big Picture of Neurodegeneration: A Meta Study to Extract the Essential Evidence on Neurodegenerative Diseases in a Network-Based Approach
Source: Front Aging Neurosci. 2022 Jun 27;14:866886. doi: 10.3389/fnagi.2022.866886 (PMC9271745; doi:10.3389/fnagi.2022.866886)

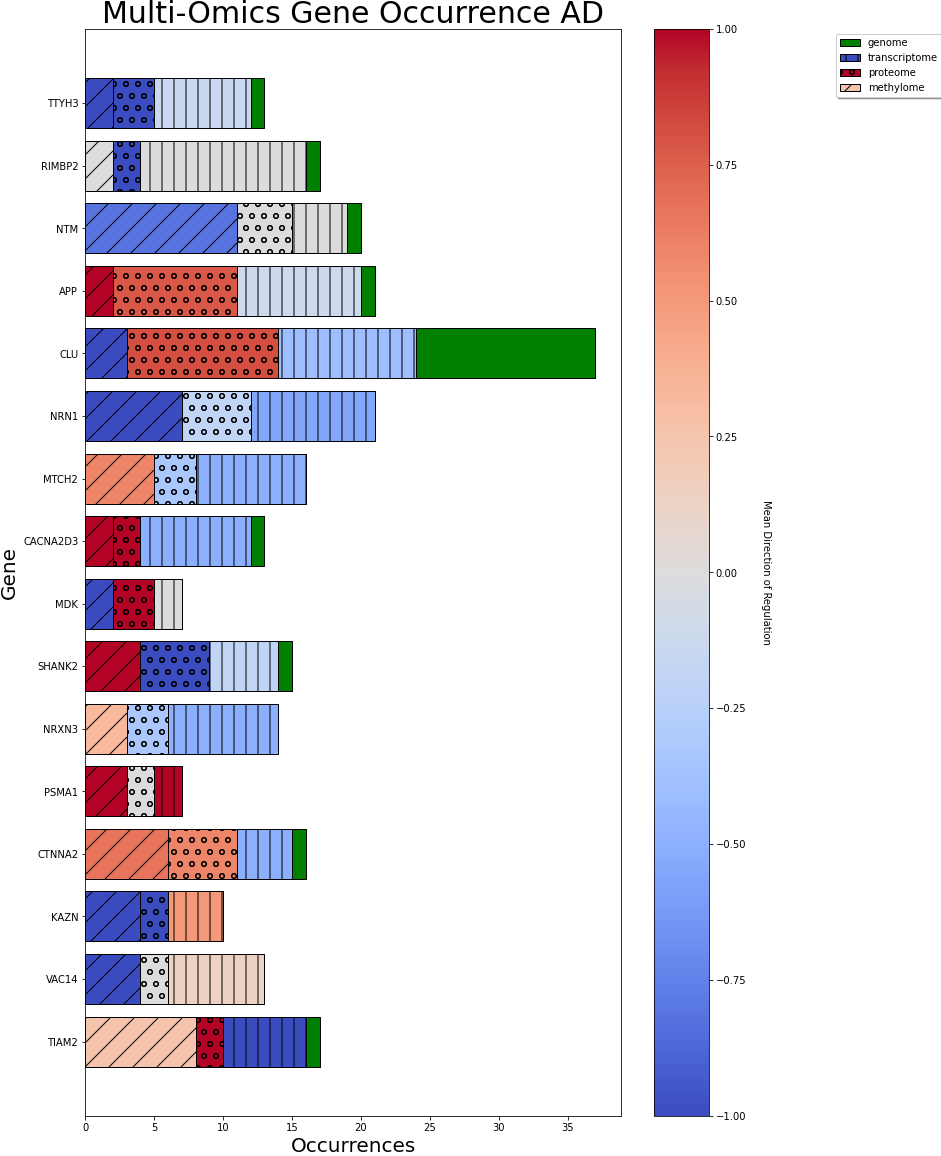

Supplement: Supplementary file 6 [file Presentation_2.zip › StackedBarPlots/AD_four.png]

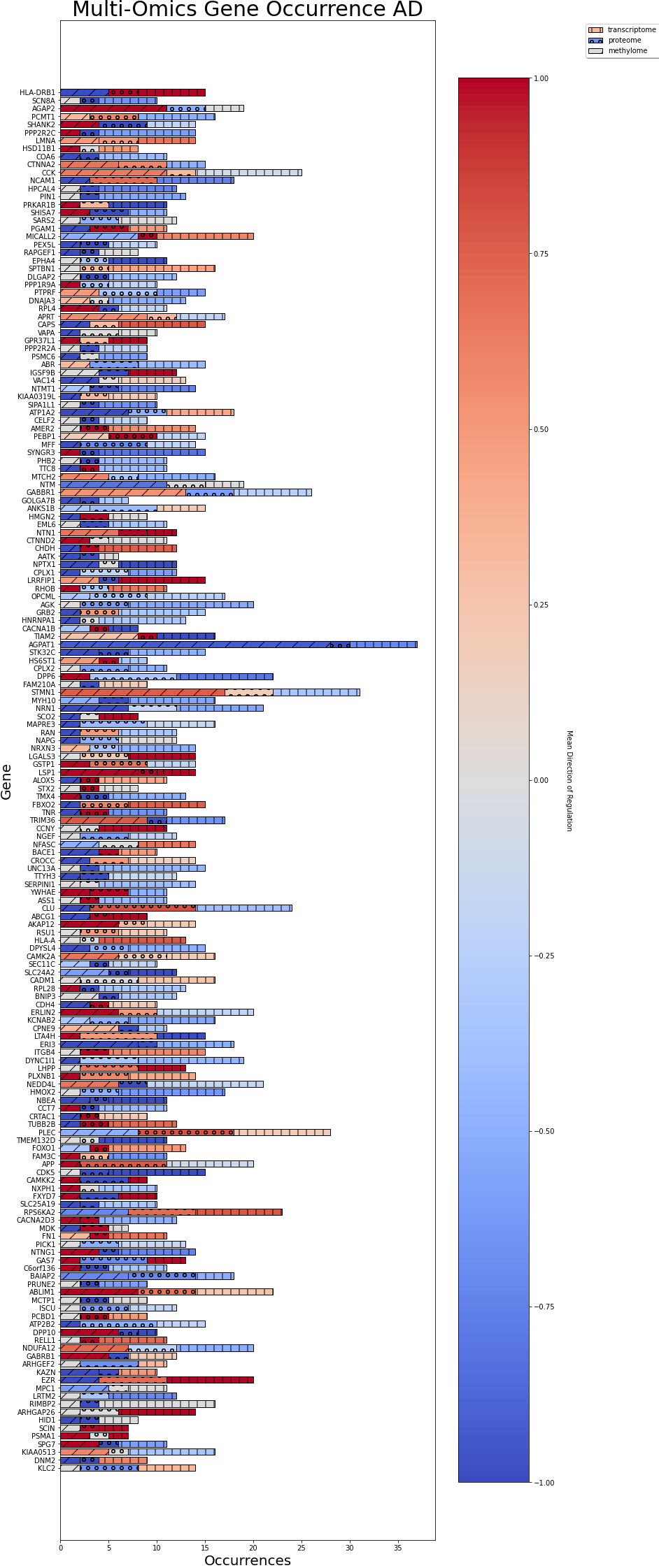

Supplement: Supplementary file 6 [file Presentation_2.zip › StackedBarPlots/AD_noSNP.png]

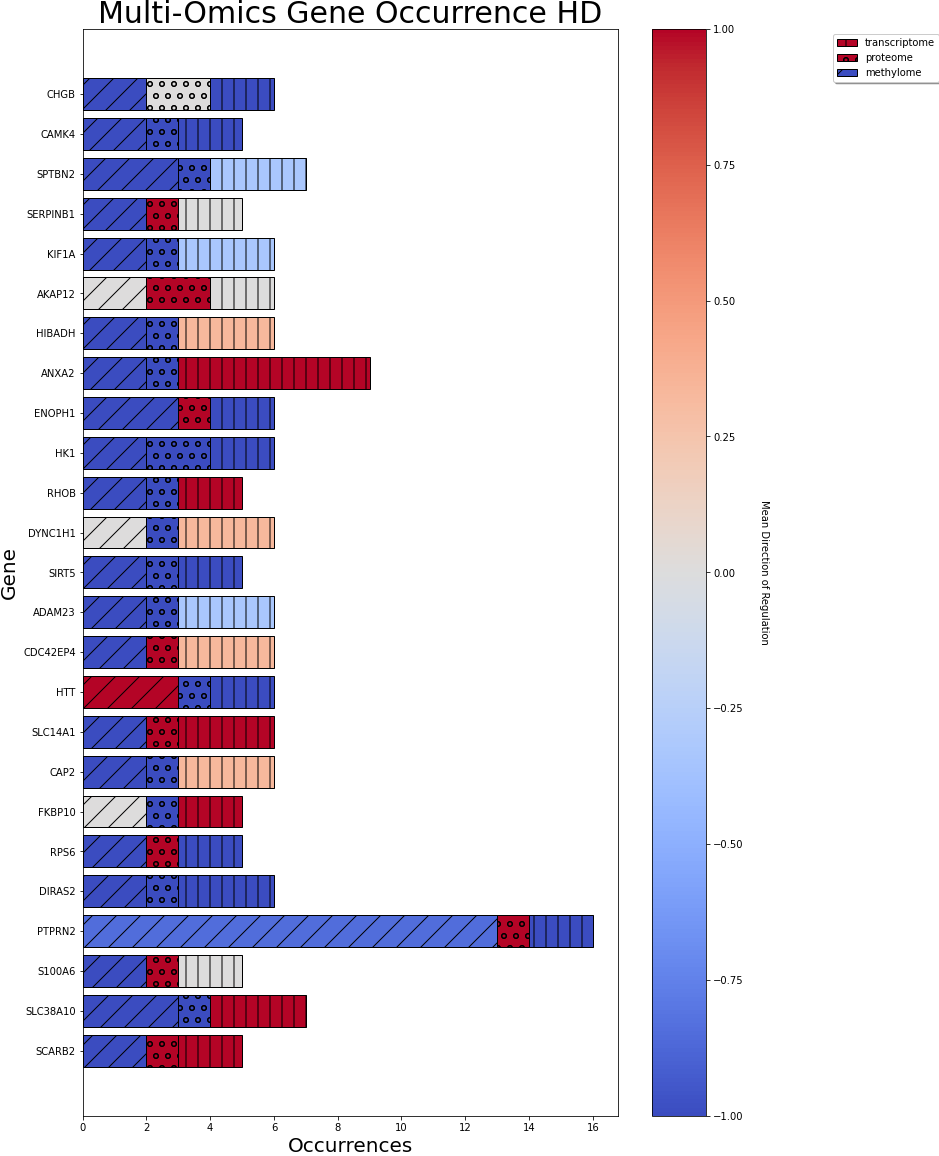

Supplement: Supplementary file 6 [file Presentation_2.zip › StackedBarPlots/HD_noSNP.png]

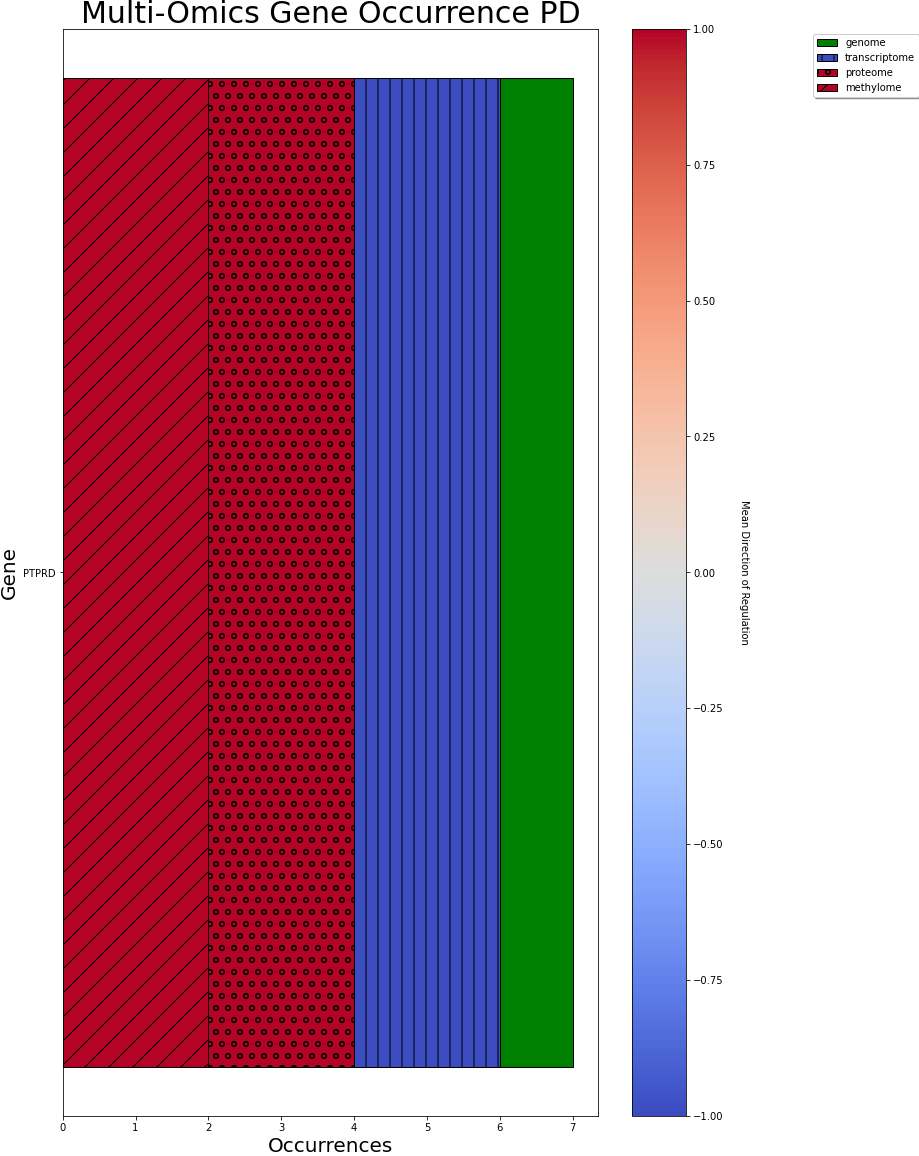

Supplement: Supplementary file 6 [file Presentation_2.zip › StackedBarPlots/PD_four.png]

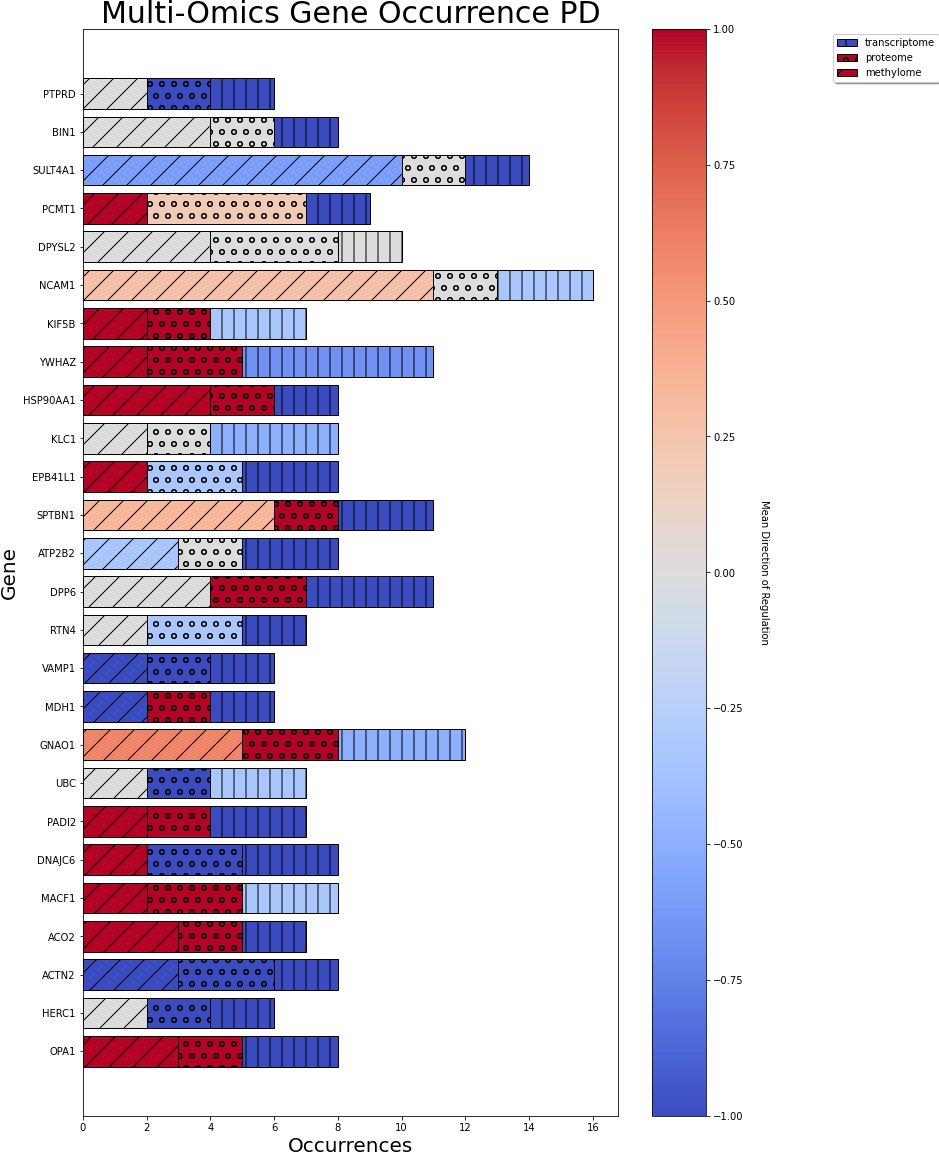

Supplement: Supplementary file 6 [file Presentation_2.zip › StackedBarPlots/PD_noSNP.png]
